# Mutual potentiation of IPA1 and OsNPR1 enhances rice immunity

**DOI:** 10.1093/plcell/koag122

**Published:** 2026-05-20

**Authors:** Zhen Wang, Mingming Liu, Rujuan Yan, Xiaohan Zhang, Xiaoguang Song, Guosheng Xiong, Jiayang Li, Zuhua He, Dong-Lei Yang

**Affiliations:** State Key Laboratory of Crop Genetics & Germplasm Enhancement and Utilization, Nanjing Agricultural University, Nanjing 210095, China; State Key Laboratory of Plant Trait Design, CAS Center for Excellence in Molecular Plant Science, Institute of Plant Physiology and Ecology, Chinese Academy of Sciences, Shanghai 200032, China; State Key Laboratory of Crop Genetics & Germplasm Enhancement and Utilization, Nanjing Agricultural University, Nanjing 210095, China; State Key Laboratory of Crop Genetics & Germplasm Enhancement and Utilization, Nanjing Agricultural University, Nanjing 210095, China; State Key Laboratory of Plant Trait Design, CAS Center for Excellence in Molecular Plant Science, Institute of Plant Physiology and Ecology, Chinese Academy of Sciences, Shanghai 200032, China; State Key Laboratory of Crop Genetics & Germplasm Enhancement and Utilization, Nanjing Agricultural University, Nanjing 210095, China; Yazhouwan National Laboratory, Sanya 572024, China; Plant Phenomics Research Center, Academy of Advanced Interdisciplinary Studies, Nanjing Agricultural University, Nanjing 210095, China; Yazhouwan National Laboratory, Sanya 572024, China; State Key Laboratory of Plant Trait Design, CAS Center for Excellence in Molecular Plant Science, Institute of Plant Physiology and Ecology, Chinese Academy of Sciences, Shanghai 200032, China; State Key Laboratory of Crop Genetics & Germplasm Enhancement and Utilization, Nanjing Agricultural University, Nanjing 210095, China; State Key Laboratory of Plant Trait Design, CAS Center for Excellence in Molecular Plant Science, Institute of Plant Physiology and Ecology, Chinese Academy of Sciences, Shanghai 200032, China

## Abstract

Disease resistance often comes with a penalty in growth and yield. The microRNA miR156 and its target, the transcription factor gene *IDEAL PLANT ARCHITECTURE 1* (*IPA1*), regulate developmental processes such as tillering and panicle branching while enhancing disease resistance and abiotic stress tolerance in rice (*Oryza sativa*). However, how this transcription factor regulates multiple processes remains unclear. Here, we found that IPA1 physically interacts with NON-EXPRESSOR OF PATHOGENESIS-RELATED GENES 1 (OsNPR1) in the nucleus. Under normal conditions, the *OsNPR1* expression levels are low, and OsCULLIN3a (OsCUL3a), an E3 ligase responsible for OsNPR1 degradation, keeps the abundance of monomeric OsNPR1 low in the nucleus and prevents IPA1 from transcriptionally regulating defense genes. When the plant is attacked by pathogens, OsNPR1 oligomers dissociate into monomers, which translocate into the nucleus and physically interact with IPA1, facilitating its binding to promoters of downstream genes, thereby activating positive defense regulators and repressing negative defense regulators. Simultaneously, IPA1 abundance increases, and IPA1 interacts with OsNPR1 and OsCUL3a, interfering with the OsCUL3a–OsNPR1 interaction, dampening the ubiquitin-mediated degradation of OsNPR1. The stabilization of OsNPR1 by IPA1 further enhances IPA1 transcriptional activity in disease resistance. Our work demonstrates that OsNPR1 facilitates IPA1 binding to the promoters of genes related to disease resistance and that IPA1 inhibits OsCUL3a-mediated degradation of OsNPR1.

## Teaser

The physical interaction between IPA1 and OsNPR1in the nucleus both enhances the transcriptional activity of IPA1 in rice immunity and stabilizes OsNPR1 via preventing it from OsCUL3a-mediated degradation.

## Introduction

Salicylic acid (SA) plays a crucial role in plant defense against biotrophic and semi-biotrophic pathogens. NON-EXPRESSOR OF PATHOGENESIS-RELATED GENES 1 (NPR1) is required for SA signaling (Cao et al. 1994, 1997; Ryals et al. 1997; Shah et al. 1997). NPR1 exists in an oligomeric form in the cytoplasm. Upon pathogen infection, the alteration in the cellular redox status causes the reduction of disulfide bonds between individual NPR1 molecules. The released NPR1 monomers then translocate to the nucleus, where they can interact with multiple transcription factors (TFs), such as TGA and TCP family members, to regulate the expression of defense-related genes (Kinkema et al. 2000; Fan et al. 2002; Mou et al. 2003; Li et al. 2018).

In contrast to NPR1, a positive regulator in disease resistance, 2 of its paralogs, NPR3 and NPR4, repress disease resistance in Arabidopsis (Zhang et al. 2006; Ding et al. 2018; Liu et al. 2020). One reason for the opposite role of NPR1 and NPR3/NPR4 in immunity is that NPR3 and NPR4 interact with TGAs, not to activate their transcription activity as NPR1 does but to repress their transcription activity (Ding et al. 2018). Another reason is that NPR3 and NPR4 associate with the Cullin–RING ligase 3 (CRL3) complex and interact with NPR1 to mediate its degradation in the nucleus (Fu et al. 2012). Yeast 2-hybrid (Y2H) assays did not detect any interaction between NPR1 and CULLIN3 (CUL3) in Arabidopsis (Dieterle et al. 2005). However, a high concentration of SA was shown to promote the formation of SA-induced NPR1 condensates (SINCs), in which the NPR1 and CUL3 interaction occurs. The NPR1-CUL3 ligase complex ubiquitinates and degrades proteins involved in cell death and immunity to promote cell survival (Zavaliev et al. 2020).

In rice (*Oryza sativa*), overexpression of *OsNPR1* (also reported as *NPR1 HOMOLOGUE1* [*NH1*]), the closest homolog of *NPR1*, enhances resistance against bacterial blight (Chern et al. 2005; Yuan et al. 2007). As in Arabidopsis, rice OsCUL3a interacts with OsNPR1 and mediates its degradation via the ubiquitin/26S proteasome system (Liu et al. 2017). In contrast to NPR3 as a negative regulator in immunity in Arabidopsis, another NPR1 homolog, OsNPR3/NH3, enhances disease resistance in rice (Bai et al. 2011).

*SQUAMOSA-PROMOTER BINDING PROTEIN-LIKE* (*SPL*) genes encode plant-specific TFs that regulate a wide range of biological responses in plants. Among the 19 *SPL* genes in rice, the transcripts of 11 are cleaved by microRNA 156 (miR156) (Xie et al. 2006). *OsSPL14*, also reported as *IDEAL PLANT ARCHITECTURE 1* (*IPA1*), affects panicle branching, tillering, and culm development (Jiao et al. 2010; Miura et al. 2010; Zhang et al. 2017). IPA1 directly binds to the promoters of *TEOSINTE BRANCHED 1* (*OsTB1*) and *DENSE AND ERECT PANICLE1* (*DEP1*), regulating their transcription to control tillering and panicle branching, respectively (Lu et al. 2013). IPA1 also modulates the levels of reactive oxygen species (ROS) and abscisic acid (ABA) by regulating the transcription of *HOMEOBOX GENE 12* (*OsHOX12*), *NAC DOMAIN-CONTAINING PROTEIN 1* (*OsNAC1*), and *OsNAC52* to enhance drought tolerance (Zhu et al. 2022; Chen et al. 2023). Under cold stress conditions, IPA1 binds to the GTAC motif in the promoter of *C-REPEAT BINDING FACTOR 3* (*OsCBF3*) and increases its transcription (Jia et al. 2022a). Under salinity stress, MITOGEN-ACTIVATED PROTEIN KINASE 4 (OsMPK4) phosphorylates IPA1, promoting its degradation (Jia et al. 2022b). In addition, IPA1 confers resistance against blast disease and bacterial blight (Wang et al. 2018; Liu et al. 2019a). The molecular mechanism underlying this distinct IPA1-mediated biological function, however, is unclear. In this study, we discovered that IPA1 interacts with OsNPR1 and the E3 ligase OsCUL3a, leading to the accumulation of OsNPR1 in the nucleus. In the nucleus, OsNPR1 promotes the binding of IPA1 to the promoters of its target genes to activate disease resistance genes and to repress disease susceptible genes. Our results demonstrated that IPA1 boosts rice immunity through enhancing SA signaling.

## Materials and methods

### Rice plants and growth conditions

The transgenic lines *OsNPR1*-OE (G316), *IPA1*-OE, microRNA156-MIMIC (MIM156), miR156-OE, *OsRDR2-3xFLAG* (*OsRDR2-FLAG*), *IPA1*-RNAi, and *OsWRKY72*-OE (*OsWRKY72*-*3xFLAG*) were described previously (Yuan et al. 2007; Hou et al. 2019; Liu et al. 2019a; Zhang et al. 2021; Wang et al. 2022). The mutants *Osnpr1-3, Osnpr1-4*, *Osnpr1-6*, *Osnpr3-1*, *Osnpr3-2*, *Oswrky45-4*, *Oswrky45-5*, *ipa1-13*, and *Osspl17-1* and the transgenic rice lines *OsCUL3apro:OsCUL3a*-*3xHA* (*OsCUL3a*-*HA*), *OsNPR1pro:OsNPR1-3xFLAG* (*OsNPR1-FLAG*), *Ubi*:*OsNPR1*-*GFP* (*OsNPR1*-*GFP*), *FEM3-3xHA* (*FEM3-HA*), and *IPA1pro:IPA1*-*3xFLAG* (*IPA1*-*FLAG*) were generated in this study. The *Osnpr1 IPA1*-OE, *Osnpr1 IPA1-FLAG*, *Osnpr1* MIM156, *OsWRKY72*-OE *IPA1*-OE, *Osnpr3 IPA1*-OE, *Oswrky45 IPA1*-OE, *IPA1*-OE *OsCUL3a*-*HA*, *IPA1*-OE *OsNPR1-GFP*, and miR156-OE *OsNPR1-FLAG* lines were obtained by crossing and identification of plants with the desired genotypes in the F_2_ and F_3_ generations based on genotyping PCR.

All rice plants were grown in isolated paddy fields in Lingshui (Hainan Province) and Nanjing (Jiangsu Province). The tobacco plants (*N. benthamiana*) were cultivated in a growth chamber under a 12-h light/12-h dark cycle at 23 °C for 4 to 5 wk.

### Plasmid construction and plant transformation

For *OsCUL3apro*:*OsCUL3a*-*3xHA*, a 5,709-bp *OsCUL3a* genomic fragment containing a 2,829-bp promoter and the 2,880-bp coding region without the stop codon was amplified by PCR using Nipponbare genomic DNA as template and primers with flanking sequences matching the vector pCAMBIA1305-3xHA (Supplementary Data Set S5). The *OsCUL3a* DNA fragment was cloned into the pCAMBIA1305-3xHA vector digested with SalI (R3138S, NEB) and HindIII (R3104S, NEB) through homologous recombination using a ClonExpress II One Step Cloning Kit (C112-02, Vazyme). For *FEM3-3xHA* (*FEM3-HA*), a 14,593-bp *FEM3* genomic fragment without the stop codon was amplified by PCR using Nipponbare genomic DNA as template and primers with flanking sequences matching the vector pCAMBIA1305-3xHA (Supplementary Data Set S5). The *FEM3* DNA fragment was cloned into the pCAMBIA1305-3xHA vector digested with SalI (R3138S, NEB) and HindIII (R3104S, NEB) through homologous recombination using a ClonExpress II One Step Cloning Kit (C112-02, Vazyme).

For *IPA1pro*:*IPA1*-*3xFLAG*, a 5,906-bp *IPA1* genomic fragment containing a 2,000-bp promoter and the 3,906-bp coding region without the stop codon was amplified by PCR using Nipponbare genomic DNA as template and primers with flanking sequences matching the vector pCAMBIA1305-3xFLAG (Supplementary Data Set S5). The *IPA1* DNA fragment was cloned into the pCAMBIA1305-3xFLAG vector digested with HpaII (R0171S, NEB) and HindIII (R3104S, NEB) through homologous recombination using a ClonExpress II One Step Cloning kit (C112-02, Vazyme).

For *OsNPR1pro*:*OsNPR1-3xFLAG* (*OsNPR1-FLAG*), a 6,387-bp *OsNPR1* genomic fragment containing a 2,034-bp promoter and the 4,353-bp coding region without the stop codon was amplified by PCR using Nipponbare genomic DNA as template and primers with flanking sequence matching the vector pCAMBIA1305-3xFLAG (Supplementary Data Set S5). The DNA fragment of OsNPR1 was cloned into the pCAMBIA1305-3xFLAG vector, which had been digested with EcoRI (R3101S, NEB) and KpnI (R3142S, NEB) enzymes, using T4 DNA ligase (M0202V, NEB).

For *OsNPR1-GFP*, the coding region of *OsNPR1* without stop codon was amplified by PCR using Nipponbare genomic DNA as template and primers with flanking sequences matching the vector pRTVnGFP (Supplementary Data Set S5). The *OsNPR1* DNA fragment was cloned into the pRTVnGFP vector digested with KpnI (R3142S, NEB) and BamHI (R3136, NEB) through homologous recombination using a ClonExpress II One Step Cloning Kit (C112-02, Vazyme).

To generate mutants, 20-bp single-guide RNAs (sgRNAs) specific for *OsNPR1*, *OsNPR3, OsCUL3a*, *IPA1*, *OsSPL17*, or *OsWRKY45* were designed with the CRISPR2 online tool (http://crispr.hzau.edu.cn/CRISPR2/). The corresponding oligos were annealed and ligated into the pCBSG03 vector digested with BsaI (R0535V, NEB) (Zhang et al. 2014). The resulting constructs were subjected to Sanger sequencing for confirmation and transformed into Nipponbare plants through Agrobacterium (*Agrobacterium tumefaciens*, strain EHA105)-mediated transformation (Horsch et al. 1985). All the primers used in this study are listed in Supplementary Data Set S5.

### Diaminobenzidine (DAB) staining and trypan blue staining

For DAB staining, the leaves from 10-wk-old rice plants were vacuum-inﬁltrated in 1 mg/mL DAB solution (1 g 3,3′-Diaminobenzidine dissolved in 1 L distilled deionized water, pH 3.0) for 60 min and incubated in darkness at 25 °C for 8 h. After staining, the leaves were dipped in de-staining buffer (4:1 (v/v) ethanol:trichloromethane) to completely remove chlorophyll. The cleared leaves were then photographed (Canon EOS 500D). For trypan blue staining, leaves of the same age as those used for DAB staining were boiled in 75% (v/v) ethanol until completely bleached, followed by boiling for 2 min in staining solution (10 mL glycerol, 10 mL lactic acid, 40 mg trypan blue and 10mL water-saturated phenol, 10 mL distilled deionized water). The leaves were then de-stained in 2.5 g/mL chloral hydrate solution and transferred to 70% (v/v) glycerol for imaging.

### Pathogen inoculation

*Xanthomonas oryzae* pv*. oryzae* (*Xoo*) inoculation was conducted by following a previously described (Liu et al. 2019a). Briefly, the *Xoo* strains PXO99A and DY89031 were cultured on nutrient agar (NA) medium (containing 3 g/L beef extract, 1 g/L yeast extract, 5 g/L polypeptone, 10 g/L sucrose, and 15 g/L agar) at 28 °C for 2 d. The bacteria were scraped and resuspended in sterilized water to an OD_600_ = 0.5 to inoculate leaves by the leaf-clipping method. The lesion lengths were measured at 14 d post-inoculation (dpi). For bacterial growth assays, the infected leaves were washed with sterilized water 3 times, homogenized, and resuspended in 2 mL sterilized water; the suspension was then serially diluted and plated on NA medium containing 15 mg/L cephalexin. The plates were incubated at 28 °C for 3 d and then the colony-forming units (CFUs) on the plates were counted.

*Xoo* treatment assays were conducted following a previously reported protocol with minor modifications (Thomas et al. 2016). In brief, leaves of 2-mo-old rice were cut into 1.5-2 cm pieces and immediately floated on 10 mM MgCl_2_ solution for mock treatment or the same solution containing a fresh *Xoo* (DY89031) cell suspension at an OD_600_ = 0.1. The leaves were kept under constitutive light and collected 36 h post-inoculation (hpi) to extract total protein.

For *Magnaporthe oryzae* inoculation, the strain FJ81278 was cultured on straw decoction and corn (SDC) medium (100 g/L rice straw, 40 g/L corn flour and 15 g/L agar) at 28 °C for 5 d and then the surface mycelium was gently removed and incubated for an additional 2 d at 28 °C to facilitate spore collection. The spores were collected in distilled water with 0.02% (v/v) Tween-20 to a titer of 1 × 10^5^ spores/mL. Rice leaves were dissected and punch-inoculated with the above spore solution. The inoculated leaves were kept on water-soaked filter paper in darkness for 24 h at 28 °C and about 90% relative humidity. The inoculated leaves were then shifted to a 12-h light/12-h dark photoperiod and incubated for 7 d before the lesion lengths were measured.

### Yeast one-hybrid (Y1H) assay

The Y1H assay was conducted according to the Matchmaker One-hybrid System's instructions (Clontech). The full-length coding sequence of *IPA1* without the stop codon was amplified by PCR using Nipponbare cDNA as template. The PCR product was cloned into the prey vector pPC86 digested with SalI (R3138S, NEB) and EcoRI (R3101S, NEB). The *OsWRKY72*, *OsWRKY45*, and *OsNPR3* promoter fragments were individually amplified using Nipponbare genomic DNA as template and gene-specific primers (Supplementary Data Set S5) and ligated into the bait plasmid p178 digested with XhoI (R0146S, NEB). The pPC86-*IPA1* plasmid was co-transformed with p178-*OsWRKY72pro*, p178-*OsWRKY45pro*, or p178-*OsNPR3*pro into yeast strain EGY48 via the lithium acetate–polyethylene glycol method (Gietz et al. 2002). Transformants were selected on synthetic defined (SD) medium lacking tryptophan (−Trp) and uracil (−Ura). The yeast was transferred onto SD medium lacking tryptophan (−Trp) and uracil (−Ura) containing X-α-Gal (SL0940, Coolaber), cultivated for 3 days (28 °C), and then photographed.

### Yeast two-hybrid (Y2H) assay

The vectors of IPA1^1–417^-AD, IPA1^1–100^-AD, IPA1^101–185^-AD, IPA1^186–417^-AD, OsSPL17-AD were reported previously (Duan et al. 2019; Liu et al. 2019a; Wang et al. 2021). The coding sequences of *IPA1* (full length), *OsNPR1*^1–582^, *OsNPR1*^1–268^, and *OsNPR1*^269–582^ were individually amplified by PCR and cloned into the pGBKT7 vector (Clontech). The coding sequences of *OsCUL3a*^1–736^, *OsCUL3a*^1–649^, *OsCUL3a*^1–377^, *OsCUL3a*^378–649^, and *OsCUL3a*^650–736^ and the full-length coding sequences of *OsSPL2*, *OsSPL3*, *OsSPL4*, *OsSPL7*, *OsSPL11*, *OsSPL12*, *OsSPL13*, *OsSPL16*, and *OsSPL18* were individually amplified by PCR and cloned into the pGADT7 vector (Clontech). The resulting vectors were co-transformed as appropriate pairs into the yeast strain AH109 via the lithium acetate–polyethylene glycol method (Gietz et al. 2002). The positive transformants were selected on SD −Leu−Trp medium. After incubation in SD −Leu−Trp liquid medium at 30 °C for 3 d, yeast colonies were adjusted to a concentration of OD_600_ = 1. The yeast colonies were then diluted by water in 10-fold serial dilutions and plated onto SD −Leu−Trp−His−Ade medium. The plates were photographed after 3 d of incubation.

### Yeast three-hybrid (Y3H) assay

Y3H assays were conducted according to the guidelines from the MATCHMAKER GAL4 Two-Hybrid System (Clontech) and as described in a previous report with minor modifications (Liu et al. 2019b). For pBridge-OsNPR1-IPA1, the full-length coding sequence of *OsNPR1* was amplified by PCR using Nipponbare cDNA as template and ligated into multiple cloning site I (MCSI) of pBridge vector to generate a fusion to the GAL4 DNA-binding domain (BD). The full-length coding sequence of *IPA1* and *OsSPL3* was amplified by PCR using Nipponbare cDNA as template and ligated into MCS II to make the expression of the gene inducible by the absence of methionine (Met), OsSPL3 served as a control. The full-length coding sequence of *OsCUL3a* was amplified by PCR using Nipponbare cDNA as template and cloned into the pGADT7 vector. The resulting vectors were co-transformed into the AH109 yeast strain and cultured on SD−Leu−Trp medium. After cultivation at 30 °C for 3 days, the yeast cells were resuspended in water were adjusted to an OD_600_ of 1 prior to further analysis and plated onto SD−Ade−Leu−Trp−His medium and SD −Ade−Leu−Trp−His−Met medium. The yeast colonies were imaged 3 d later.

### Luciferase complementation imaging assay

The LUC complementation imaging assay was carried out as previously described (Chen et al. 2008). The full-length coding sequences of *IPA1* and *OsNPR1* were individually amplified by PCR using Nipponbare cDNA as template and cloned into the pCAMBIA1300-nLUC vector using recombinant ligase (C112-02, Vazyme). The full-length coding sequences of *OsNPR1* and *OsCUL3a* were individually amplified by PCR and cloned into the pCAMBIA1300-cLUC vector using recombinant ligase (C112-02, Vazyme). The resulting vectors were transformed into Agrobacterium strain GV3101; positive transformants were grown in liquid LB medium containing 25 µg/mL gentamycin and 50 µg/mL kanamycin for 36 to 48 h. The bacteria were collected by centrifugation (4,000×g, 5 min) and resuspended in infiltration buffer (10 mM MgCl_2_, 10 mM MES and 200 M acetosyringone) to an OD_600_ of 0.5. The indicated combination of bacterial suspensions was mixed (cLUC:nLUC = 1:1, v/v) and infiltrated into the leaves of five-week-old *N. benthamiana* plants. After incubation for 48 h under darkness and 16 h in the light at 23 °C, the *N. benthamiana* leaves were sprayed with 1 mM luciferin (115144-35-9, GoldBio) and the luminescence signals were captured using a CCD imaging system (Night SHADE LB985, Berthold Technologies).

### Bimolecular fluorescence complementation (BiFC) assay

The full-length coding sequence of *IPA1* was amplified by PCR using Nipponbare cDNA as template and cloned into the 35S-SPYNE173 vector (Waadt et al. 2008) to generate *IPA1*-*nYFP*. The full-length coding sequence of *OsNPR1* was amplified by PCR using Nipponbare cDNA as template and cloned into the 35S-SPYCE(M) vector to generate *OsNPR1*-*cYFP*. The resulting constructs were confirmed by Sanger sequencing and transformed into Agrobacterium strain GV3101. The indicated combinations of bacterial suspensions in infiltration buffer were infiltrated into *N. benthamiana* leaves with the nuclear marker construct *D53-eRFP* (Zhou et al. 2013). After 2 days, the fluorescence signals were imaged using a confocal scanning microscope (LSM 800, Zeiss, excitation wavelengths: 488 nm for YFP, and 561 nm for RFP; emission wavelengths: 527 nm for YFP and 610 nm for RFP; one time laser power calibration: 5%; scanning mode: sequential scanning; scan speed: 2 s; Venus: 110 V; offset: 0%; 40×/1.3 NA water-immersion objective). To examine the effect of BTH on the interaction between IPA1 and OsNPR1, 200 µM BTH or a mock solution was infiltrated into *N. benthamiana* leaves 12 h prior to imaging. For quantitative analysis, at least 10 fields per sample were imaged with identical settings, and fluorescence intensity was quantified using ImageJ.

### Antibody development

The antibodies against OsNPR1 and OsWRKY45 were produced by ABclonal Technology (Wuhan, China). For the production of the anti-OsNPR1 antibody, a DNA fragment encoding amino acid residues (aa) 475 to 582 was cloned into the pET32a vector through homologous recombination using a ClonExpress II One Step Cloning kit (C112-02, Vazyme). The resulting construct was transformed into *E. coli* Rosetta strain BL21. For the production of the anti-OsWRKY45 antibody, a DNA fragment encoding the C-terminal half of OsWRKY45 (180 to 326 aa) was cloned into the pET28a vector through homologous recombination using a ClonExpress II One Step Cloning kit (C112-02, Vazyme) and further transformed into *E. coli* Rosetta strain BL21. Positive colonies were cultivated in liquid LB containing 50 mg/L kanamycin, and protein production was induced by the addition of 0.8 mM IPTG (isopropyl-β-d-thiogalactoside) at 37 °C for 4 h. The collected bacteria were broken by sonication (Vibra cell VCX-750, Sonics & Materials, Inc., Newton, USA). The recombinant protein was isolated using Glutathione Sepharos 4B beads (17-0756-01, GE Healthcare). This purified protein was used as antigen to immunize a rabbit, which received 4 injections at 12-d intervals. The antibody against OsNPR1 and OsWRKY45 were purified from the rabbit serum.

### Protein extraction and immunoblotting

Total proteins were extracted with lysis buffer (50 mM Tris-HCl pH 7.5, 150 mM NaCl, 1% [v/v] Triton X-100, 0.1% [w/v] SDS, 1 mM EDTA, 3 mM dithiothreitol, 1 mM PMSF, 1% [w/v] protease inhibitor) on ice for 20 min. The supernatants were collected after centrifugation at 12,000 × g for 10 min at 4 °C. Proteins were separated in 8 to 10% (w/v) SDS-PAGE gel and then transferred to polyvinylidene difluoride (PVDF) membrane (IPVH00010, Millipore).

Immunodetection of OsNPR1, IPA1, OsWRKY45, and OsNPR1*-*GFP were performed using anti-OsNPR1 antibody (1:5,000), anti-IPA1 antibody (1:5,000, AbP80307-A-SE, BPI), anti-OsWRKY45 antibody (1:5,000) or anti-GFP antibody (1:4,000, AE012, ABclonal), respectively, and a secondary antibody (1:5,000, CW0156, CWbio; goat anti-rabbit IgG, horseradish peroxidase conjugated). Immunodetection of OsWRKY72*-*3xFLAG, IPA1*-*3xFLAG, OsCUL3a-3xFLAG, IPA1-3xHA, and OsCUL3a-3xHA was performed using an anti-FLAG antibody (1:2,000, F1804, Sigma-Aldrich), anti-HA antibody (1:3,000, H3663, Sigma-Aldrich), anti-actin antibody (1:5,000, CW0264A, CWBIO) and anti-histone H3 antibody (1:2,000, 05-499, Millipore), respectively, and a secondary antibody (1:5,000, CW0102S, CWbio; goat anti-mouse IgG, horseradish peroxidase conjugated). Signals were detected with an ECL Western Blotting Detection Kit (IF6747, Engibody) under a ChemiDoc XRS gel imaging system (Bio-Rad Laboratories, Hercules). Immunodetection of actin and histone H3 or Ponceau S staining (0.1 g Ponceau S and 5 mL acetic acid dissolved in 100 mL ddH_2_O) of the Rubisco large subunit served as protein loading controls.

### Cell-free protein degradation assay

Cell-free degradation assays were performed essentially as described (Spoel et al. 2009). Briefly, protein was extracted in lysis buffer (25 mM Tris-HCl pH 7.5, 10 mM MgCl_2_, 10 mM NaCl and 5 mM DTT) on ice for 20 min. The extracts were centrifuged at 12,000 × g for 10 min at 4 °C, and the supernatants were transferred to 200 μL tubes and incubated at 28 °C. At the indicated times (0 min, 20 min, 40 min, 60 min), 2× SDS sample buffer was added to the samples and heated at 95 °C for 5 min to terminate the degradation reaction. OsNPR1 protein abundance was analyzed via immunoblotting.

### Nuclear-cytoplasmic fractionation

Cytoplasmic and nuclear proteins were isolated as previously described (Wang et al. 2011). Briefly, 1 g of leaves was ground and resuspended in lysis buffer (20 mM Tris-HCl pH 7.5, 20 mM KCl, 2 mM EDTA, 2.5 mM MgCl_2,_ 25% [v/v] glycerol, 0.25 M sucrose, 1 mM PMSF, 5 mM DTT, and 1% [w/v] protease inhibitor). The lysate containing the total proteins was filtered through 3 layers of Miracloth (475855-1R, Millipore) and then centrifuged at 12,000 × g for 10 min at 4 °C. The supernatants containing cytoplasmic proteins were collected. The pellet, containing nuclei and organelles, was washed 3 times with 1.5 mL NRBT buffer (20 mM Tris-HCl pH 7.5, 25% [v/v] glycerol, 2.5 mM MgCl_2_ and 0.2% [v/v] Triton X-100). The pellet was then resuspended in 600 μL NRB2 buffer (20 mM Tris-HCl pH 7.5, 10 mM MgCl_2_, 0.25 M sucrose, 0.5% [v/v] Triton X-100, 5 mM DTT, 1 mM PMSF and 1% [w/v] protease inhibitor). The obtained suspension was layered onto NRB3 buffer (20 mM Tris-HCl pH 7.5, 10 mM MgCl_2_, 1.7 M sucrose, 0.5% [v/v] Triton X-100) in the ratio of 1:1 (v/v) and centrifuged at 12,000 × g for 30 min at 4 °C. The nuclear pellet was resuspended in 200 µL of protein lysis buffer. Samples were separated by SDS-PAGE. Actin and histone H3 served as cytoplasmic and nuclear markers, respectively.

### Co-immunoprecipitation (co-IP) analysis

Total proteins were extracted with co-IP lysis buffer (50 mM HEPES, pH 7.5, 150 mM NaCl, 0.5% [v/v] Triton X-100, 5% [v/v] glycerol, 1 mM EDTA, 1 mM DTT and 1% [w/v] protease inhibitor) on ice for 30 min. After centrifugation at 10,000 × g for 10 min at 4 °C, the supernatant was incubated with 5 μL anti-FLAG (F1804-5MG, Sigma-Aldrich) or anti-HA (H3663, Sigma-Aldrich) antibody at 4 °C for 2 h with gentle rotation. Then 10 μL rProtein A Sepharose Fast Flow (17127902, Cytiva) was added, and the mixture was incubated for 3 h. The immunoprecipitated protein complexes were centrifuged at 5,000 × g for 2 min at 4 °C and washed with Phosphate Buffered saline (PBS) buffer at least 3 times and analyzed by immunoblotting using an anti-HA antibody (H3663, Sigma-Aldrich), anti-FLAG antibody (F1804-5MG, Sigma-Aldrich), anti-OsNPR1 antibody, or anti-IPA1 antibody (AbP80307-A-SE, BPI). For BTH treatment, plants were treated with 200 µM BTH for 12 h, and then total protein was extracted using co-IP lysis buffer.

### Electrophoretic mobility shift assays

The full-length coding regions of IPA1 (Duan et al. 2019) and OsNPR1 were separately amplified by PCR using Nipponbare cDNA as template and cloned into the pGEX-4T-1 vector using recombinant ligase (C112-02, Vazyme). The fusion proteins IPA1–GST and OsNPR1–GST were expressed in *Escherichia coli* BL21 (DE3). IPA1–GST and OsNPR1–GST were purified using GSTSep Glutathione Agarose Resin (20507ES10, Yeasen). DNA probes were designed on the specific binding motifs of *IPA1*-Probe A in *OsWRKY72* promoter, Probe B and Probe C in *OsNPR3* promoter, Probe D in *OsWRKY45* promoter. Oligonucleotide probes were synthesized and labeled with Cy5 at the 5′ end by Sangon (Shanghai, China). The oligos information are provided in the Table S5.

Electrophoretic mobility shift assays (EMSAs) were performed in vitro as follows. Purified recombinant protein (0.5 μg) was incubated with 1 μL of 5 μM Cy5-labeled DNA probe in a 20 μL reaction mixture containing 2 μL of 10× binding buffer (50 mM Tris-HCl, pH = 7.6; 10 mM NaCl; 250 mM KCl; 25 mM MgCl_2_; 10 mM EDTA, pH = 8.0; and 10 mM dithiothreitol) and 4 μL of 50% (v/v) glycerol. The reaction mixtures were incubated at 28 °C for 1 h in the dark. Subsequently, 2 μL of 10× loading buffer was added, and the samples were separated by 6% native polyacrylamide gel electrophoresis in 0.5× TBE buffer at 100 V for 80 min on ice in the dark. Cy5-labeled DNA signals were detected using a Starion FLA-9000 imaging system (Fujifilm, Japan).

### Gene expression assay and RNA-seq analysis

For gene expression assays, total RNA was extracted from leaves using TRIzol reagent, following the manufacturer's protocol (15596018, Invitrogen). About 500 ng total RNA was used to synthesize first-strand cDNA using HiScript Q RT SuperMIX (R233-01, Vazyme). Quantitative PCR was performed using AceQ qPCR RT SYBR Green Master Mix (Q212-01, Vazyme) on a real-time PCR detection system (Bio-Rad, CFX96) using *UBIQUITIN* (LOC_Os03g13170) as internal control.

For RNA-seq analysis, total RNA was extracted from the leaves of NIP, *IPA1*-OE, *Osnpr1-3 IPA1*-OE, TP309, and *OsNPR1*-OE plants with TRIzol reagent (15596018, Invitrogen), using the leaves from 5 individual rice plants as 1 replicate, each genotype with 3 replicates. The libraries were prepared with NEBNext Ultra RNA Library Prep kit for Illumina (E7770, NEB). The mRNA was enriched by magnetic beads with oligo dT and fragmented into short pieces by fragmentation buffer, The enriched mRNA served as templates for the synthesis of the first strand of cDNA. The synthesis of double-stranded DNA was completed by adding buffer, dNTPs, RNase H, and DNA polymerase I. The resulting DNA was purified using AMPure XP beads. Following purification, the DNA was end-repaired, A-tailed, and ligated with adapters. The final library was produced through PCR enrichment. And the library was sequenced on an Illumina NovaSeq 6000 platform (Annoroad Gene Technology Co., Ltd., Beijing, China).

Raw reads were cleaned using Trim_Galore to remove the adaptor sequences and low-quality reads. The “rmdup” command of SAMtools v1.7 was used to discard PCR duplicates (Li et al. 2009). The resulting clean reads were mapped to the Nipponbare reference genome (RGAP 7.0) using Hisat 2 (v2.1.2) (Kim et al. 2019) and StringTie v1.3.4 (Pertea et al. 2015) with default parameters. Transcript abundance was normalized using the number of fragments per kilobase of transcript per million mapped reads (FPKM). The identification of differentially expressed genes (DEGs) was performed with FDR < 0.05 and absolute value of log_2_(fold change) ≥ 0.485 by using the R package DESeq2 (Love et al. 2014). An analysis of GO term enrichment used the Gene Ontology Enrichment Analysis tool: ShinyGO v0.60 as described previously (Ge et al. 2020). Heatmaps, scatterplots and volcano plots were generated using the pheatmap and ggplot2 R packages (Wickham 2011; Kolde 2019).

### Benzothiadiazole (BTH) treatment

BTH (32820-100MG, Sigma) was dissolved in 50 mL of ethanol to prepare a 100 mM stock solution. The fully expanded leaves from 1-mo-old rice plants were then cut into 6-cm segments and immediately floated on mock solution or 200 μM BTH for 24 h, as described in a previous report (Thomas et al. 2016). After 24 h treatment, *Magnaporthe oryzae* inoculation was performed. The inoculated leaves were kept under a 12-h light/12-h dark photoperiod for 10 d before measuring lesion lengths. The experiment was repeated 3 times.

For measuring mRNA or protein levels, 1-mo-old rice seedlings were transferred to nutrient solutions containing 200 μM BTH for the indicated time. Total RNA or protein was then extracted for further analysis.

### ChIP-qPCR

ChIP assays were conducted following a previously reported protocol with minor modifications (Saleh et al. 2008). Briefly, 2-wk-old rice seedlings were collected and the roots were removed. The remaining shoots were cut into pieces. Roughly, 5-g samples were vacuum-infiltrated with cross-linking buffer (0.4 M sucrose, 10 mM Tris–HCl pH 8.0, 1 mM PMSF, 1 mM EDTA, and 1% formaldehyde) for approximately 15 min until the tissue became translucent. The cross-linking reaction was terminated by adding glycine to a final concentration of 0.125 M, and the samples were washed 3 times with distilled water. Cell nuclei were isolated by grinding the samples into fine powder in liquid nitrogen and lysing and washing with nuclei isolation buffer (0.25 M sucrose, 15 mM PIPES pH 6.8, 5 mM MgCl_2_, 60 mM KCl, 15 mM NaCl, 1 mM CaCl_2_, and 0.9% Triton X-100). After centrifugation at 2,000 × g for 5 min, the nuclei pellet was resuspended in nuclei lysis buffer (50 mM HEPES pH 7.5, 150 mM NaCl, 1 mM EDTA, 1% SDS, 1% Triton X-100, 1% [w/v] protease inhibitor) for 15 min. Chromatin was fragmented into 200 to 500 bp fragments by sonication using the Bioruptor Pico Sonication System (B01060003, Diagenode) for 7 to 8 cycles (30 sec on, 30 sec off per cycle). The fragmented chromatin was centrifuged at 12,000 × g for 10 min to remove insoluble debris. Four percent of the diluted chromatin served as the input control. The sonicated chromatin sample was incubated overnight at 4 °C with or without anti-FLAG antibody (F1804, Sigma-Aldrich). Then, 10 μL of rProtein A Sepharose Fast Flow (17127902, Cytiva) pre-equilibrated with nuclei lysis buffer was added and incubated for 3 h. The samples were centrifuged at 3,800 × g for 2 min at 4 °C to collect the beads with the chromatin. Wash the beads for 2 min each time with gentle rotation at 4 °C with 1 mL of each of the following buffers and centrifuge for 3,800 × g for 2 min at 4 °C: first time with low salt wash buffer (20 mM Tris–HCl pH 8.0, 150 mM NaCl, 2 mM EDTA, 1% Triton X-100, 0.1% SDS), the second time with high salt buffer (20 mM Tris–HCl pH 8.0, 500 mM NaCl, 2 mM EDTA, 1% Triton X-100, 0.1% SDS), the third time with LiCl buffer (20 mM Tris–HCl pH 8.0, 250 mM LiCl, 1 mM EDTA, 0.5% NP-40), and other 2 times with TE buffer (1 mM EDTA, 10 mM Tris–HCl pH 8.0). Then, add 500 μL elution buffer (0.5% SDS, 0.1 M NaHCO_3_), 20 μL of 5 M NaCl was added to each tube and incubated at 65 °C overnight to reverse the cross-linking. Add 10 μL of 0.5 M EDTA, 20 μL of 1 M Tris–HCl (pH 6.5), and 1 μL of proteinase K (EO0491, Thermo Fisher scientific) to each tube and incubate at 45 °C for 1.5 hours to digest the proteins. Dissolve the DNA in 100 μL TE buffer (10 mM Tris–HCl pH 8.0, 1 mM EDTA) for qPCR analysis. The primers used for ChIP-qPCR are listed (Supplementary Data Set S5).

### Dual-luciferase reporter assay

Dual-luciferase reporter assays were performed essentially as described (Hellens et al. 2005). The full-length coding sequences of *IPA1* and *OsNPR1* were individually amplified by PCR and cloned into the pGreenII-62-SK vector to generate pGreenII-62-SK-*IPA1* and pGreenII-62-SK-*OsNPR1* through homologous recombination using a ClonExpress II One Step Cloning kit (C112-02, Vazyme). The sequenced-verified constructs were transformed into Agrobacterium strain GV3101.

Promoter fragments of *OsWRKY72* (1,000 bp), *OsWRKY45* (2,540 bp), and *OsNPR3* (3,000 bp) were individually amplified by PCR from Nipponbare genomic DNA and cloned into the pGreenII0800-LUC vector through homologous recombination using a ClonExpress II One Step Cloning Kit (C112-02, Vazyme). The sequenced-verified constructs were transformed into Agrobacterium strain GV3101 harboring the helper plasmid pSoup (G6039-2, AngYuBio).

Agrobacterium harboring a recombinant vector were separately collected by centrifugation at 4,000 × g for 5 min and resuspended in infiltration buffer (10 mM MgCl_2_, 10 mM MES, and 200 µM acetosyringone) to an OD_600_ of 0.4. Agrobacterium cell suspensions carrying a single reporter vector (pGreenII0800-*OsWRKY72pro:LUC*, pGreenII0800-*OsWRKY45pro:LUC*, or pGreenII0800-*OsNPR3pro:LUC*) were mixed in equal volumes with effector construct (pGreenII-62-SK-*IPA1*, pGreenII-62-SK-*OsNPR1*, pGreenII-62-SK). The Agrobacterium mixed suspension was incubated at room temperature for 3 h, before being infiltrated into *N. benthamiana* leaves using a needleless syringe. Two days after infiltration, firefly luciferase (F-LUC) and Renilla luciferase (R-LUC) activities were measured with a Dual Luciferase Reporter Gene Assay Kit (RG027, Beyotime) on a SpectraMax iD5 reader (Molecular Devices). LUC activity was normalized to that of REN.

### Protoplast isolation and dual-luciferase reporter assay

Protoplast isolation from rice was performed as previously described (Zhang et al. 2011) with minor modifications. Briefly, two-week-old seedlings were finely chopped into ∼1 mm fragments and incubated in enzyme solution (1.5% [w/v] Cellulase RS, 0.75% [w/v] Macerozyme R-10, 0.6 M mannitol, 10 mM MES pH 5.7, 10 mM CaCl_2_, and 0.1% [w/v] BSA). The digestion was carried out at 70 × g for 5 h at room temperature. An equal volume of W5 solution (154 mM NaCl, 125 mM CaCl_2_, 5 mM KCl, and 2 mM MES, pH 5.7) was then added to release the protoplasts, which were filtered through a 40 μm nylon mesh. The filtrate was centrifuged at 800 × g for 2 min, and the pellet was resuspended in 300 μL MMG buffer (0.4 M mannitol, 15 mM MgCl_2_, and 4 mM MES, pH 5.7). For transfection, 20 μg of plasmid DNA (pGREENII-0800-LUC, *OsNPR3pro*-LUC, *OsWRKY45pro*-LUC, or *OsWRKY72pro*-LUC) was mixed with an equal volume of PEG solution (40% [w/v] PEG 4000, 0.2 M mannitol, and 0.1 M CaCl_2_). After mixing gently, the suspension was incubated in the dark for 20 min, followed by centrifugation to remove the supernatant. The transformed protoplasts were resuspended in 1 mL WI solution (0.5 M mannitol, 20 mM KCl and 4 mM MES at pH 5.7), and incubated in the dark for 18 h at room temperature. Then, cells were lysed with lysis buffer, and firefly luciferase (F-LUC) and Renilla luciferase (R-LUC) activities were measured using a Dual-Luciferase Reporter Gene Assay Kit (RG027, Beyotime) on a SpectraMax iD5 reader (Molecular Devices) according to the manufacturer's instructions. LUC activity was normalized to REN activity. All plasmids for protoplast transfection were purified using an EndoFree Maxi Plasmid kit (TIANGEN, DP117) according to the manufacturer's instructions.

## Results

### IPA1 physically interacts with OsNPR1

**Figure 1:**
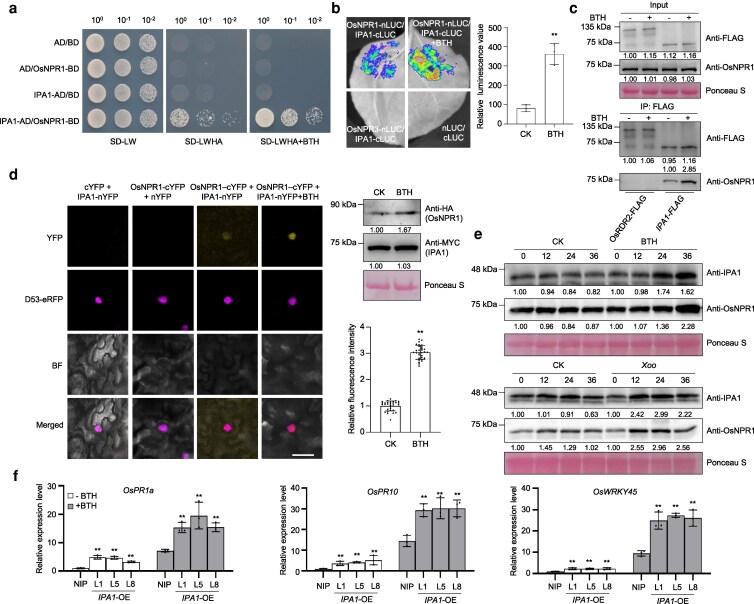
IPA1 physically interacts with OsNPR1 in the nucleus. a) Y2H assay showing the interaction between IPA1 and OsNPR1 in the presence and absence of 200 μM BTH; various control combinations are also indicated. Abbreviations: AD, GAL4 activation domain; A, adenine; BD, GAL4 DNA-binding domain; BTH, benzothiadiazole; H, histidine; L, leucine; SD, synthetic defined medium; W, tryptophan. b) Firefly LUC complementation imaging assay showing that IPA1 and OsNPR1 interact in *N. benthamiana* leaves with and without 200 μM BTH treatment for 12 h, IPA1-OsNPR3 was used as a negative control. The relative luminescence values were quantified. Values are means ± standard deviation (n = 3), ** indicates significant differences (*P* < 0.01) by 2-tailed Student *t* test. c) co-IP assay of the IPA1–OsNPR1 interaction using *IPA1pro*:*IPA1*-*3xFLAG* transgenic rice plants with and without 200 μM BTH treatment for 12 h. *OsRDR2pro*:*OsRDR2*-*3xFLAG* served as negative control. OsNPR1 was detected by immunoblotting with a specific antibody generated for this study. d) BiFC assay of the interaction between IPA1 and OsNPR1 in *N. benthamiana* leaves with and without 200 μM BTH treatment for 12 h and the fluorescence intensity was measured (n = 30). The protein levels of OsNPR1-cYFP (anti-HA) and IPA1-nYFP (anti-MYC) were measured by immunoblotting. D53-eRFP was used as a nuclear marker. Abbreviations: BF, bright field; cYFP, C-terminal half of yellow fluorescent protein; eRFP, enhanced red fluorescent protein; nYFP, N-terminal half of YFP. Scale bar = 50 μm. Values are means ± standard deviation (n = 30), ** indicates significant differences (*P* < 0.01) by 2-tailed Student *t* test. e) Immunoblot analysis of IPA1 and OsNPR1 in 1-mo-old NIP seedlings treated with 200 μM BTH and immunoblotting of IPA1 and OsNPR1 in 2-mo-old NIP leaves treated with *Xoo* (DY89031) at OD_600_ = 0.1. f) Relative transcript levels of *OsPR1a*, *OsPR10*, and *OsWRKY45* in 1-mo-old NIP, *IPA1*-OE seedlings with mock treatment and 200 μM BTH treatment for 12 h. Gene expression levels were examined and normalized to *OsUBQ*. Values are means ± standard deviation (n = 3). ** indicates significant differences (*P* < 0.01) by 2-tailed Student *t* test. All experiments were repeated at least 3 times.

Adult rice plants from 3 *IPA1*-overexpression (*IPA1*-OE) lines grown in a paddy field exhibited a lesion-mimic phenotype on their lower leaves (Fig. S1a). Trypan blue staining revealed widespread cell death in these leaves (Fig. S1b), concomitantly with a pronounced accumulation of H_2_O_2_ as indicated by diaminobenzidine (DAB) staining (Fig. S1c). Consistent with our previous findings (Liu et al. 2019a), 2 marker genes of SA signaling, *PATHOGENESIS-RELATED 1a* (*OsPR1a*) and *OsWRKY45* showed significantly higher expression levels in *IPA1*-OE leaves than in the leaves of the wild-type (WT) progenitor Nipponbare (NIP) (Fig. S1d). The autoimmunity phenotypes of *IPA1*-OE were reminiscent of *OsNPR1*-OE in rice plants (Chern et al. 2005; Yuan et al. 2007) (Fig. S1, e to h). Moreover, both *IPA1*-OE and *OsNPR1*-OE plants exhibited reduced plant height and tiller number (Fig. S1, i to k). The transcript levels of IPA1 were significantly more elevated in *OsNPR1*-OE than in WT plants (Fig. S1l). Together, these results raised the possibility that IPA1 and OsNPR1 function in the same signaling pathway, possibly via direct interaction. To test this hypothesis, we performed a Y2H assay. Only yeast cells harboring constructs encoding IPA1 fused to the activation domain of yeast GAL4 (IPA1-AD) and OsNPR1 fused to the GAL4 DNA-binding domain (OsNPR1-BD) grew well on selective medium lacking leucine, tryptophan, histidine, and adenine (Fig. 1a). Given that OsNPR1 is a putative receptor of SA, we investigated whether SA modulates the interaction between IPA1 and OsNPR1 in Y2H assay. Treatment with the SA functional analog benzothiadiazole (BTH) significantly enhanced the growth of yeast co-transformed with IPA1-AD and OsNPR1-BD (Fig. 1a). A firefly luciferase (LUC) complementation imaging assay in *Nicotiana benthamiana* (*N. benthamiana*) leaves confirmed the interaction of IPA1 with OsNPR1, but not with OsNPR3. Similarly, BTH treatment significantly increased the LUC signal of IPA1-OsNPR1 (Fig. 1b). We generated transgenic *IPA1pro*:*IPA1-FLAG* rice plants expressing *IPA1*-*FLAG* under the *IPA1* native promoter to conduct co-immunoprecipitation (co-IP) assays using an anti-FLAG antibody, which showed that OsNPR1 is present in the immunoprecipitated (Fig. 1c). The precipitation of OsNPR1 was further enhanced in *IPA1*-FLAG with BTH treatment (Fig. 1c). The 3 experiments above therefore demonstrate the interaction between IPA1 and OsNPR1, which was enhanced by SA. A bimolecular fluorescence complementation (BiFC) assay in *N. benthamiana* plants confirmed the IPA1–OsNPR1 interaction and indicated that the 2 proteins interact in the nucleus (Fig. 1d). We also found that BTH significantly enhanced the fluorescence signal in the nucleus (Fig. 1d). The quantification of protein levels and fluorescence signals confirmed that BTH strengthens their interaction (Fig. 1d), which is consistent with the result in co-IP assay (Fig. 1c). As the application of BTH, the inoculation of *Xanthomonas oryzae* pv*. oryzae* (*Xoo*) enhanced the protein abundance of OsNPR1 and IPA1 in rice plants (Fig. 1e). In WT plants (NIP), the transcript levels of *OsPR1a*, *OsPR10*, and *OsWRKY45* were upregulated by BTH treatment as indicated by quantitative reverse transcription-polymerase chain reaction (RT-qPCR). The mRNA abundance of the 3 genes in *IPA1*-OE rice plants was significantly higher than that in WT both with and without BTH treatment (Fig. 1f), indicating that IPA1 boosts SA signaling, possibly due to its physical interaction with OsNPR1. These experiments suggested the key role of IPA1 in the SA signaling pathway.

As *IPA1* is a member of the *OsSPL* family of TFs, whose encoding transcripts are targets of miR156, we tested 10 other OsSPLs with OsNPR1 in the Y2H assay. As with IPA1, OsSPL4, OsSPL7, OsSPL11, and OsSPL17 interacted with OsNPR1 in yeast cells (Fig. S2a), consistent with previous findings that OsSPL4, OsSPL7, and OsSPL17 increase disease resistance (Liu et al. 2019a; Zhang et al. 2022; Hui et al. 2023).

To delineate the interaction interface between IPA1 and OsNPR1, IPA1 was divided into 3 parts—the N terminus (amino acids [aa] 1 to 100), the SQUAMOSA PROMOTER BINDING PROTEIN domain (SBP, aa 101 to 185), and the C terminus (aa 186 to 417)—for Y2H assay to test their interactions with OsNPR1 (Fig. S2b). The N terminus and C terminus of IPA1 interacted with OsNPR1 in this assay (Fig. S2c). Reciprocally, we divided OsNPR1 into 2 parts: the N terminus (aa 1 to 268) containing the broad complex, tramtrack, bric-a-brac/poxvirus and zinc finger (BTB/POZ) domain and the C terminus (aa 269 to 582) containing the ankyrin-repeat (ANK) domain (Fig. S2b). A Y2H assay demonstrated that the BTB domain of OsNPR1, but not the truncation containing the ANK domain, interacts with full-length IPA1 and either end, but not with the SBP domain (Fig. S2c).

### OsNPR1 is required for IPA1-mediated disease resistance

**Figure 2:**
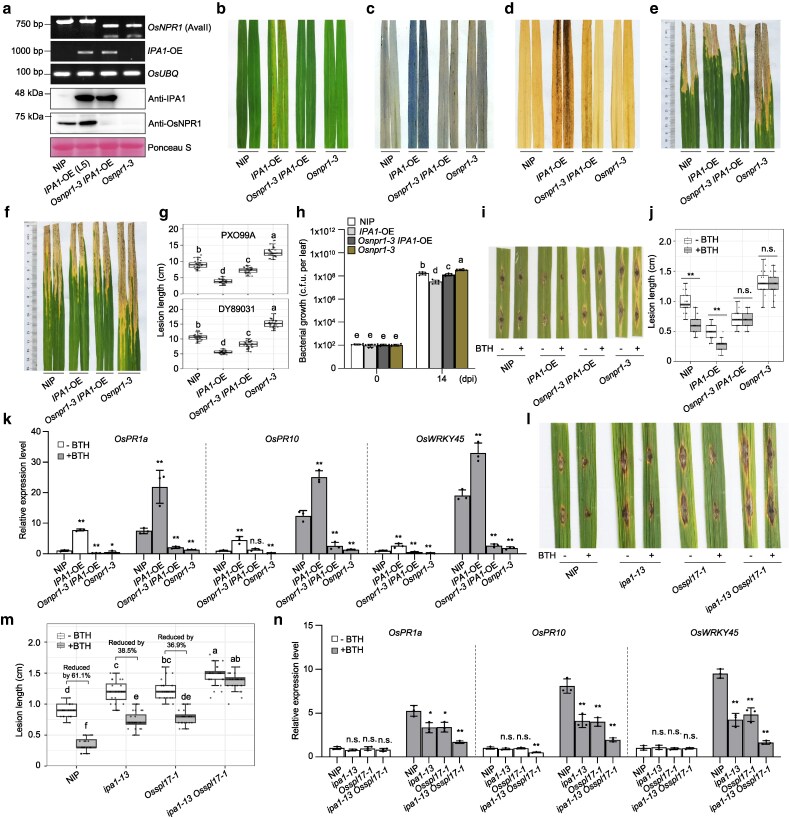
OsNPR1 is required for IPA1-mediated resistance. a) Genotyping PCR for *Osnpr1-3* by Cleaved Amplified Polymorphic Sequences (CAPS) marker and the *IPA1*-OE transgene (top) and immunoblot analysis of OsNPR1 and IPA1 protein abundance (bottom) in the indicated genotypes. *OsUBQ* (LOC_Os03g13170) served as control for the PCR. Ponceau S staining of the Rubisco large subunit served as a loading control for the immunoblot. b–d) Lesion-mimic phenotypes (b), cell death as determined by trypan blue staining (c) and accumulation of reactive oxygen species (ROS) based on diaminobenzidine (DAB) staining (d) in the leaves of NIP, *IPA1*-OE (L5), *Osnpr1-3 IPA1*-OE, and *Osnpr1-3* plants at flowering time. e, f) Lesions caused by bacterial blight in the indicated genotypes at 14 days post inoculation (dpi) with *Xoo* strain PXO99A (e) and DY89031 (f). g) Length of lesions in the indicated genotypes at 14 dpi with *Xoo* strain PXO99A (top) or DY89031 (bottom) (n = 30). h) Bacterial growth, defined as colony-forming units (CFUs), in the indicated genotypes at 0 and 14 dpi (n = 6). i) Blast disease symptoms at 10 dpi of leaves inoculated with *M. oryzae* strain FJ81278 using the punch-inoculated method with or without 200 μM BTH treatment. j) Length of lesions caused by blast disease at 10 dpi with strain FJ81278 (n = 20) with or without 200 μM BTH treatment. k) Relative transcript levels for *OsPR1a*, *OsPR10*, and *OsWRKY45* in the indicated genotypes (n = 3) with mock treatment and 200 μM BTH treatment for 12 h. l) Blast disease symptoms at 10 dpi of NIP, *ipa1-13*, *Osspl17-1*, and *ipa1-13 Osspl17-1* leaves inoculated with *M. oryzae* strain FJ81278 using the punch-inoculated method with or without 200 μM BTH treatment. m) Length of lesions caused by blast disease at 10 dpi with strain FJ81278 (n = 20) with or without 200 μM BTH treatment. n) Relative transcript levels for *OsPR1a*, *OsPR10*, and *OsWRKY45* in the indicated genotypes with mock treatment and 200 μM BTH treatment for 12 h. Gene expression levels in (k) and (n) were examined and normalized to *OsUBQ*. Values are means ± standard deviation (n = 3). Boxplots in (g), (j) and (m) show median and interquartile range, and the error bars denote the full range excluding outliers. Different lowercase letters in (g), (h) and (m) indicate significant differences according to Duncan's new multiple range test (*P* < 0.05). In (j), (k) and (n), asterisks indicate significant differences (* *P* < 0.05, ** *P* < 0.01), n.s. indicates non-significant by 2-tailed Student *t* test. All experiments were repeated at least 3 times.

To reveal the biological significance of the IPA1–OsNPR1 interaction, we generated 3 *Osnpr1* mutants via clustered regularly interspaced short palindromic repeats (CRISPR)/CRISPR-associated nuclease 9 (Cas9)-mediated gene editing. The *Osnpr1-3* mutant carries a 2-bp deletion in the second exon, leading to the introduction of a premature stop codon (Fig. S3a). The mutation in *OsNPR1* does not affect the expression levels of *IPA1* (Fig. S3b). We crossed the *Osnpr1-3* mutant to the *IPA1*-OE line L5 to obtain *Osnpr1-3 IPA1*-OE plants (Fig. 2a). The spontaneous cell death and H_2_O_2_ accumulation phenotypes seen in the *IPA1*-OE line L5 were eliminated in *Osnpr1-3 IPA1*-OE (Fig. 2, b to d). Following manual inoculation of leaf tips with a bacterial suspension for the 2 *Xoo* strains, PXO99A and DY89031, we observed significantly longer lesions due to bacterial blight in *Osnpr1-3 IPA1*-OE leaves than in those of *IPA1*-OE (Fig. 2, e to g). In agreement with this result, PXO99A bacterial populations accumulated to more than 4-fold higher levels in *Osnpr1-3 IPA1*-OE leaves than in *IPA1*-OE leaves (Fig. 2h). The increased resistance against blast disease (*M. oryzae* infection) conferred by *IPA1* overexpression was also compromised in *Osnpr1-3 IPA1*-OE plants (Fig. 2, i and j). The transcript levels of *OsPR1a*, *OsPR10*, and *OsWRKY45* were significantly lower in *Osnpr1-3 IPA1*-OE plants than those in *IPA1*-OE plants (Fig. 2k). A genetic analysis with 2 other *IPA1*-OE lines, L1 and L8, with the other mutant *Osnpr1-6* (carrying a 1-bp deletion at the same target site as *Osnpr1-3* and accumulating no full-length OsNPR1) showed that the disease resistance due to overexpression of *IPA1* is diminished when *OsNPR1* is mutated (Fig. S3c to k; Supplementary Data Set S1). The above evidence suggests that OsNPR1 is required for IPA1-mediated resistance against bacterial blight and blast disease.

The BTH treatment dramatically activated disease resistance against *M*. *oryzae* in WT and *IPA1*-OE. The inducible effect on disease resistance by BTH was completely abolished in *Osnpr1-3 IPA1*-OE plants as well as in *Osnpr1-3* (Fig. 2, i and j). More importantly, the upregulation of *OsPR1a*, *OsPR10*, and *OsWRKY45* by BTH treatment was compromised in *Osnpr1-3 IPA1*-OE plants (Fig. 2k). These results demonstrated that IPA1 boosts SA signaling, probably via OsNPR1.

To reveal the exact role of IPA1 in SA signaling transduction, using 1 sgRNA we generated a loss-of-function double mutant in *IPA1* and its close homolog *OsSPL17*, *ipa1-13 Osspl17-1*. For *IPA1*, 1 bp was inserted in *ipa1-13*, whereas for *OsSPL17*, an 8-bp deletion and 6-bp substitution happened in *Osspl17-1*. Both mutations resulted in a premature stop codon and presumably produced a truncated protein (Fig. S4, a and b). The *ipa1-13 Osspl17-1* was crossed with WT, the single mutants of *ipa1-13* and *Osspl17-1* were segregated out in F2 generation (Fig. S4c). The *ipa1-13* and *Osspl17-1* had more tillers than NIP, and the *ipa1-13 Osspl17-1* double mutant further increased tiller number (Fig. S4d), suggesting that the 2 genes redundantly negatively regulate tillering. Upon BTH treatment, lesion lengths of blast disease were reduced by less than 40% in *ipa1-13* and *Osspl17-1*, which was significantly lower than 61% in NIP. For *ipa1-13 Osspl17-1,* the treatment of BTH did not reduce lesion length (Fig. 2, l and m). Consistently, BTH-induced upregulation of *OsPR1a*, *OsPR10*, and *OsWRKY45* in *ipa1-13* and *Osspl17-1* was partially compromised than in NIP. The reduction extent of the 3 genes under BTH treatment in *ipa1-13 Osspl17-1* was more obvious (Fig. 2n). These results suggested that IPA1 and its closest homolog OsSPL17 redundantly play a vital role in the SA signaling pathway.

Upregulation of *IPA1* resulted in shorter plant height and fewer tillers (Zhang et al. 2017; Liu et al. 2019a). These developmental phenotypes, however, were not affected by the mutation of *OsNPR1* (Fig. S5, a to c; Supplementary Data Set S1). The greater seed size of *IPA1*-OE than WT was more obvious in *Osnpr1 IPA1*-OE, likely due to the negative role of *OsNPR1* in seed size (Fig. S5, d to h; Supplementary Data Set S1), indicating that IPA1 and OsNPR1 antagonistically regulate seed development. Together, these results revealed that IPA1-regulated disease resistance, but probably not development, depends on OsNPR1 and SA signaling.

### OsNPR1 is required for disease resistance in miR156 MIMIC rice plants

**Figure 3:**
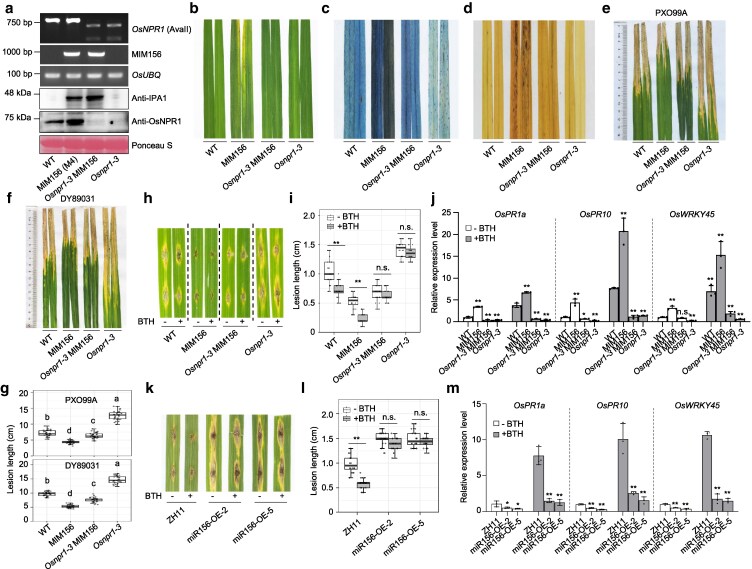
Mutation in *OsNPR1* compromises disease resistance in MIM156. a) Genotyping PCR for the miR156-MIMIC transgene and *Osnpr1-3* by CAPS marker (top) and immunoblot analysis of OsNPR1 and IPA1 protein abundance (bottom) in the indicated genotypes. *OsUBQ* served as control for the PCR. Ponceau S staining of the Rubisco large subunit served as a loading control for the immunoblot. b–d) Lesion-mimic phenotypes (b), cell death as determined by trypan blue staining (c) and accumulation of reactive oxygen species (ROS) based on diaminobenzidine (DAB) staining (d) in the indicated genotypes at the flowering stage. e, f) Lesions caused by bacterial blight in the indicated genotypes at 14 dpi inoculated with *Xoo* strain PXO99A (e) and DY89031 (f). g) Length of lesions in the indicated genotypes at 14 dpi inoculated with *Xoo* strain PXO99A (top) or DY89031 (bottom) (n = 30). Different lowercase letters indicate significant differences (*P* < 0.05) according to Duncan's new multiple range test. h) Blast disease symptoms at 10 dpi in leaves inoculated with *M. oryzae* strain FJ81278 using the punch-inoculated method with or without 200 μM BTH treatment. i) Length of lesions in the indicated genotypes at 10 dpi with strain FJ81278 (n = 20), with or without BTH treatment. j) RT-qPCR analysis of *OsPR1a*, *OsPR10*, and *OsWRKY45* expression level in the indicated genotype plants with mock treatment and 200 μM BTH treatment for 12 h. Gene expression levels were examined and normalized to *OsUBQ*. Values are means ± standard deviation (n = 3). k) Blast disease symptoms at 10 dpi in leaves inoculated with *M. oryzae* strain FJ81278 using the punch-inoculated method with or without 200 μM BTH treatment. l) Length of lesions in the indicated genotypes at 10 dpi with strain FJ81278 (n = 20), with or without BTH treatment. m) RT-qPCR analysis of *OsPR1a*, *OsPR10*, and *OsWRKY45* expression level in the indicated genotype plants with mock treatment and 200 M BTH treatment for 12 h. Gene expression levels were examined and normalized to *OsUBQ*. Values are means ± standard deviation (n = 3). Boxplots in (g), (i) and (l) show median and interquartile range, and error bars denote the full range excluding outliers. In (i), (j), (l) and (m), asterisks indicate significant differences (* *P* < 0.05, ** *P* < 0.01), n.s. indicates non-significant by 2-tailed Student *t* test. All experiments were repeated at least 3 times.

Since *IPA1* is 1 of 11 target genes whose transcripts are cleaved by miR156 (Xie et al. 2006) and OsNPR1 physically interacts with 5 of these OsSPLs (Fig. S2a), we investigated the genetic relationship between OsNPR1 and miR156 in disease resistance and development. The *Osnpr1-3* mutant was introduced into the miR156 MIMIC (MIM156) line, in which miR156 abundance was reduced by the expression of a complementary RNA mimic decoy upregulating its targeted *OsSPLs* (Dai et al. 2018) to generate *Osnpr1-3* MIM156 lines (Fig. 3a). Similar autoimmunity phenotypes, including spontaneous cell death and H_2_O_2_ accumulation, which were observed in *IPA1*-OE and *OsNPR1*-OE leaves, were also detected in MIM156. There was a substantial suppression of spontaneous cell death and H_2_O_2_ accumulation in the *Osnpr1-3* MIM156 plants compared with the MIM156 plants (Fig. 3, b to d). The bacterial blight and blast disease were more severe in *Osnpr1-3* MIM156 than in MIM156 (Fig. 3, e to i), suggesting OsNPR1 is indispensable for miR156 to repress disease resistance. BTH-induced resistance against blast disease was completely abolished in the *Osnpr1-3* MIM156 rice plants (Fig. 3, h and i). The transcript levels of *OsPR1a*, *OsPR10*, and *OsWRKY45* in MIM156 were significantly higher than those in WT both with and without BTH treatment. More importantly, the upregulation of *OsPR1a*, *OsPR10*, and *OsWRKY45* by BTH treatment was almost totally lost in *Osnpr1-3* MIM156 plants (Fig. 3j). In miR156-OE plants, where targeted *OsSPLs* were downregulated, the BTH-enhanced disease resistance was totally lost (Fig. 3, k and l). The upregulation of *OsPR1a*, *OsPR10*, and *OsWRKY45* by BTH treatment was substantially reduced in miR156-OE plants than in WT (Fig. 3m). These results indicated that miR156 negatively regulated SA signaling.

Plant height and tiller number in *Osnpr1-3* MIM156 were comparable to that in MIM156 plants (Fig. S6, a to c). The seeds of *Osnpr1-3* MIM156 were obviously longer than those of MIM156 (Fig. S6, d to h), suggesting that for seed length and seed size, miR156 and OsNPR1 probably function in separate pathways. These morphological phenotype analyses demonstrated that OsNPR1 plays a small role in miR156-regulated development.

We tested the phenotypes using another *OsNPR1* mutant, *Osnpr1-4* (Fig. S3a), and the MIM156 line M13 to obtain *Osnpr1-4* MIM156 (Fig. S7a). The autoimmunity phenotypes seen in MIM156 were largely suppressed in *Osnpr1-4* MIM156 (Fig. S7, b to d). In parallel, the severity of bacterial blight (*Xoo* infection) and blast disease (*M. oryzae* infection) was significantly greater in *Osnpr1-4* MIM156 than in MIM156, although neither reached the levels in *Osnpr1-4* (Fig. S7, e to i). These results demonstrate that the disease resistance, but probably not development, regulated by miR156 partially relies on SA and OsNPR1.

### OsNPR1 promotes the transcriptional activity of IPA1

In Arabidopsis, NPR1 interacts with multiple TGA TFs and functions as a co-transcription factor (Zhang et al. 1999; Zhou et al. 2000; Rochon et al. 2006; Ding et al. 2018; Chen et al. 2021; Khan et al. 2022). Since OsNPR1 interacts with IPA1 and is required for IPA1-mediated disease resistance, we speculated that OsNPR1 might function as a co-transcription factor of IPA1. To test this hypothesis, we conducted transcriptome deep sequencing (RNA-seq) using total RNA extracted from the leaves of NIP, *IPA1*-OE (line L5), *Osnpr1-3 IPA1*-OE, *OsNPR1*-OE (line G316 in the TP309 background), and TP309. Each genotype comprised 3 biological replicates, with each replicate providing over 33 million reads with more than 34× coverage (Supplementary Data Set S2). The scatterplots and Pearson correlation coefficients between replicates demonstrated strong reproducibility among the 3 biological replicates for each genotype (Fig. S8a).

**Figure 4:**
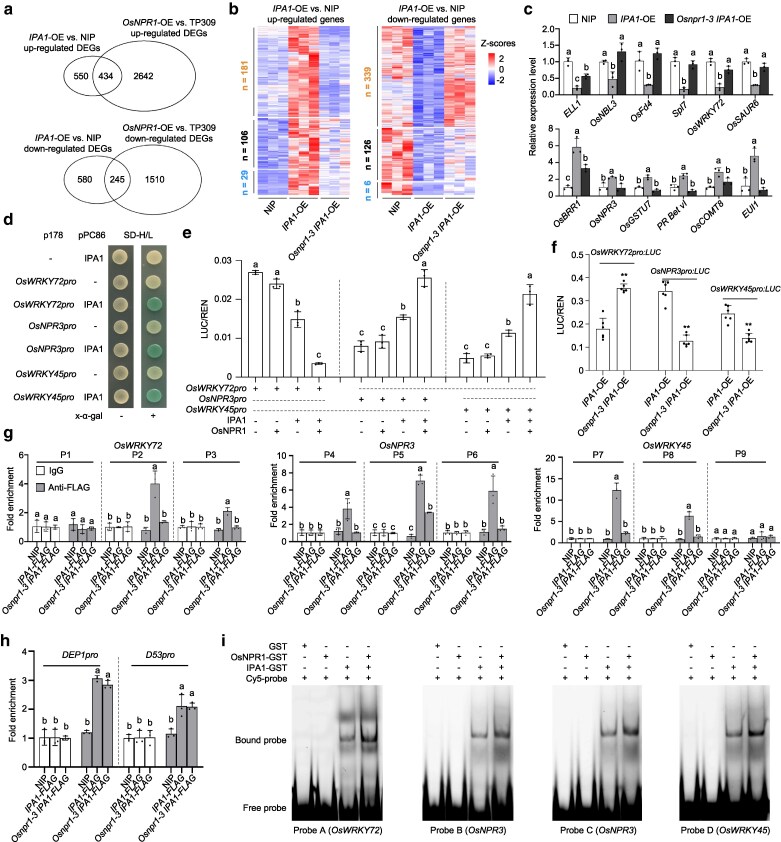
OsNPR1 is required for IPA1 transcriptional activity. a) Venn diagrams showing the extent of overlap between upregulated DEGs in *IPA1*-OE (L5) and upregulated DEGs in *OsNPR1*-OE (G316) and the overlap between downregulated DEGs in *IPA1*-OE (L5) and downregulated DEGs in *OsNPR1*-OE (G316), relative to their corresponding WT. b) Heatmap representation of the relative expression levels of IPA1-upregulated genes (left) or -downregulated genes (right) that depend on OsNPR1 in NIP, *IPA1*-OE, and *Osnpr1-3 IPA1*-OE plants. The color key (blue to red) represents the Z-scores gene expression. Z-scores were calculated using the formula Z = {x−mean (x)}/sd (x), with x represents the standardized counts normalized by DEseq2 package's “counts” function. c) RT-qPCR analysis measuring the relative transcript levels of 6 *IPA1*-upregulated genes (top) and 6 *IPA1-*downregulated genes (bottom) in NIP, *IPA1*-OE, and *Osnpr1*-*3 IPA1*-OE plants. Gene expression levels were examined and normalized to *OsUBQ*. Values are means ± standard deviation (n = 3). d) Y1H assay showing the binding of IPA1 to the promoters of *OsWRKY72*, *OsNPR3* and *OsWRKY45*. e) Dual-luciferase reporter assays showing the effect of OsNPR1 or/and IPA1 on *OsWRKY72*, *OsNPR3*, and *OsWRKY45* transcription in *N. benthamiana* leaves. Values are means ± standard deviation (n = 3). f) Dual-luciferase reporter assays showing the effects of OsNPR1 and/or IPA1 on *OsWRKY72*, *OsNPR3*, and *OsWRKY45* transcription in rice protoplasts from *IPA1*-OE and *Osnpr1-3 IPA1*-OE plants. Values are means ± standard deviation (n = 6). ** indicates *P* < 0.01 by 2-tailed Student *t* test. g) ChIP-qPCR showing the enrichment of *IPA1*-FLAG on different promoter regions of *OsWRKY72*, *OsNPR3*, and *OsWRKY45* in the indicated genotypes. h) ChIP-qPCR showing the enrichment of *IPA1*-FLAG on the promoter regions of *DEP1* and *D53* in the indicated genotypes. i) EMSA showing direct binding of GST–IPA1 to the promoters of *OsWRKY72*, *OsWRKY45*, and *OsNPR3*, and enhanced binding in the presence of OsNPR1. Different lowercase letters (c, e, g and h) indicate significant differences (*P* < 0.05) according to Duncan's new multiple range test. All experiments were repeated at least 3 times.

Using the R package DESeq2, we identified 984 upregulated differentially expressed genes (DEGs) and 825 downregulated DEGs in *IPA1*-OE relative to NIP, and 3,076 upregulated DEGs and 1,755 downregulated DEGs in *OsNPR1*-OE compared with TP309 (log_2_(fold change) ≥ 0.485, false discovery rate [FDR] < 0.05) (Fig. S8b). Among 984 DEGs upregulated in *IPA1*-OE, 434 (44.1%) were also upregulated in *OsNPR1-*OE. Among 825 DEGs downregulated in *IPA1*-OE, 245 (29.7%) were also downregulated in *OsNPR1-*OE (Fig. 4a). The transcriptome analysis indicates that OsNPR1 and IPA1 largely regulate the expression of common sets of genes.

To investigate the degree to which IPA1-regulated genes depend on OsNPR1 function, we conducted pairwise comparisons of the transcriptomes from *IPA1*-OE, *Osnpr1-3 IPA1*-OE, and NIP plants, leading to the identification of 6 groups of DEGs. A detailed analysis showed that among 984 genes upregulated in *IPA1*-OE compared with NIP, 316 (32.1%) were downregulated in *Osnpr1-3 IPA1*-OE relative to *IPA1*-OE (Fig. 4b; Fig. S8c; Supplementary Data Set S3). By contrast, among the 825 genes downregulated in *IPA1*-OE compared with NIP, 471 (57.1%) were upregulated in *Osnpr1-3 IPA1*-OE relative to *IPA1*-OE (Fig. 4b; Fig. S8c; Supplementary Data Set S3). These results demonstrated that OsNPR1 contributes substantially to the expression of IPA1-regulated genes.

Gene Ontology (GO) enrichment term analysis revealed that the IPA1–OsNPR1 upregulated genes are enriched in several biological processes, including “response to stress,” “response to chemical,” “response to abiotic stimulus,” “oxidation-reduction process,” and “defense response.” etc. A similar analysis of IPA1–OsNPR1 downregulated genes revealed an enrichment in biological processes such as “response to stress,” “response to hormone,” “oxidation-reduction process,” “lignin metabolic process,” and “defense response.” etc. (Fig. S8d; Supplementary Data Set S4).

To further test the effect of OsNPR1 on the expression of IPA1-regulated genes, we selected several IPA1–OsNPR1 co-regulated genes for RT-qPCR validation. For IPA1–OsNPR1 downregulated genes, we selected negative regulators of defense responses: *EARLY LESION LEAF 1* (*ELL1*), *NATURAL BLIGHT LEAF 3* (*OsNBL3*), *FERREDOXIN 4* (*OsFd4*), *SPOTTED LEAF 7* (*Spl7*), *OsWRKY72*, and *SMALL AUXIN UP-REGULATED 6* (*OsSAUR6*) (Yamanouchi et al. 2002; Hoang et al. 2019; Hou et al. 2019; Cui et al. 2021; Qiu et al. 2021; Lu et al. 2023). The relative transcript levels of these 6 genes were indeed significantly lower in *IPA1*-OE than in NIP and significantly higher in *Osnpr1-3 IPA1*-OE than in *IPA1*-OE (Fig. 4c). When attacked by *Xoo*, the transcriptional level of *OsWRKY72* was increased in WT. In *IPA1*-OE rice plants, the upregulation of *OsWRKY72* by *Xoo* inoculation was largely repressed (Fig. S9a).

We also selected 6 IPA1–OsNPR1 upregulated genes that enhance disease resistance: *BLAST RESISTANCE-RELATED 1* (*OsBRR1*), *OsNPR3*, *GLUTATHIONE S-TRANSFERASE 7* (*OsGSTU7*), *PATHOGENESIS-RELATED* gene *Bet v1* (*PR Bet v1*), *CAFFEIC ACID O-METHYLTRANSFERASE 8* (*COMT8*), and *ELONGATED UPPERMOST INTERNODE 1* (*EUI1*) (Zhu et al. 2006; Yang et al. 2008; Peng et al. 2009; Liang et al. 2022). RT-qPCR analysis showed that compared with those in NIP, their relative transcript levels are higher in *IPA1*-OE but are significantly reduced in *Osnpr1-3 IPA1*-OE (Fig. 4c). In WT plants, *Xoo* inoculation significantly induced the expression of *OsNPR3*, *OsWRKY45*. In *IPA1*-OE plants, *OsNPR3* and *OsWRKY45* were further upregulated compared with that in NIP both before and after *Xoo* inoculation (Fig. S9a).

The validated DEGs indeed had multiple IPA1-binding motifs, GTAC and TGGGCC/T, within their 2-kb promoters (Fig. S9b). The 1-kb promoter of *OsWRKY72*, one of the IPA1-downregulated genes, contains 4 putative IPA1-binding motifs, GTAC, and TGGGCC/T. Likewise, the promoters of *OsWRKY45* (2,540 bp) and *OsNPR3* (3,000 bp), 2 IPA1-upregulated genes, harbor 16 and 20 putative IPA1-binding motifs, respectively (Fig. S9c). A yeast 1-hybrid (Y1H) assay revealed that IPA1 directly binds to the promoters of *OsWRKY72*, *OsWRKY45*, and *OsNPR3* (Fig. 4d). We also tested the transcriptional activity of IPA1 and/or OsNPR1 toward these promoters driving the firefly *luciferase* (*LUC*) reporter gene in *N. benthamiana* leaves (Fig. S9d). Following co-infiltration of the *35S*:*IPA1* effector construct together with the reporter construct *OsWRKY72pro*:*LUC*, we detected lower relative LUC activity relative to the *OsWRKY72pro*:*LUC* reporter alone, whereas co-infiltration of *35S*:*IPA1* with the reporter *OsWRKY45pro*:*LUC* or *OsNPR3pro*:*LUC* resulted in higher relative LUC activity. Notably, OsNPR1 alone has little effect on the transcriptional output of *OsWRKY72*, *OsNPR3*, and *OsWRKY45* as relative LUC activity was comparable to that obtained with the LUC reporter constructs alone. In the presence of OsNPR1, however, the transcriptional activity of IPA1 toward *OsWRKY72*, *OsNPR3*, and *OsWRKY45* was further enhanced (Fig. 4e; Fig. S9d). The reporter construct *OsWRKY72pro*:*LUC* was separately transformed into rice protoplasts of *IPA1*-OE and *Osnpr1-3 IPA1*-OE. The relative signals of LUC were much lower in *IPA1*-OE protoplasts than in *Osnpr1-3 IPA1*-OE protoplasts, confirming that OsNPR1 enhances the repressive effect of IPA1 on *OsWRKY72*. For the reporter constructs of *OsNPR3* and *OsWRKY45*, the relative LUC signals were much higher in *IPA1*-OE protoplasts than in *Osnpr1-3 IPA1*-OE protoplasts (Fig. 4f), suggesting that OsNPR1 increases the transcriptional activity of IPA1 on *OsNPR3* and *OsWRKY45*.

To investigate the effect of OsNPR1 on the transcriptional activity of IPA1 in rice plants, using its native promoter, we generated *IPA1*:*IPA1-3xFLAG* (*IPA1*-*FLAG*) transgenic rice plants, and crossed the transgenic *IPA1*-*FLAG* line with *Osnpr1-3* to obtain *Osnpr1-3 IPA1-FLAG*. Using these materials, we performed chromatin immunoprecipitation (ChIP)-qPCR (ChIP-qPCR) with an anti-FLAG antibody. Consistent with the above results, IPA1 was found to bind to 2 of the 3 tested regions in the promoters of *OsWRKY45* and *OsWRKY72*, and all 3 tested regions in the promoter of *OsNPR3* in *IPA1-FLAG*. However, the binding ability of IPA1 to these regions was significantly reduced in *Osnpr1-3 IPA1-FLAG* plants (Fig. 4g; Fig. S9c). We next examined 2 developmental genes, *DEP1* and *D53*, which are directly bound and transcriptionally regulated by IPA1 (Lu et al. 2013; Song et al. 2017). The ChIP-qPCR results showed that the enrichment levels in *Osnpr1-3 IPA1-FLAG* are comparable to that in *IPA1-FLAG* (Fig. 4h), suggesting that OsNPR1 is not required for the transcription activity of IPA1 toward these 2 developmental genes. Furthermore, electrophoretic mobility shift assays (EMSA) showed that recombinant IPA1-GST binds to the promoters of *OsWRKY72*, *OsNPR3*, and *OsWRKY45*, and the presence of OsNPR1-GST further enhanced the DNA-binding ability of IPA1 on those DNA fragments (Fig. 4i). These findings demonstrate that the binding of IPA1 with the promoters of *OsWRKY72*, *OsNPR3*, and *OsWRKY45* require the co-transcriptional factor OsNPR1. Furthermore, these results demonstrate that OsNPR1 likely specifically enhances the transcriptional activity of IPA1 on defense genes and can either activate positive regulator genes (*OsWRKY45* and *OsNPR3*) or repress negative regulator gene involved in defense (*OsWRKY72*).

### Downstream genes transcriptionally regulated by IPA1 contribute to IPA1-mediated disease resistance

**Figure 5:**
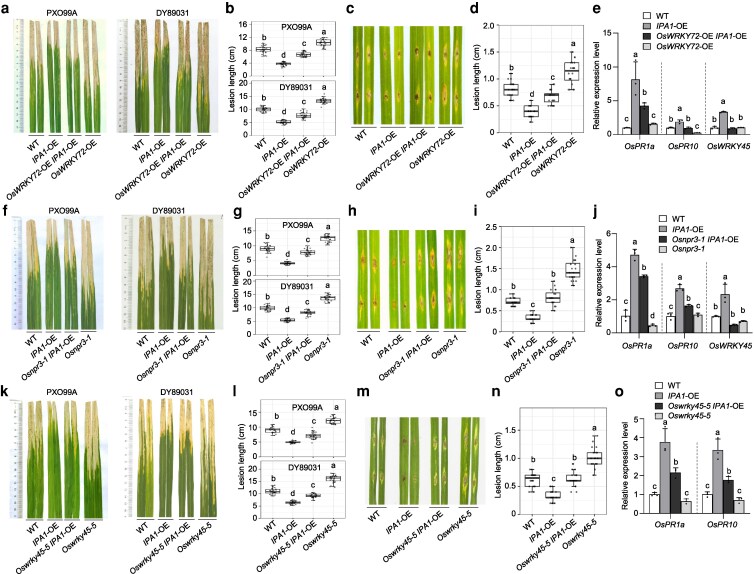
IPA1-regulated genes contribute to IPA1-mediated disease resistance. a, f and k) Lesion symptoms caused by bacterial blight in the indicated genotypes at 14 dpi inoculated with *Xoo* strain PXO99A (left) and DY89031 (right). b, g and l) Lesion length in the indicated genotypes at 14 dpi inoculated with *Xoo* strain PXO99A (top) or DY89031 (bottom) (n = 30). c, h and m) Lesion symptoms in the indicated genotypes at 7 dpi inoculated with *M. oryzae* strain FJ81278 using punch-inoculated method. d, i and n) Lesions length in the indicated genotypes at 7 dpi with strain FJ81278 (n = 20). e and j) Relative transcript levels of *OsPR1a*, *OsPR10*, and *OsWRKY45* in the indicated genotypes. o) Relative transcript levels of *OsPR1a* and *OsPR10* in the indicated genotypes. Gene expression levels were examined and normalized to *OsUBQ*. Values are means ± standard deviation (n = 3). Boxplots in (b), (d), (g), (i), (l), and (n) show median and interquartile range, and error bars denote the full range excluding outliers. Different lowercase letters indicate significant differences (*P* < 0.05) according to Duncan new multiple range test. All experiments were repeated at least 3 times.

To investigate whether IPA1 transcriptionally regulated genes contribute to disease resistance, we conducted a genetic analysis. Accordingly, we crossed 1 *OsWRKY72-*OE line that is more susceptible to *Xoo* than NIP (Hou et al. 2019) into the *IPA1*-OE line L8 to obtain *OsWRKY72*-OE *IPA1*-OE (Fig. S10a). Bacterial blight caused by *Xoo* infection produced significantly longer lesions on the leaves of *OsWRKY72*-OE *IPA1*-OE than those of *IPA1*-OE (Fig. 5, a and b). The lesions caused by *M. oryzae* were also significantly longer in *OsWRKY72*-OE *IPA1*-OE than in *IPA1*-OE (Fig. 5, c and d). The relative transcript levels of *OsPR1a*, *OsPR10*, and *OsWRKY45* were significantly lower in *OsWRKY72*-OE *IPA1*-OE compared with *IPA1*-OE (Fig. 5e). These results demonstrate that the downregulation of *OsWRKY72* by IPA1 lessens disease susceptibility.

We selected a target gene of IPA1, *OsNPR3*, a member of rice NPRs that is most closely related to NPR3 in Arabidopsis (Fig. S10b; Files S1 and S2), to generate the *Osnpr3-1* and *Osnpr3-2* mutants by CRISPR/Cas9-mediated gene editing. *Osnpr3-1* and *Osnpr3-2* harbor a 1-bp insertion and 1-bp deletion, respectively, both resulting in a frameshift mutation and the introduction of a premature stop codon before the ANK domain (Fig. S10c). The lesions caused by bacterial blight (*Xoo* infection) and blast disease (*M. oryzae* infection) were much longer in *Osnpr3* than in NIP (Fig. 5, f to i; Fig. S10, d and e), which is consistent with a previous report that upregulation of *OsNPR3* enhances disease resistance against *Xoo* (Bai et al. 2011). We crossed the *Osnpr3-1* mutant to *IPA1*-OE line L5 to obtain *Osnpr3-1 IPA1*-OE (Fig. S10f). The lesions caused by bacterial blight and blast disease in *Osnpr3-1 IPA1*-OE were significantly longer than those in *IPA1*-OE (Fig. 5, f to i). The relative transcript levels of *OsPR1a*, *OsPR10*, and *OsWRKY45* were significantly lower in *Osnpr3-1 IPA1*-OE than in *IPA1*-OE (Fig. 5j). These results suggest that the upregulation of *OsNPR3* by IPA1 also contributes to IPA1-mediated disease resistance.

*OsWRKY45* was previously shown to be an IPA1 upregulated target gene (Wang et al. 2018; Liu et al. 2019a). In this study, we observed that *OsWRKY72* overexpression, or mutation in either *OsNPR1* or *OsNPR3*, blocks *OsWRKY45* transcript accumulation normally seen upon overexpression of *IPA1*, indicating that the upregulation of *OsWRKY45* is involved in not only IPA1 and OsNPR1 but also *OsWRKY72* and *OsNPR3*. We thus examined the effect of mutated *OsWRKY45* on IPA1-mediated disease resistance. We generated 2 *Oswrky45* mutants via CRISPR/Cas9 editing, *Oswrky45-4* and *Oswrky45-5*. *Oswrky45-5* had a 1-bp insertion, causing a frameshift mutation and resulting in a variant protein retaining only the first 78 aa of OsWRKY45, followed by 250 amino acids not present in WT OsWRKY45 (Fig. S10g). The lesions caused by bacterial blight and blast disease were much longer in *Oswrky45-4* and *Oswrky45-5* than in TP309 (Fig. S10, h and i). We produced a specific antibody against OsWRKY45 using a fragment corresponding to aa 180 to 326 (thus after the frameshift) to examine OsWRKY45 abundance in the *Oswrky45-5* mutant. We detected no signal for OsWRKY45 in the *Oswrky45-5* mutant (Fig. S10j), indicating that *Oswrky45-5* is probably a null allele. We crossed *Oswrky45-5* to *IPA1*-OE line L5 to obtain *Oswrky45 IPA1*-OE. An immunoblot analysis with our specific anti-OsWRKY45 antibody confirmed the greater accumulation of OsWRKY45 in *IPA1*-OE, with a complete loss of signal in *Oswrky45 IPA1*-OE (Fig. S10j). The lesions caused by bacterial blight and blast disease were significantly longer in *Oswrky45 IPA1*-OE than in *IPA1*-OE (Fig. 5, k to n). The relative transcript levels of *OsPR1a* and *OsPR10* were significantly lower in *Oswrky45 IPA1*-OE than in *IPA1*-OE (Fig. 5o). These results indicate that the upregulation of *OsWRKY45* by IPA1 enhances disease resistance. Collectively, our genetic analysis demonstrates that both positive and negative regulator genes in defense, whose transcription is regulated by IPA1, contribute to IPA1-mediated disease resistance.

### IPA1 stabilizes nucleus-localized OsNPR1

During the course of our genetic analysis, we noticed that OsNPR1 protein was more abundant in 2 MIM156 lines and in 2 *IPA1*-OE lines than in WT (Fig. 2a and 3a; Fig. S3c and Fig. S7a). We thus examined OsNPR1 abundance in 3 independent MIM156 lines, confirming the generally higher OsNPR1 protein levels in MIM156 lines compared with ZH11 (Fig. S11a). Importantly, relative *OsNPR1* transcript levels were comparable in MIM156 and ZH11 (Fig. S11b), suggesting that the increase in OsNPR1 protein is a post-transcriptional effect. Because degradation is critical for NPR1 abundance in Arabidopsis and rice (Tada et al. 2008; Spoel et al. 2009; Fu et al. 2012), we hypothesized that OsNPR1 stability is likely altered in MIM156 plants. To test this possibility, we performed a cell-free degradation assay for the endogenous OsNPR1, which demonstrated that OsNPR1 is significantly more stable in MIM156 extracts than in ZH11 extracts (Fig. S11c). Nucleocytoplasmic fractionation and immunoblot analysis of the resulting fractions indicated that the levels of nuclear OsNPR1 are higher in MIM156 than in ZH11. In contrast, the cytosolic pool of OsNPR1 was smaller in MIM156 than in ZH11 (Fig. S11d).

**Figure 6:**
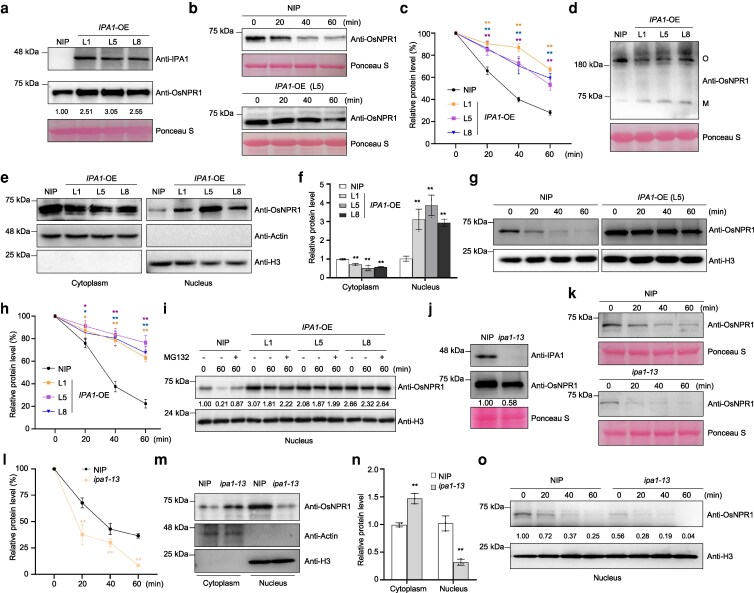
IPA1 promotes OsNPR1 accumulation in the nucleus by repressing its degradation. a) Abundance of OsNPR1 and IPA1 in NIP and 3 *IPA1*-OE lines as indicated by immunoblotting. Ponceau S staining of the Rubisco large subunit served as loading control. The relative abundance of OsNPR1 was quantified using ImageJ, with the levels in NIP set to 1. b) Cell-free degradation assay of OsNPR1 in total protein extracts prepared from NIP (top) or *IPA1*-OE (L5, bottom). Ponceau S staining of the Rubisco large subunit served as loading control. c) Quantification of OsNPR1 abundance at the indicated time points in NIP and 3 *IPA1*-OE lines based on 3 biological replicates. d) Abundance of OsNPR1 oligomers and monomers in NIP and 3 *IPA1*-OE lines, as determined by nonreducing (-DTT) immunoblotting. Abbreviations: M, monomer; O, oligomer. Ponceau S staining of the Rubisco large subunit served as loading control. e) Subcellular fractionation assays showing the abundance of OsNPR1 in the cytoplasmic and nuclear fractions in protein extracts prepared from NIP and 3 *IPA1*-OE lines. Histone H3 and actin served as loading controls for the nuclear and cytosolic fractions, respectively. f) Quantification of relative OsNPR1 abundance in the cytoplasm and nucleus for NIP and 3 *IPA1*-OE lines. g) Cell-free degradation assay for nucleus-localized OsNPR1 in NIP and *IPA1*-OE (L5 line). Histone H3 served as loading control for the nuclear fraction. h) Quantification of relative nucleus-localized OsNPR1 abundance in NIP and 3 *IPA1*-OE lines at the indicated time points. i) Effect of MG132 treatment on the abundance of nucleus-localized OsNPR1 in NIP and 3 *IPA1*-OE lines at the indicated time points. Histone H3 served as loading control for the nuclear fraction. j) Abundance of OsNPR1 and IPA1 in *ipa1-13* and NIP as indicated by immunoblotting. Ponceau S staining of the Rubisco large subunit served as loading control. k) Cell-free degradation assay of OsNPR1 in total protein extracts prepared from NIP (top) or *ipa1-13* (bottom). Ponceau S staining of the Rubisco large subunit served as loading control. l) Quantification of OsNPR1 abundance at the indicated time points in *ipa1-13* and NIP based on 3 biological replicates. m) Subcellular fractionation assays showing the abundance of OsNPR1 in the cytoplasmic and nuclear fractions in protein extracts prepared from *ipa1-13* and NIP. Histone H3 and actin served as loading controls for the nuclear and cytosolic fractions, respectively. n) Quantification of relative OsNPR1 abundance in the cytoplasm and nucleus for *ipa1-13* and NIP. o) Cell-free degradation assay for nucleus-localized OsNPR1 in *ipa1-13* and NIP. Histone H3 served as loading control for the nuclear fraction. In (c), (f), (h), (l) and (n), values are means ± standard deviation; * indicates significant differences *P* < 0.05; ** indicates *P* < 0.01 by 2-tailed Student *t* test. All experiments were repeated at least 3 times.

When we analyzed OsNPR1 abundance in *IPA1*-OE lines, we observed increased OsNPR1 accumulation in all 3 *IPA1*-OE lines tested (Fig. 6a). Notably, RNA-seq showed that the transcriptional levels of *OsNPR1* in *IPA1*-OE and NIP are comparable (Fig. S12a), and RT-qPCR analysis further confirmed that the relative transcript levels of *OsNPR1* were similar across all genotypes (Fig. S12b), suggesting that the observed effects are primarily regulated at the protein level rather than the transcriptional level.

Given that miR156 negatively and IPA1 positively regulated OsNPR1 protein levels, we asked whether OsNPR1 regulated IPA1 expression levels. In 3 *OsNPR1*-*FLAG* lines, the transgenic *OsNPR1* was driven by its native promoter, and the mRNA levels of *OsNPR1* were increased by 4- to 17-fold than the WT, while the mRNA levels of *IPA1* were comparable to that of WT. In G316 (*OsNPR1*-OE in the TP309 background, *OsNPR1* being driven by the CaMV 35S promoter), the mRNA levels of *OsNPR1* were more than 200 times that of WT, and the mRNA levels of *IPA1* were more than 3 times that of WT (Fig. S12, c and d). Consistent with mRNA levels, the protein levels of OsNPR1 in *OsNPR1*-*FLAG* were 3 to 6 times of the WT, while in G316, the protein level of OsNPR1 was more than 20 times that of TP309. The protein levels of IPA1 in *OsNPR1*-*FLAG* plants were comparable to that in WT. In G316, however, the protein level of IPA1 was almost 3 times that of TP309 (Fig. S11e). These results suggested that the expression levels and expression pattern of OsNPR1 possibly affect IPA1 expressions.

The plant height and tiller number of *OsNPR1*-*FLAG* transgenic plants were comparable to the counterparts of NIP WT. In the *OsNPR1*-OE (G316 line), the plant height and tiller number were decreased than that in TP309 (Fig. S12, f and g). The morphological phenotypic difference between *OsNPR1*-*FLAG* and *OsNPR1*-OE were likely partially due to IPA1 abundance. The disease resistance of *OsNPR1*-FLAG and *OsNPR1*-OE plants against *Xoo* was positively correlated to OsNPR1 expression levels (Fig. S12, h and i), consistent with our previous report (Li et al. 2016).

A cell-free degradation assay revealed that OsNPR1 is more stable in protein extracts prepared from each of the 3 *IPA1*-OE lines than in those prepared from NIP (Fig. 6, b and c). The addition of MG132, an inhibitor of the 26S proteasome, largely blocked the degradation of OsNPR1 in WT, and to some extent in *IPA1*-OE (Fig. S13a). These results suggest that IPA1 increases OsNPR1 protein abundance, possibly through enhancing its stability in relation to the ubiquitin-mediated degradation pathway.

Previous reports have shown that NPR1 undergoes a transition from oligomers to monomers in the cytoplasm upon SA treatment, after which OsNPR1 monomers translocate to the nucleus and activate the expression of defense-related genes in Arabidopsis (Mou et al. 2003; Tada et al. 2008). In rice, OsNPR1 also relocates to the nucleus upon a change in the cell redox status (Yuan et al. 2007). Under nonreducing conditions, we isolated total proteins and examined OsNPR1 abundance. Monomeric OsNPR1 accumulated to higher levels in *IPA1*-OE than in NIP, while oligomeric OsNPR1 was less abundant in *IPA1*-OE than in NIP (Fig. 6d). We further examined the distribution of OsNPR1 oligomers and monomers following infection with *Xoo* or *M. oryzae*. Interestingly, pathogen treatment led to an overall increase in total OsNPR1 protein levels (Fig. 1e), resulting in the accumulation of both oligomeric and monomeric forms in NIP (Fig. S13b). Nucleocytoplasmic fractionation analysis indicated that *IPA1*-OE lines accumulate higher levels of nuclear OsNPR1 than in NIP, concomitant with a lower abundance of cytosolic OsNPR1 in *IPA1*-OE than in NIP (Fig. 6, e and f). To confirm the effect of IPA1 on nucleocytoplasmic distribution of OsNPR1, we generated *OsNPR1*-*GFP* transgenic rice plants. We then crossed *OsNPR1*-*GFP* with *IPA1*-OE (L5) to get *IPA1*-OE *OsNPR1*-*GFP* rice plants. The OsNPR1-GFP localized both in cytoplasm and nucleus by confocal scanning the protoplast isolated from *OsNPR1*-GFP and *IPA1*-OE *OsNPR1*-GFP. The quantification results showed that OsNPR1-GFP accumulated much more in nucleus of *IPA1*-OE *OsNPR1*-*GFP* than in nucleus of *OsNPR1*-*GFP* (Fig. S13c). The fluorescence signals were much stronger in nucleus of *IPA1*-OE *OsNPR1*-*GFP* than in nucleus of *OsNPR1*-*GFP* by directly scanning the seedling (Fig. S13d). The immunoblotting confirmed that the protein levels of OsNPR1-GFP were much higher in nucleus of *IPA1*-OE *OsNPR1*-*GFP* than in nucleus of *OsNPR1*-*GFP* (Fig. S13e). Thus, the nucleocytoplasmic distribution of OsNPR1 was consistent with the fraction of OsNPR1 present as oligomer or monomer in *IPA1*-OE and WT.

The stability of cytoplasmic OsNPR1 in *IPA1*-OE was comparable to that in WT (Fig. S13, f and g). In addition, MG132 treatment had little effect on cytoplasmic accumulation of OsNPR1 in *IPA1*-OE and WT (Fig. S13h). In the nucleus fractions, however, OsNPR1 was more stable in *IPA1*-OE than in WT (Fig. 6, g and h), and MG132 treatment substantially inhibited OsNPR1 degradation in WT and *IPA1*-OE (Fig. 6i). Thus, IPA1 stabilizes nucleus-localized OsNPR1, likely by interfering with the 26S proteasome degradation pathway.

To confirm the effect of IPA1 on OsNPR1 protein stability, we examined the OsNPR1 protein levels in *ipa1-13* and *IPA1*-RNAi, which abolished and substantially reduced IPA1 protein accumulation, respectively, and found that the abundance of OsNPR1 was significantly reduced in *ipa1-13* and *IPA1*-RNAi than in WT (Fig. 6j; Fig. S14, b and d). The mRNA levels of *OsNPR1*, however, were comparable in *ipa1-13* and *IPA1*-RNAi than in WT (Fig. S14, a and c). OsNPR1 exhibited much less stable in *ipa1-13* and *IPA1*-RNAi than in WT by the degradation assay (Fig. 6, k and l; Fig. S14e). The levels of nuclear- and cytoplasm-localized OsNPR1 were much lower and higher in *ipa1-13* than in WT, respectively (Fig. 6, m and n). In addition, the stability of nucleus-localized OsNPR1 was much lower in *ipa1-13* than in WT (Fig. 6o). These results demonstrate that the abolishment of IPA1 makes nucleus-localized OsNPR1 less stable.

### IPA1 represses OsNPR1 degradation by interfering with OsCUL3a

In Arabidopsis, CULLIN3 (CUL3) functions as an E3 ligase for NPR1 turnover (Spoel et al. 2009; Fu et al. 2012). In rice, OsNPR1 is also targeted by OsCUL3a for degradation (Liu et al. 2017). In a LUC complementation imaging assay in *N. benthamiana* leaves and a Y2H assay, we confirmed the interaction between OsNPR1 and OsCUL3a (Fig. S15, a and b). Notably, the 2 domains of N terminus OsCUL3a, Cullin-repeat (aa 1 to 377) and Cullin-homology (aa 378 to 649), but not the C terminus (aa 650 to 736) each weakly interacted with full-length OsNPR1 in yeast, while the N terminus of OsCUL3a, containing both Cullin-repeat and Cullin-homology domains (aa 1 to 649), interacted more strongly with full-length OsNPR1 (Fig. S15, c and d). The N terminus of OsNPR1 that contains the BTB domain (aa 1 to 268), but not its C terminus with the ANK domain (aa 269 to 582), interacted with full-length OsCUL3a (Fig. S15d). We further determined that the N terminus of OsNPR1 (aa 1 to 268) only interacts with the N terminus of OsCUL3a containing both Cullin-repeat and Cullin-homology domains of OsCUL3a (aa 1-649) (Fig. S15e).

**Figure 7:**
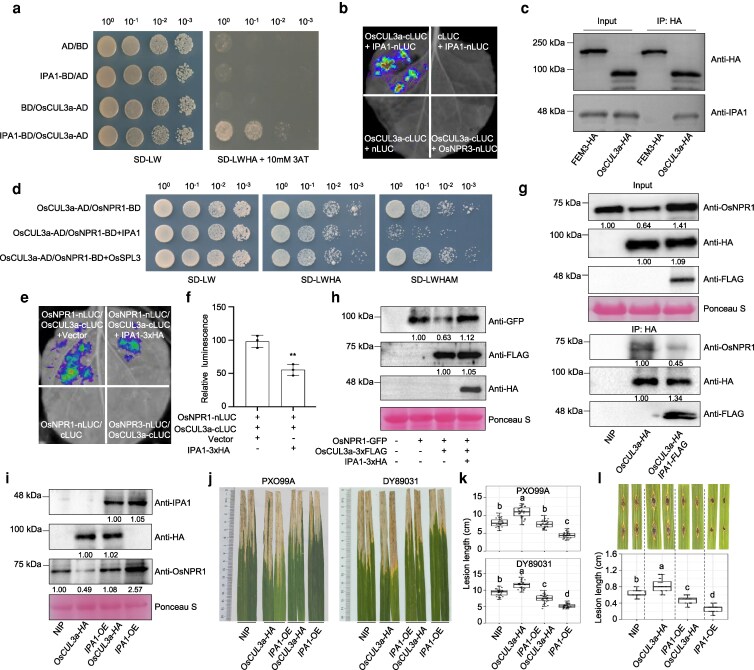
IPA1 stabilizes OsNPR1 by interfering with OsCUL3a. a) Yeast 2-hybrid assay showing that IPA1 interacts with OsCUL3a. b) LUC complementation assay showing the IPA1-OsCUL3a interaction in *N. benthamiana* leaves, OsNPR3-OsCUL3a was used as a negative control. c) Co-IP assay showing that IPA1 co-precipitates with OsCUL3a in transgenic plants of *OsCUL3apro*:*OsCUL3a*-*3xHA* following immunoprecipitation with an anti-HA antibody, *FEM3*-HA served as negative control. d) Y3H assay testing the effect of IPA1 and OsSPL3 on the interaction between OsNPR1 and OsCUL3a. M, methionine. e) LUC complementation assay between OsCUL3a and OsNPR1 in the absence or presence of IPA1 in *N. benthamiana* leaves. f) Quantification of relative LUC activity derived from the reconstitution of LUC by the interaction of OsNPR1 and OsCUL3a in the presence or absence of IPA1. Values are means ± standard deviation (n = 3). ** indicates *P* < 0.01 by 2-tailed Student *t* test. g) Co-IP assay with an anti-HA antibody using *OsCUL3a-HA* and *OsCUL3a-HA IPA1*-*FLAG* transgenic rice plants. h) OsCUL3a induces OsNPR1 degradation in *N. benthamiana* leaves, which is counteracted by IPA1. *OsNPR1-GFP* and *OsCUL3a-3xFLAG* were co-infiltrated in *N. benthamiana* leaves alone or together with *IPA1-3xHA*. Ponceau S staining of the Rubisco large subunit served as loading control. i) The protein levels of IPA1, OsCUL3a-HA, and OsNPR1 were examined by immunoblotting in the indicated genotypes. j) Lesion symptoms caused by bacterial blight in the indicated genotypes at 14 dpi with *Xoo* strain PXO99A (left) and DY89031 (right). k) Length of lesions in the indicated genotypes at 14 dpi with *Xoo* strain PXO99A or DY89031 (n = 30). l) Blast disease symptoms (up) and lesion length (n = 20, bottom) at 7 dpi after inoculation with *M. oryzae* strain FJ81278 using the punch-inoculated method. In (k) and (l), boxplots show median and interquartile range, and the error bars denote the full range excluding outliers. Different lowercase letters indicate significant differences (*P* < 0.05) according to Duncan new multiple range test. All experiments were repeated at least 3 times.

As OsNPR1 interacts with both IPA1 and OsCUL3a, it is possible that IPA1 may physically interact with OsCUL3a. To test this hypothesis, we performed a Y2H assay and LUC complementation imaging assay in *N. benthamiana* leaves. Indeed, IPA1 interacted with OsCUL3a (Fig. 7, a and b). We produced transgenic *OsCUL3apro*:*OsCUL3a*-*3xHA* rice plants (*OsCUL3a*-*HA*) and used them for a co-IP assay with anti-HA antibody. We detected IPA1 in the immunoprecipitated proteins of *OsCUL3a*-*HA* but not in immunoprecipitated proteins of *FEM3-HA* (*FEM3*, *Five Elements Mountain 3*, encoding the largest subunit of rice RNA polymerase V) (Zheng et al. 2021) (Fig. 7c), suggesting that IPA1 and OsCUL3a interact in vivo.

Since IPA1 interacts with OsCUL3a, which serves as an E3 ligase, it is possible that IPA1 degradation is mediated by OsCUL3a. To address this possibility, we used 3 lines of *OsCUL3a*-*HA* and created 2 *Oscul3a* mutants by CRISPR/Cas9 technology (Fig. S16, a and b). In 3 lines of *OsCUL3a-HA* plants, as expected, OsNPR1 protein levels were significantly reduced compared with WT. In contrast, the IPA1 protein levels in *OsCUL3a-HA* plants were comparable to that in WT. As previously reported (Liu et al. 2017), OsNPR1 protein levels were accumulated higher in the 2 *Oscul3a* mutants than in WT. The IPA1 protein levels in the 2 *Oscul3a* mutants, however, were the same as WT (Fig. S16c). These results exclude the possibility that OsCUL3a mediates degradation of IPA1.

To elucidate the protein-protein interaction relationship among IPA1, OsNPR1, and OsCUL3a, we turned to a yeast 3-hybrid (Y3H) assay. When IPA1 was expressed, the growth of yeast cells harboring constructs encoding OsCUL3a-AD and OsNPR1-BD declined. When OsSPL3 that does not interact with OsNPR1 (Fig. S2a) was expressed, the growth of yeast cells with OsCUL3a-AD and OsNPR1-BD did not change (Fig. 7d; Fig. S17). In a LUC complementation imaging assay, the relative LUC activity when the constructs *OsNPR1-nLUC* and *OsCUL3a-cLUC* were co-infiltrated in *N. benthamiana* leaves decreased significantly in the presence of *IPA1* than in the absence of *IPA1* (Fig. 7, e and f).

When we expressed *OsCUL3a*-*HA* in rice plants, the protein level of OsNPR1 was decreased accordingly (Fig. S16c). When the transgenic *IPA1*-*FLAG* was introduced into *OsCUL3a*-*HA*, the OsNPR1 protein level was rescued (Fig. 7g). These genetic experiments suggest that OsCUL3a and IPA1 function antagonistically on OsNPR1 abundance. In a co-IP assay with an anti-HA antibody, we co-immunoprecipitated much less OsNPR1 from *OsCUL3a*-*HA IPA1*-*FLAG* extracts than from *OsCUL3a*-*HA* (Fig. 7g). The co-infiltration of *N. benthamiana* leaves with *OsNPR1-GFP* and *OsCUL3a-3xFLAG* resulted in lower OsNPR1-GFP protein levels than when *OsNPR1-GFP* is expressed alone. The additional co-infiltration of *IPA1*-*3xHA* counteracted the negative effect of OsCUL3a-3xFLAG on OsNPR1-GFP abundance (Fig. 7h). These experiments demonstrate that IPA1 interferes with the interaction between OsCUL3a and OsNPR1 and thus stabilizes OsNPR1.

The resistance against bacterial blight and blast disease was compromised in *OsCUL3a*-*HA* relative to NIP probably due to reduced OsNPR1 protein levels (Fig. 7, i to l). Introducing the *IPA1*-OE transgene into the *OsCUL3a*-*HA* line (yielding *IPA1*-OE *OsCUL3a*-*HA*) led to increased OsNPR1 levels and enhanced disease resistance than *OsCUL3a*-*HA* (Fig. 7, i to l). These results suggest that OsCUL3a and IPA1 have opposite effects on disease resistance mainly through regulating the protein levels of OsNPR1.

miR156-OE rice plants had lower levels of IPA1 and possibly its homologs and accumulated less OsNPR1 accordingly (Fig. S18a), although relative *OsNPR1* transcript levels showed no significant change in miR156-OE compared with ZH11 (Fig. S18b). A subcellular fractionation assay indicated lower levels of OsNPR1 in both the nucleus and cytoplasm in miR156-OE compared with WT, probably due to the decreased stability of OsNPR1 (Fig. S18, c and d).

We crossed the miR156-OE line with transgenic *OsNPR1-FLAG* plants (Fig. S19a). An immunoblot analysis revealed lower abundance of OsNPR1-FLAG (using anti-FLAG antibody) and total OsNPR1 (using anti-OsNPR1 antibody) in miR156-OE *OsNPR1*-*FLAG* compared with *OsNPR1*-*FLAG* (Fig. S19b), without a corresponding change in *OsNPR1* transcript levels (Fig. S19c). The overaccumulation of miR156 in *OsNPR1*-*FLAG* decreased the stability of OsNPR1 (Fig. S19, d and e). Subcellular fractionation and cell-free degradation assays indicated that the low stability of OsNPR1 in the nucleus, but not in the cytoplasm, is likely the main reason for the decreased OsNPR1 levels observed in miR156-OE *OsNPR1*-*FLAG* compared with *OsNPR1*-*FLAG* (Fig. S19, f to k).

The miR156-OE plants were more susceptible to bacterial blight and blast disease compared with WT. The introduction of miR156-OE into *OsNPR1*-*FLAG*, which exhibits greatly enhanced disease resistance, substantially diminished resistance in miR156-OE *OsNPR1*-*FLAG* (Fig. S20, a to e). *OsNPR1*-*FLAG* showed significantly elevated transcript levels of *OsPR1a* and *OsWRKY45* compared with WT. Consistent with the above disease phenotypes, *OsPR1a* and *OsWRKY45* transcript levels were much lower in miR156-OE *OsNPR1*-*FLAG* than in *OsNPR1*-*FLAG* (Fig. S20f). Together, these results suggested that miR156 diminished disease resistance via modulating OsNPR1 abundance.

## Discussion

We previously demonstrated that IPA1 increased disease resistance against bacterial blight through repressing gibberellic acid (GA) signaling (Liu et al. 2019a). In addition, the GA insensitivity of *IPA1* overexpression rice lines results in enhanced seed dormancy and delayed growth (Liu et al. 2019a; Miao et al. 2019). Genetic analysis showed that activation of GA signaling only partially compromised IPA1-mediated disease resistance (Liu et al. 2019a), indicating the involvement of other signaling pathways in IPA1-mediated disease resistance. Here, we showed that overexpression of *IPA1* or knockdown of miR156 abundance caused ROS accumulation and cell death. Overexpression of *OsNPR1* greatly increased resistance to *Xoo* and resulted in ROS accumulation and spontaneous cell death (Chern et al. 2005; Yuan et al. 2007). The similar autoimmunity phenotypes of *IPA1*-OE and *OsNPR1*-OE plants led us to hypothesize that IPA1 and OsNPR1 function in the same pathway. Here, we provided several lines of evidence that support IPA1 and OsNPR1 physical and genetic interaction in controlling disease resistance. Whether and how GA and SA integrate within IPA1-mediated disease resistance is worth further investigation. Our results discovered that IPA1 and other homologous OsSPLs function in SA signaling. Given that other transcription factors, like OsWRKY45 and several TGAs work in SA signaling (Chern et al. 2001; Shimono et al. 2007), the specificity of these transcription factors in SA signaling is another open question.

While we found that the IPA1-OsNPR1 interaction happens in the nucleus, the possibility that transient interaction of IPA1 with OsNPR1 in the cytoplasm cannot be totally excluded. If this possibility is also true, when IPA1 is translocated into the nucleus, OsNPR1 could be carried into the nucleus as well.

Our results discovered that the protein level of OsNPR1 was increased because of the stabilization effect of IPA1 by interference with the OsCUL3a-OsNPR1 interaction. We previously showed that the abundance of SLR1, another co-transcription factor of IPA1, is higher in *IPA1*-OE (Liu et al. 2019a), suggesting that IPA1 might interfere with the degradation of SLR1 as well. Thus, interaction between a transcription factor and its co-transcription factor, resulting in the stabilization of the co-transcription factor, might be a common phenomenon (Mahendrawada et al. 2025).

IPA1 regulates rice development such as tillering, panicle branching and culm development (Jiao et al. 2010; Miura et al. 2010; Zhang et al. 2017). IPA1 also increases the ability against diseases and multiple abiotic stresses such as drought, salinity, and cold (Wang et al. 2018; Liu et al. 2019a; Jia et al. 2022a, 2022b; Zhu et al. 2022; Chen et al. 2023). How a single transcription factor confers on rice several distinct biological functions is elusive. Our study revealed that the co-transcription factor, OsNPR1 interacts with IPA1 and is required for IPA1-mediated disease resistance but not for growth, tillering and panicle branching. Given that 2 other co-transcription factors, SLR1 and Dwarf 53 (D53), also interact with IPA1 (Song et al. 2017; Liu et al. 2019a), we speculate that IPA1 possesses several distinct functions in development and in responses to various biotic and abiotic stresses due to it interacting with different co-transcription factors in specific spatiotemporal pattern. The identities and characterization of these IPA1 co-transcription factors, which specifically function in different biological responses, are areas for future research.

The transcriptome analysis and transcriptional activity assays showed that IPA1 both up- and downregulates large sets of downstream genes. In addition, OsNPR1 enhanced both the activation and repression activity for transcription. The biochemical mechanism underlying the opposite roles of IPA1-OsNPR1 in transcription likely involves other transcription factors and/or epigenetic factors like histone modifications. The molecular mechanism underlying the opposite role of single transcription factor in transcription regulation warrants further investigation.

We discovered that OsNPR1 interacts with IPA1 via its BTB domain and determined that the OsNPR1 BTB domain also mediates the interaction of OsNPR1 with OsCUL3a. The interactions of IPA1-OsNPR1 and IPA1-OsCUL3a interfere with OsCUL3a-OsNPR1 interaction. Finally, we showed that IPA1, a transcription factor, protects its co-transcription factor, OsNPR1, from degradation by interfering with OsCUL3a, a scaffold protein for various CRL3-type ubiquitin E3 ligase of OsNPR1 (Fig. S21). A recent crystal structural study showed that NPR1 interacts with TGA3 through its ankyrin-repeat domain (Kumar et al. 2022). It is plausible that OsNPR1 interacts with various TFs via different domains. Moreover, OsDWD1-OsCUL4 also plays a critical role in OsNPR1 ubiquitination and degradation (Choi et al. 2022). Whether IPA1 also interferes with OsDWD1-OsCUL4 requires further investigation.

Recently, 2 reports showed that IPA1 can be phosphorylated at serine S163 and ubiquitinated at lysine K29. The phosphorylated IPA1 enhances binding to the promoter of defense gene, while the K29-polyubiquitination does not affect IPA1 stability but enhances its transcriptional activity on *OsWRKY45* (Wang et al. 2018; Shi et al. 2024). Given that OsNPR1 promotes the binding of IPA1 on OsWRKY45 promoter, it is plausible that post-translation modification on IPA1 increases its interaction with OsNPR1 to enhance the transcriptional activity. Another possibility SA regulating the modifications of IPA1 would be worthy investigating.

 

### Accession numbers

*IPA1* (LOC_Os08g39890), *OsNPR1* (LOC_Os01g09800), *OsNPR3* (LOC_Os03g46440), *OsWRKY45* (LOC_Os05g25770), *OsWRKY72* (LOC_Os11g29870), *OsCUL3a* (LOC_Os02g51180).

## Supplementary Material

koag122_Supplementary_Data

## Data Availability

RNA sequencing data have been deposited in the CNGBdb (https://cngb.org/, CNP0005515).

## Dive Curated Terms

The following phenotypic, genotypic, and functional terms are of significance to the work described in this paper:

NPR3 Gramene: AT5G45110
NPR3 Araport: AT5G45110

NPR4 Gramene: AT4G19660
NPR4 Araport: AT4G19660

SPL Gramene: AT4G27330
SPL Araport: AT4G27330

SPL7 Gramene: At5g18830
SPL7 Araport: At5g18830

ANK Gramene: XM_008388629.2
ANK Araport: XM_008388629.2

TGA3 Gramene: AT1G22070
TGA3 Araport: AT1G22070

SLR1 Gramene: LOC_Os03g49990
SLR1 Araport: LOC_Os03g49990
