# Supplementary material for: Mutual potentiation of IPA1 and OsNPR1 enhances rice immunity
Source: Plant Cell. 2026 May 20;38(5):koag122. doi: 10.1093/plcell/koag122 (PMC13188844; doi:10.1093/plcell/koag122)
Supplement: koag122_Supplementary_Data [file koag122_supplementary_data.zip › Supplementary Figures.pdf]

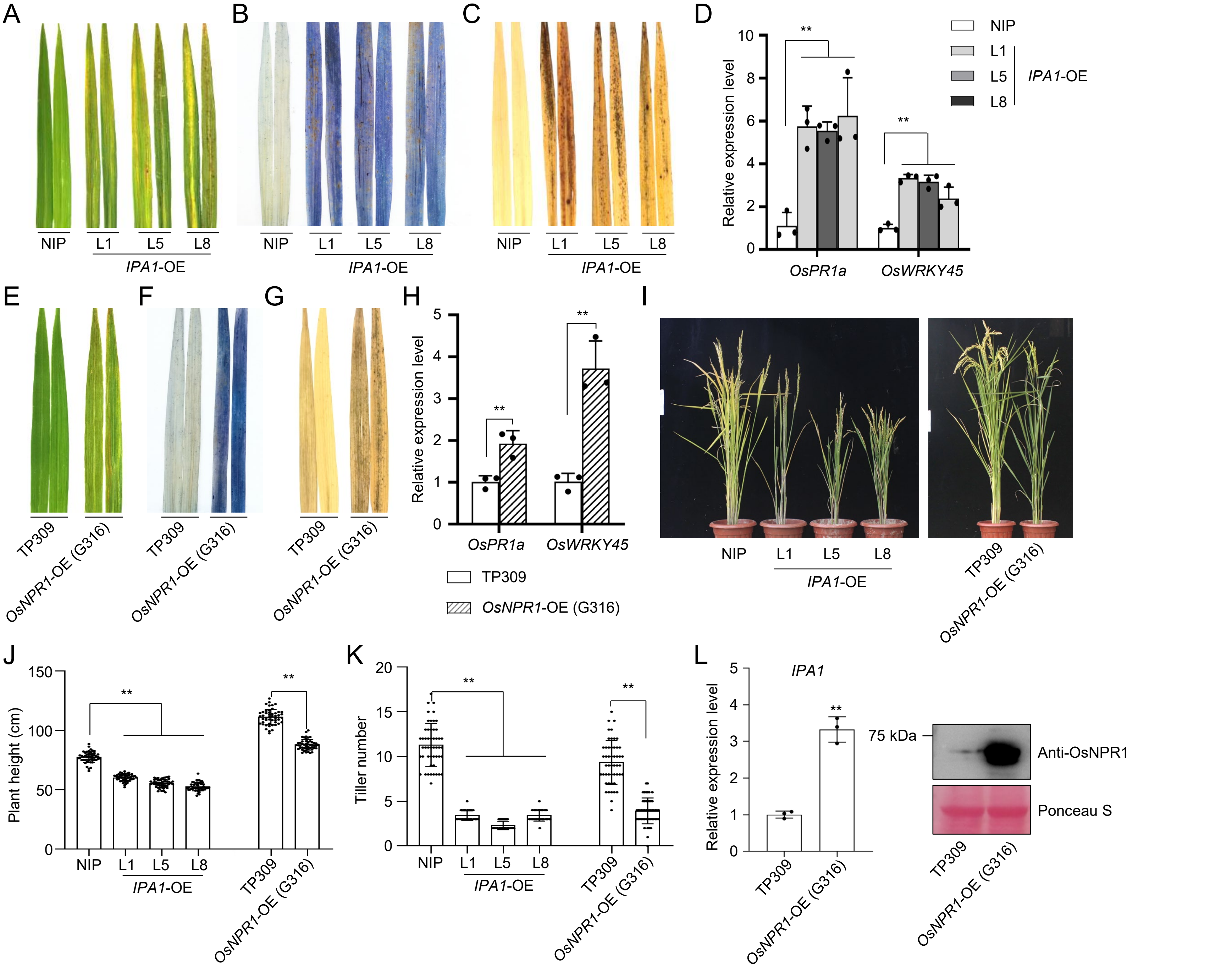

Supplementary Figure S1. *IPA1*-OE and *OsNPR1*-OE rice plants exhibit a lesion-mimic phenotype. (Supports Figure 1)

(*A*) Lesion-mimic phenotypes in three *IPA1*-OE lines at the flowering stage. Wild-type Nipponbare (NIP) at the same developmental stage was used as a control. (*B*) Detection of cell death in NIP and three *IPA1*-OE lines by trypan blue staining. (*C*) Detection of reactive oxygen species (ROS) accumulation in NIP and three *IPA1*-OE lines by DAB staining. (*D*) Relative transcript levels of *OsPR1a* and *OsWRKY45* in NIP and three *IPA1*-OE lines. (*E*) Lesion-mimic phenotypes of *OsNPR1*-OE (G316) plants at the flowering stage. Wild type (TP309) at the same stage served as control. (*F*) Detection of cell death in *OsNPR1*-OE and TP309 by trypan blue staining. (*G*) Detection of ROS accumulation in *OsNPR1*-OE and TP309 by DAB staining. (*H*) Relative transcript levels of *OsPR1a* and *OsWRKY45* in *OsNPR1*-OE and TP309. (*I*) Photograph of 110-day-old rice plants of the indicated genotypes. (*J*) Plant height of 110-day-old rice plants of the indicated genotypes (n = 50). (*K*) Tiller number per plant of the indicated genotypes (n = 50). (*L*) Relative transcript levels of *IPA1* in *OsNPR1*-OE and TP309 (left). The protein levels of OsNPR1 by immunoblotting in *OsNPR1*-OE and TP309 (right). In (*D*), (*H*) and (*L*), gene expression levels were examined and normalized to *OsUBQ*, values are means ± standard deviation (n = 3). ** indicates significant differences ($P < 0.01$) by two-tailed Student's t-test. All experiments were repeated at least three times.

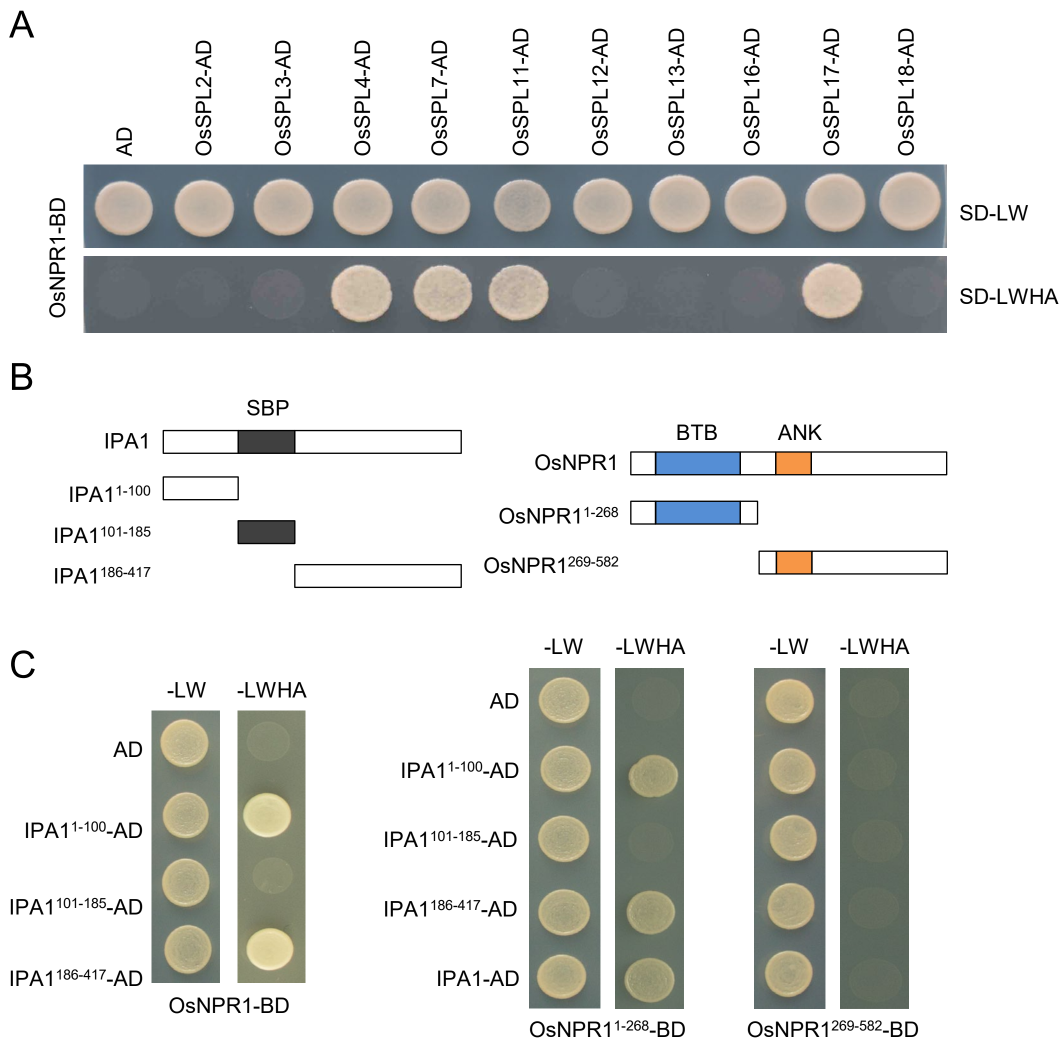

Supplementary Figure S2. Y2H assay between OsNPR1 with IPA1 domains and with other ten OsSPLs. (Supports Figure 1)

(*A*) Y2H assay testing the interaction between OsNPR1 and OsSPL2, OsSPL3, OsSPL4, OsSPL7, OsSPL11, OsSPL12, OsSPL13, OsSPL16, OsSPL17, and OsSPL18. (*B*) Diagrams of the full-length and truncated *IPA1* and *OsNPR1* constructs used in the Y2H assay. (*C*) Y2H assay between three segments IPA1 with full-length OsNPR1 (left), Y2H assay between truncated IPA1 with N-terminal (middle) and C-terminal of OsNPR1 (right). AD, GAL4 activation domain; BD, GAL4 DNA-binding domain; SD, synthetic defined medium; L, leucine; W, tryptophan; H, histidine; A, adenine. All experiments were repeated at least three times.

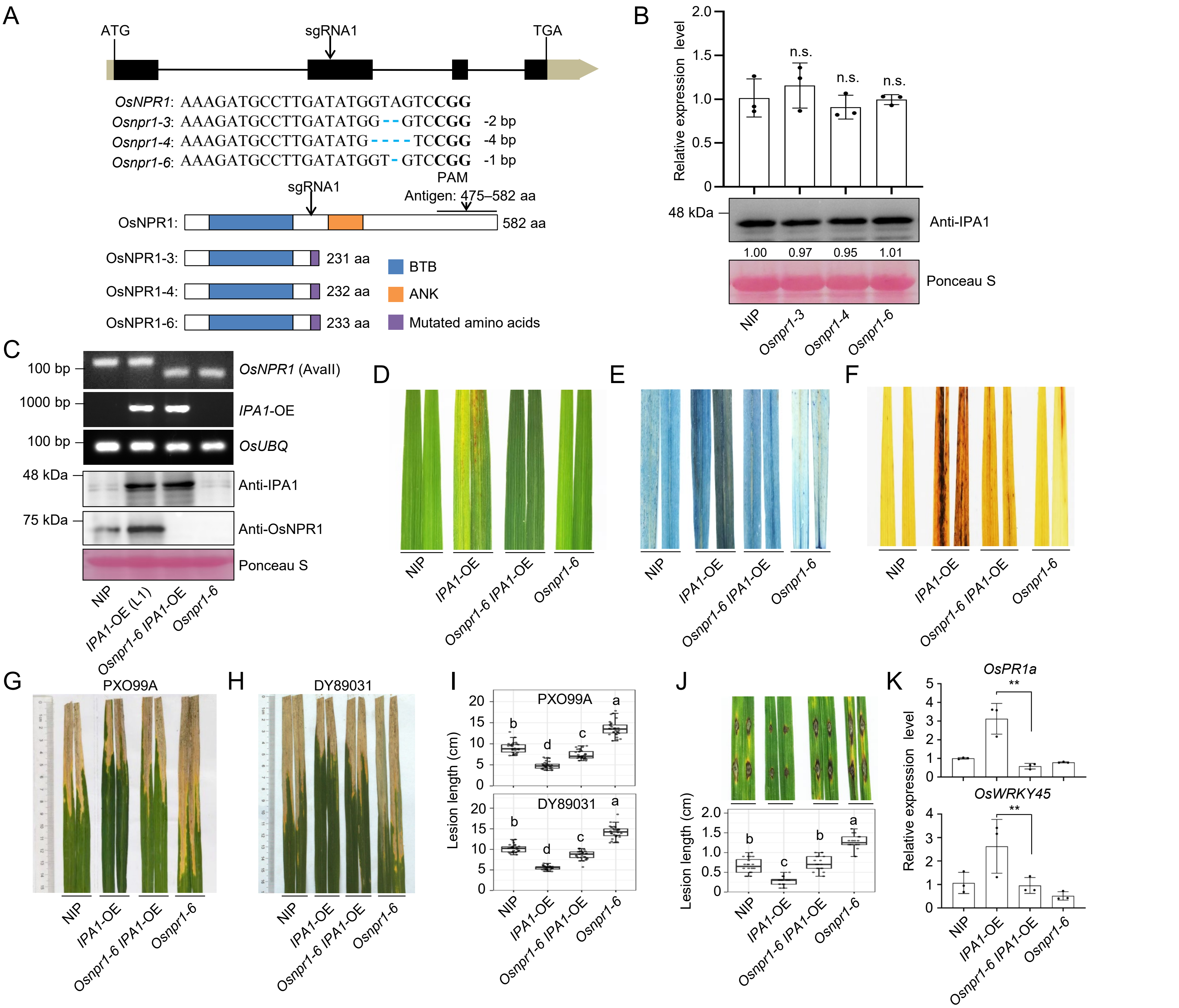

Supplementary Figure S3. IPA1-mediated resistance partially depends on OsNPR1. (Supports Figure 2)

(*A*) Diagram of the *OsNPR1* locus with the sgRNA target site for genome editing. The mutations in *OsNPR1* are shown in blue. PAM, protospacer adjacent motif. Diagram of wild-type OsNPR1 and the predicted OsNPR1 mutant proteins in the *Osnpr1* mutants. (*B*) The relative transcript levels of *IPA1* by real-time PCR in *Osnpr1* mutants and NIP (top). Gene expression levels were examined and normalized to *OsUBQ*. Values are means ± standard deviation (n = 3). The protein levels of IPA1 by immunoblotting in *Osnpr1* mutants and NIP (bottom). (*C*) Genotyping PCR of *Osnpr1-6* by dCAPS marker and *IPA1*-OE in the indicated genotypes. *OsUBQ* served as control (top). Immunoblot analysis of OsNPR1 and IPA1 protein levels in the indicated genotypes (bottom); Ponceau S staining of the large subunit of Rubisco served as loading control. (*D*) Lesion-mimic phenotypes in the indicated genotypes. (*E*) Cell death in the indicated genotypes based on trypan blue staining. (*F*) ROS accumulation in the indicated genotypes as shown by DAB staining. (*G*) and (*H*) Lesion symptoms caused by bacterial blight in the indicated genotypes at 14 days post inoculation (dpi) with *Xoo* strain PXO99A (*G*) and DY89031 (*H*). (*I*) Length of lesions in the indicated genotypes at 14 dpi with *Xoo* strain PXO99A or DY89031 (n = 30). (*J*) Blast disease symptoms and length of lesions (n = 20) in the indicated genotypes at 7 dpi after inoculation with *M. oryzae* strain FJ81278 using the punch-inoculated method. In (*I*) and (*J*), boxplots show median and the interquartile range, and error bars show the full range excluding outliers. Different lowercase letters in indicate a significant difference ($P < 0.05$) according to Duncan's new multiple range test. (*K*) Relative transcript levels of *OsPR1a* and *OsWRKY45* in the indicated genotypes. Gene expression levels were examined and normalized to *OsUBQ*. Values are means ± standard deviation (n = 3). ** indicates significant differences ($P < 0.01$) by two-tailed Student's *t*-test. All experiments were repeated at least three times.

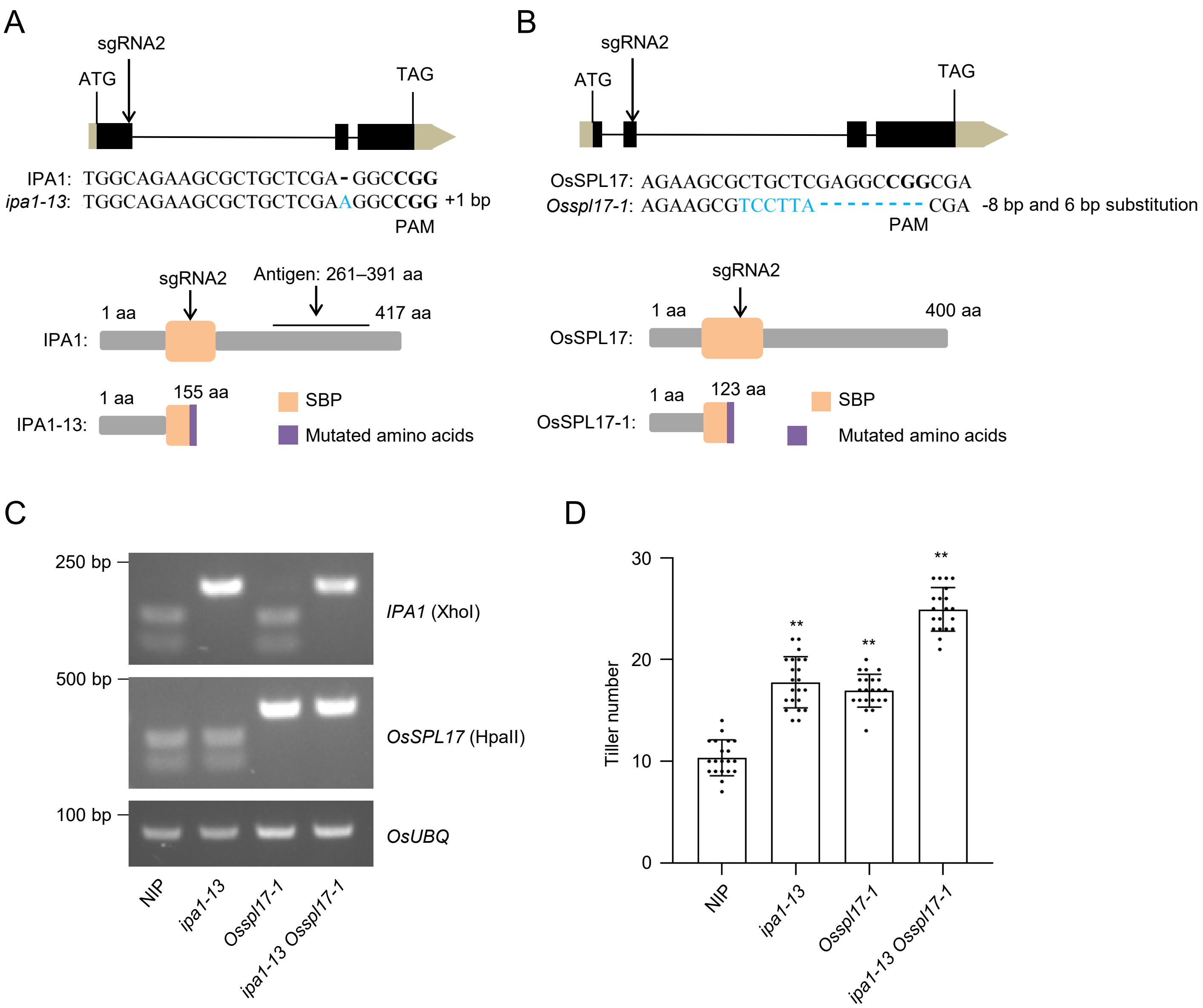

Supplementary Figure S4. Mutations in *IPA1* and *OsSPL17*. (Supports Figure 2)
(*A*) Diagram of the *IPA1* locus with the sgRNA target site for genome editing. The mutation in *IPA1* is shown in blue. Diagram of wild-type IPA1 and the predicted IPA1 mutant protein in the *ipa1-13* mutant. (*B*) Diagram of the *OsSPL17* locus with the sgRNA target site for genome editing. The mutation in *OsSPL17* is shown in blue. Diagram of wild-type OsSPL17 and the predicted OsSPL17 mutant protein in the *Osspl17-1* mutant. (*C*) Genotyping PCR of *ipa1-13*, *Osspl17-1* and *ipa1-13 Osspl17-1* by dCAPS marker in the indicated genotypes. *OsUBQ* served as control. (*D*) Tiller number per plant of *ipa1-13*, *Osspl17-1* and *ipa1-13 Osspl17-1* and NIP (n = 20). ** indicates significant differences ($P < 0.01$) by two-tailed Student's *t*-test.

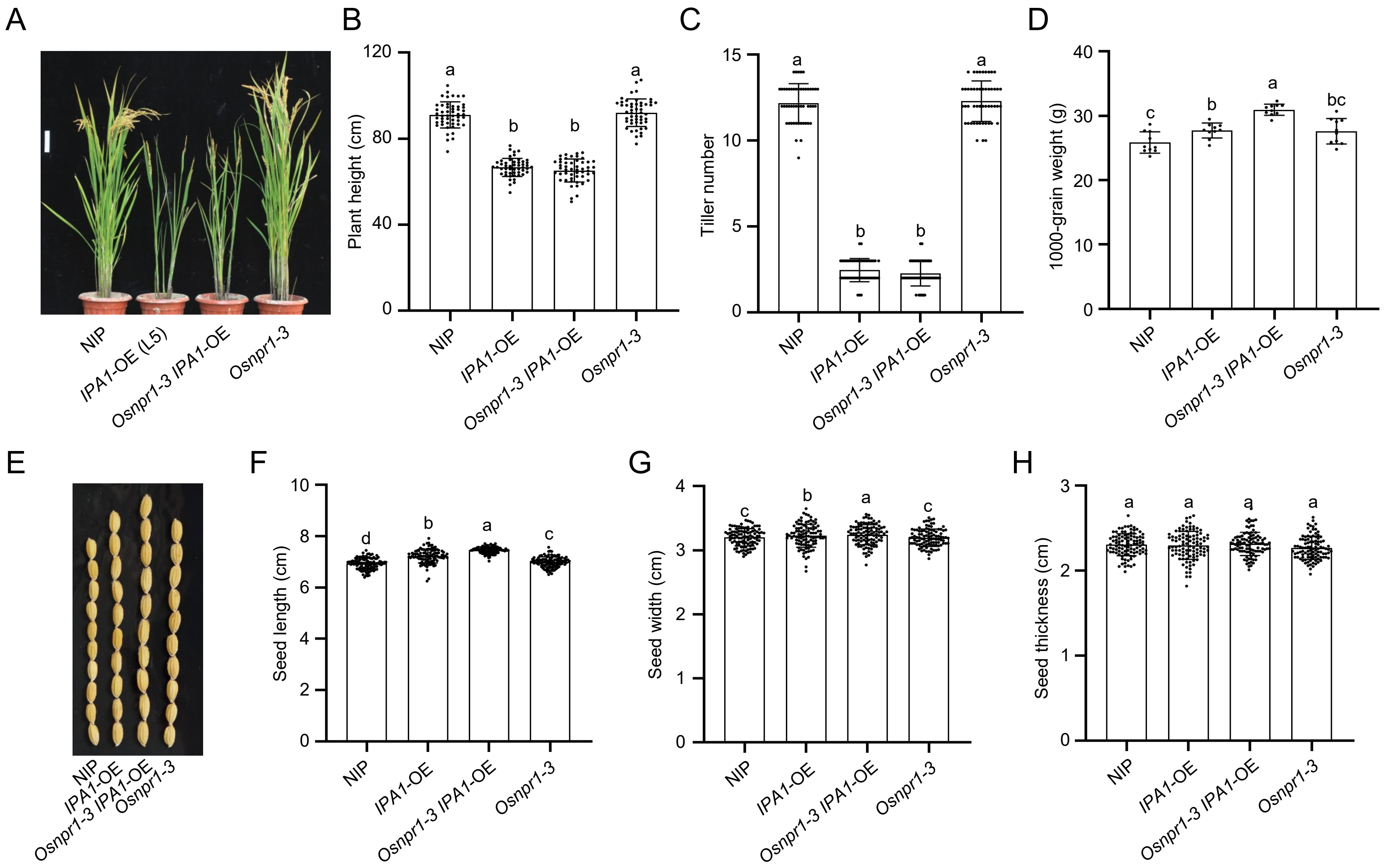

Supplementary Figure S5. IPA1 regulates growth and development in a OsNPR1-independent manner. (Supports Figure 2)

(*A*) Representative photograph of 110-day-old rice plants of the indicated genotypes. (*B*) Height of 110-day-old rice plants of the indicated genotypes (n = 50). (*C*) Tiller number per plant of the indicated genotypes (n = 50). (*D*) 1000-grain weight for the indicated genotypes (n = 10, each sample with 200 grains). (*E*) Grain size for the indicated genotypes. (*F*) Seed length for the indicated genotypes (n = 100). (*G*) Seed width for the indicated genotypes (n = 100). (*H*) Seed thickness for the indicated genotypes (n = 100). Values are means ± standard deviation. Different lowercase letters indicate significant differences ($P < 0.05$) according to Duncan's new multiple range test. All experiments were repeated at least three times.

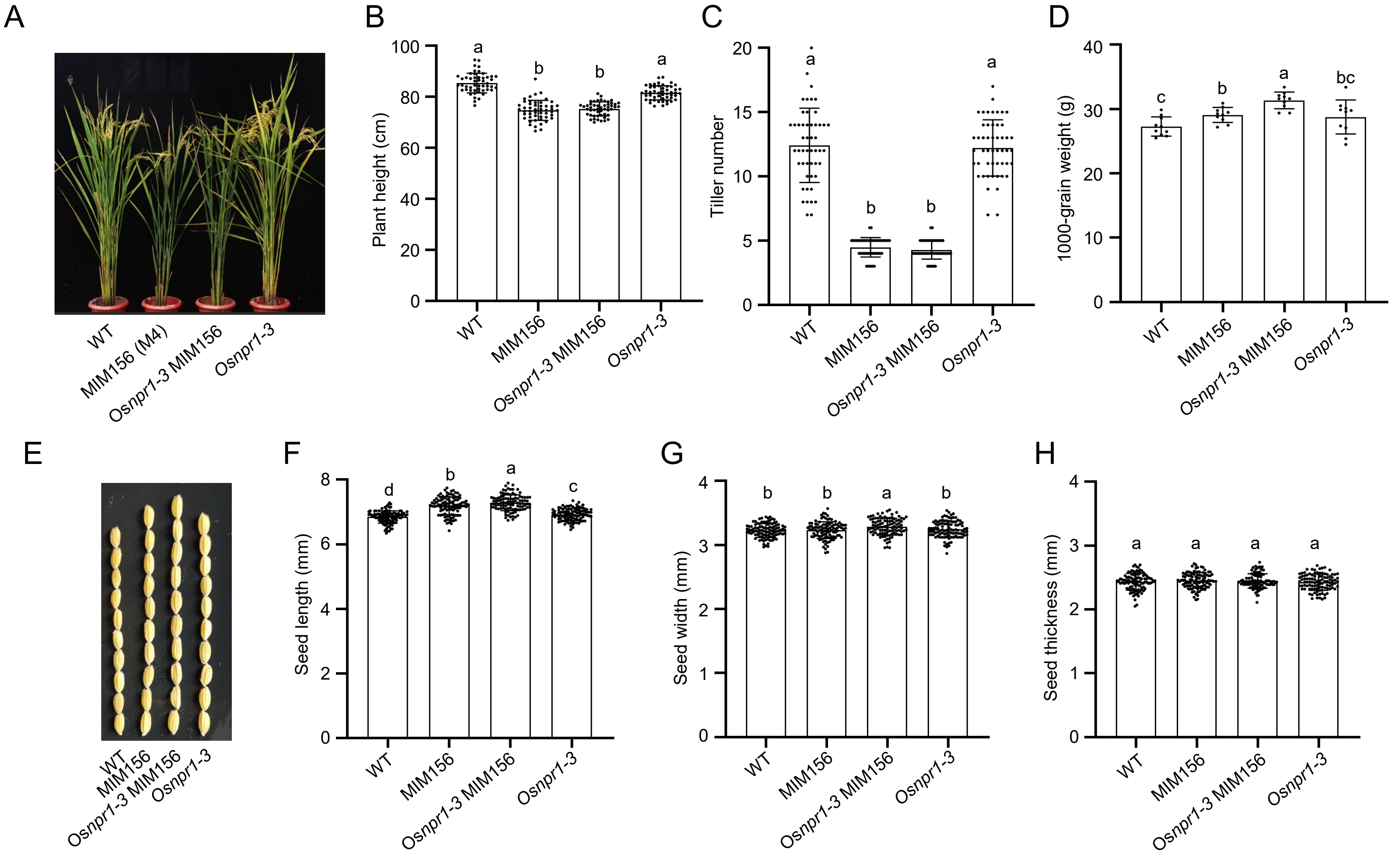

Supplementary Figure S6. OsNPR1 does not contribute to growth or development in MIM156. (Supports Figure 3)

(*A*) Representative photograph of 110-day-old rice plants of the indicated genotypes. (*B*) Plant height of the indicated genotypes (n = 20). (*C*) Tiller number per plant for the indicated genotypes (n = 20). (*D*) 1,000-grain weight for the indicated genotypes (n = 10, each sample with 200 grains). (*E*) Grain size for the indicated genotypes. (*F*) Seed length for the indicated genotypes (n = 100). (*G*) Seed width for the indicated genotypes (n = 100). (*H*) Seed thickness for the indicated genotypes (n = 100). Values are means ± standard deviation. Different lowercase letters indicate significant differences ($P < 0.05$) according to Duncan's new multiple range test. All experiments were repeated at least three times.

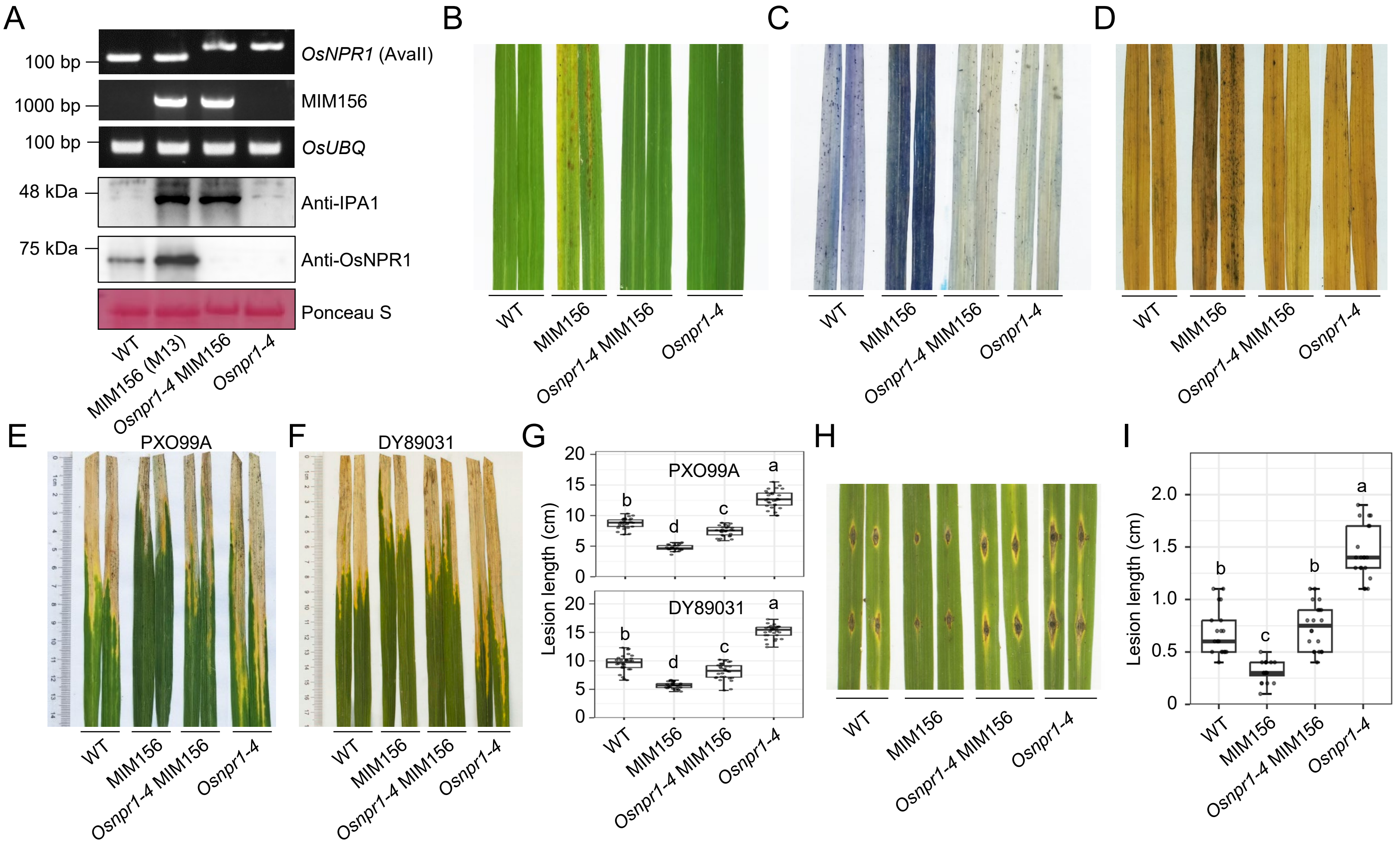

Supplementary Figure S7. OsNPR1 is required for disease resistance in MIM156 rice plants. (Supports Figure 3)

(*A*) Genotyping PCR for the miR156-MIMIC (MIM156) and *Osnpr1-4* by dCAPS in the indicated genotypes; *OsUBQ* served as control (top). Immunoblot analysis of OsNPR1 and IPA1 in the indicated genotypes; Ponceau S staining of the large subunit of Rubisco served as a loading control (bottom). (*B*) Lesion-mimic phenotypes of the indicated genotypes. (*C*) Cell death in the indicated genotypes as determined by trypan blue staining. (*D*) ROS accumulation in the indicated genotypes as shown by DAB staining. (*E*) and (*F*) Lesion symptoms caused by bacterial blight in the indicated genotypes at 14 dpi with *Xoo* strain PXO99A (*E*) and DY89031 (*F*). (*G*) Length of lesions at 14 dpi inoculated with *Xoo* strain PXO99A or DY89031 (n = 30). (*H*) Blast disease symptoms at 7 dpi inoculated with *M. oryzae* strain FJ81278 using the punch-inoculated method. (*I*) Length of lesions at 7 dpi inoculated with strain FJ81278 (n = 20). Boxplots in (*G*) and (*I*) show median and the interquartile range, and error bars denote the full range excluding outliers. Different lowercase letters in (*G*) and (*I*) indicate significant differences ($P < 0.05$) according to Duncan's new multiple range test. All experiments were repeated at least three times.

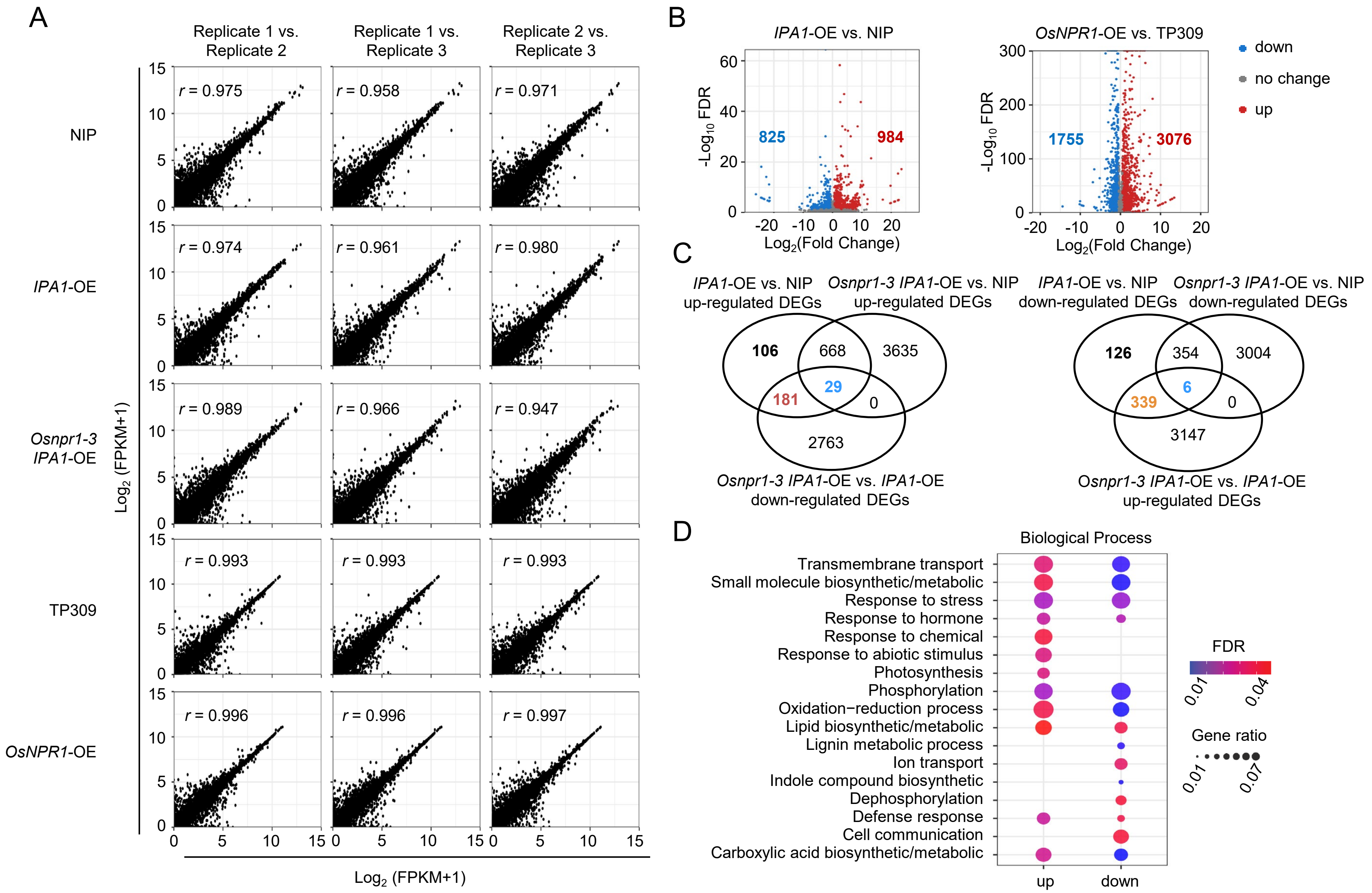

Supplementary Figure S8. Transcriptome analysis of IPA1 and OsNPR1 regulated genes. (Supports Figure 4)

(*A*) Scatterplots showing the extent of pairwise correlation between each of the three biological replicates for each genotype. The Pearson's correlation coefficient (*r* value) is given for each comparison. (*B*) Volcano plots showing the differentially expressed genes (DEGs) in *IPA1*-OE (L5) and *OsNPR1*-OE (G316) compared to their corresponding WT (Log_2(Fold Change) ≥ 0.485; FDR< 0.05). The *x*-axis represents Log_2(Fold Change); the *y*-axis indicates $-Log_{10}$ (FDR). (*C*) Venn diagram showing the extent of overlap among the DEGs obtained from the pairwise comparisons of WT, *IPA1*-OE, and *Osnpr1-3 IPA1*-OE. (*D*) Gene ontology (GO) term enrichment analysis of IPA1-regulated genes that depend on OsNPR1.

A

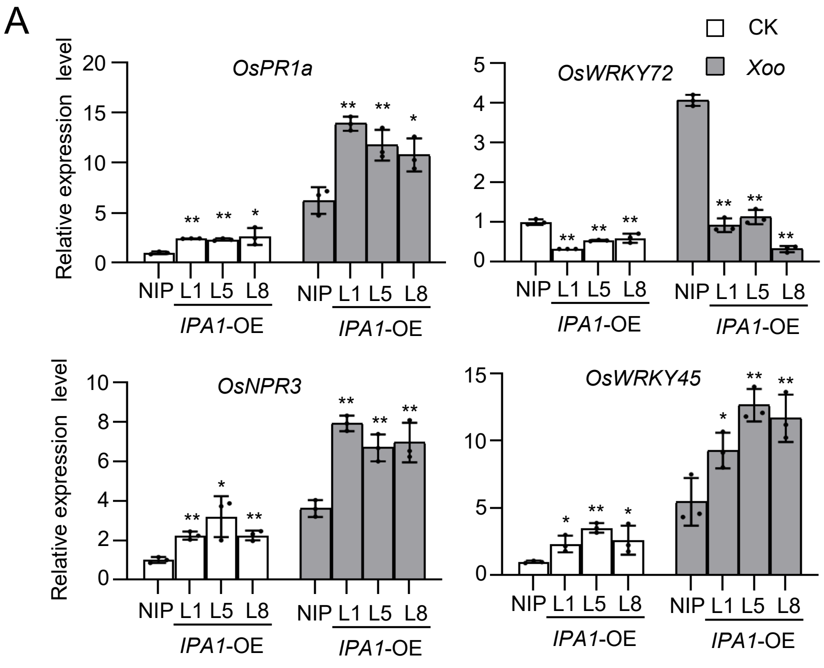

B

Category	Gene Name	Number of IPA1 binding motifs: Total	TGGGCC/T	GTAC
down-regulated in *IPA1*-OE	*ELL1*	4	1	3
	OsNBL3	15	7	8
	OsFd4	11	1	10
	Spl7	11	0	11
	OsSAUR6	16	2	14
	OsWRKY72	12	1	11
up-regulated in *IPA1*-OE	*OsBRR1*	10	2	8
	OsGSTU7	3	2	1
	PR Bet v I	4	0	4
	OsCOMT8	4	0	4
	EUI1	11	1	10
	OsNPR3	17	1	16
	OsWRKY45	15	6	9

C D

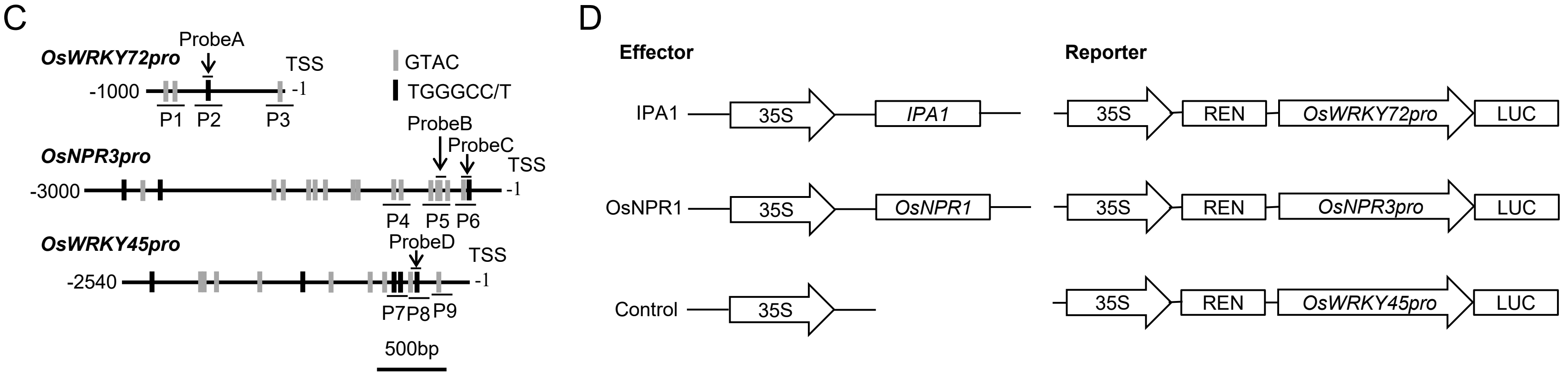

Supplementary Figure S9. Functional analysis of IPA1 downstream genes. (Supports Figure 4)
(*A*) Expression of four *IPA1* regulated genes before and after *Xoo* infection (DY89031). Gene expression levels were examined and normalized to *OsUBQ*. Values are means ± standard deviation (n = 3). Asterisks indicate significant differences (* $P < 0.05$, ** $P < 0.01$) by two-tailed Student's *t*-test. (*B*) Number of IPA1-binding motifs within the 2k bp promoter regions of thirteen IPA1-regulated genes. (*C*) Diagrams showing the IPA1 putative binding motifs in the promoters of *OsWRKY72*, *OsNPR3*, and *OsWRKY45* used in the yeast one-hybrid (Y1H) assay, ChIP-qPCR and EMSA. (*D*) Diagrams showing the effector and reporter constructs used in the dual-luciferase reporter assay.

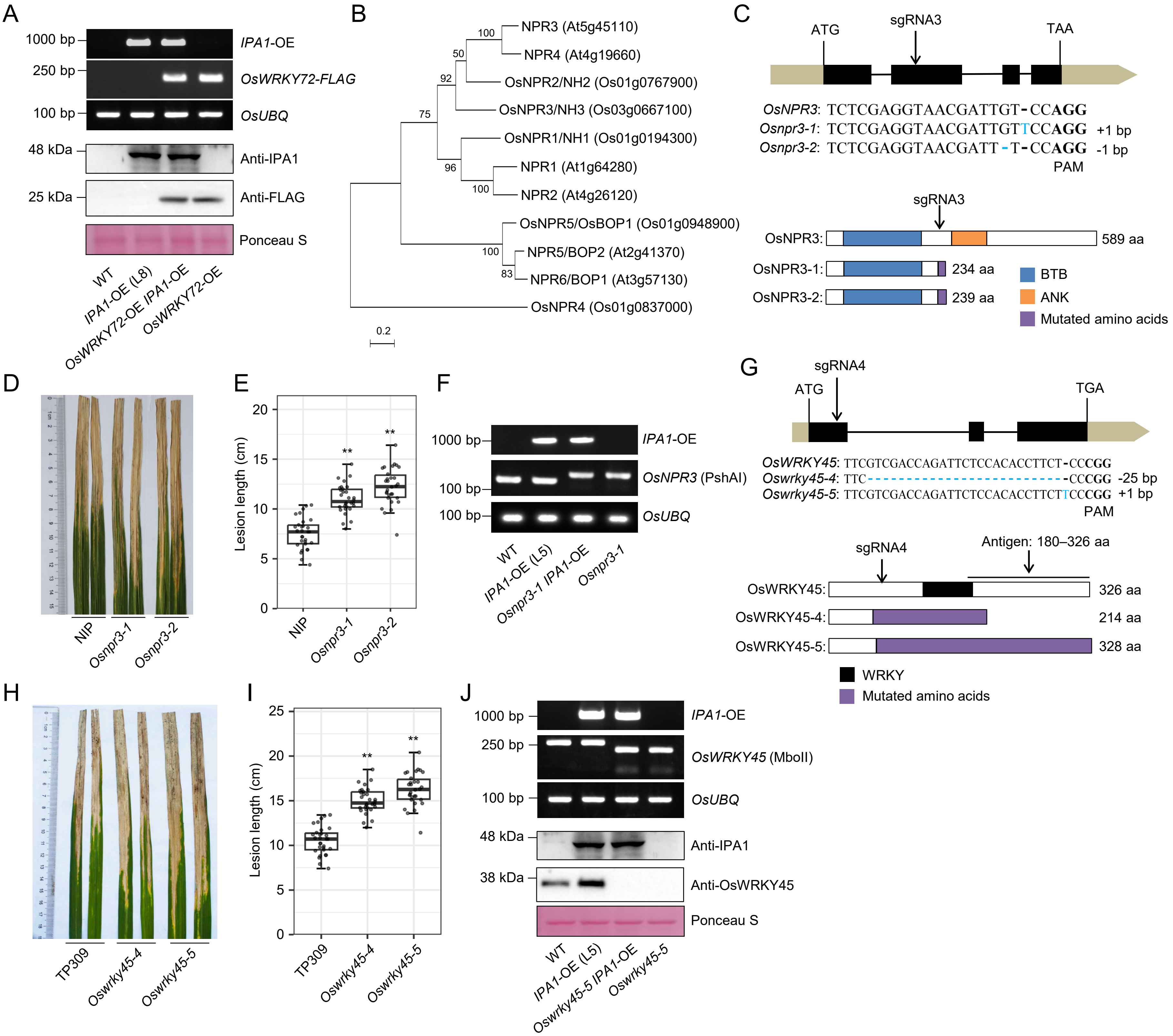

Supplementary Figure S10. Genetic analysis of *IPA1*-OE and its three downstream genes. (Supports Figure 5)

(*A*) Genotyping PCR for the *IPA1*-OE and *OsWRKY72*-OE transgenes in the indicated genotypes; *OsUBQ* served as control (top). Immunoblot analysis of IPA1 and OsWRKY72-FLAG abundance in the indicated genotypes; Ponceau S staining of the large subunit of Rubisco served as a loading control (down). (*B*) Phylogenetic analysis of the NPR proteins in Arabidopsis and rice. MEGA 7.0 software was used to reconstruct the neighbor-joining tree. The scale bar indicates substitutions per site and the number indicates bootstrap values. (*C*) Diagram of the *OsNPR3* locus showing the sgRNA target site. The mutations in the OsNPR3 are shown in blue. Diagram showing the wild-type OsNPR3 and the predicted OsNPR3 proteins in *Osnpr3-1* and *Osnpr3-2* mutants. (*D*) Lesion symptoms by bacterial blight in the indicated genotypes at 14 dpi inoculated with *Xoo* strain PXO99A. (*E*) Length of Lesions in the indicated genotypes at 14 dpi inoculated with *Xoo* PXO99A (n = 30). (*F*) Genotyping for the *IPA1*-OE transgene and the *Osnpr3-1* mutation by dCAPS marker. *OsUBQ* served as control. (*G*) Diagram of the *OsWRKY45* locus showing the sgRNA target site. The mutations in *OsWRKY45* in *Oswrky45-4* and *Oswrky45-5* are shown in blue. Diagram showing the structure of wild-type OsWRKY45 and the predicted mutated OsWRKY45 proteins in *Oswrky45-4* and *Oswrky45-5* mutants. (*H*) Lesion symptoms in the indicated genotypes at 14 dpi inoculated with *Xoo* strain PXO99A. (*I*) The length of lesions in the indicated genotypes at 14 dpi inoculated with *Xoo* PXO99A (n = 30). (*J*) Genotyping PCR for the *IPA1*-OE transgene and the *Oswrky45-5* mutation by dCAPS marker; *OsUBQ* served as control. Immunoblot analysis of OsWRKY45 and IPA1 in the indicated genotypes. Ponceau S staining of the large subunit of Rubisco served as a loading control. Boxplots in (*E*) and (*I*), show median and interquartile range, and error bars denote the full range excluding outliers. In (*E*) and (*I*), ** indicates significant differences ($P < 0.01$) by two-tailed Student's *t*-test.

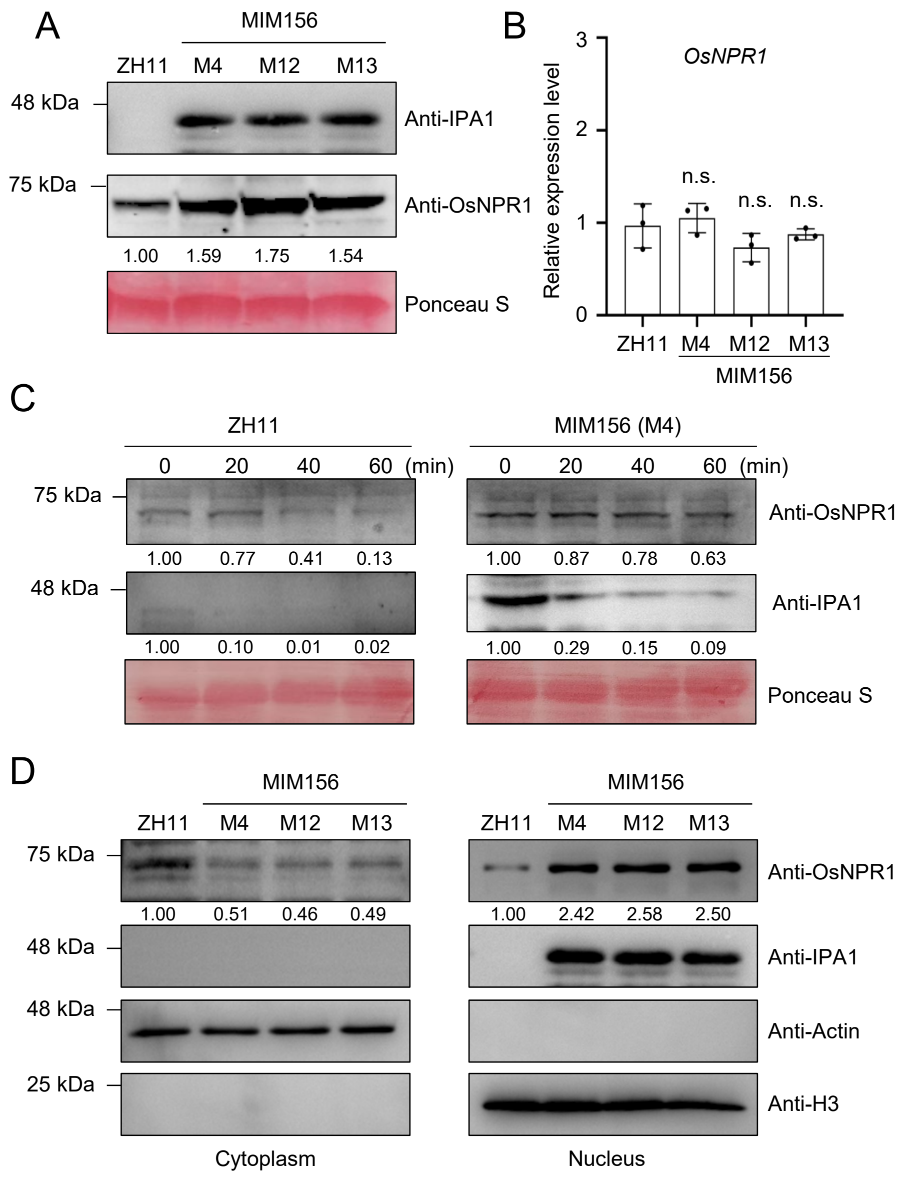

Supplementary Figure S11. OsNPR1 and IPA1 abundance in MIM156 plants. (Supports Figure 6)

(*A*) Immunoblot analysis of OsNPR1 and IPA1 in leaves of the wild-type ZH11 and three MIM156 lines. Ponceau S staining of the large subunit of Rubisco served as a loading control. (*B*) Relative OsNPR1 transcript levels in ZH11 and three MIM156 lines as determined by qRT-PCR. *OsNPR1* gene expression levels were examined and normalized to *OsUBQ*. Values are means ± standard deviation (n = 3). n.s. indicates non-significant differences (P > 0.05) according to two-tailed Student's *t*-test. (*C*) Immunoblot analysis of OsNPR1 and IPA1 in ZH11 and MIM156 (M4) at the indicated time points in the degradation assay. Ponceau S staining of the large subunit of Rubisco served as a loading control. (*D*) Subcellular fractionation assay showing the abundance of OsNPR1 and IPA1 in the cytoplasm and nucleus from ZH11 and MIM156 plants. Actin and histone H3 served as markers for the cytosolic and nuclear fractions, respectively. All experiments were repeated at least three times.

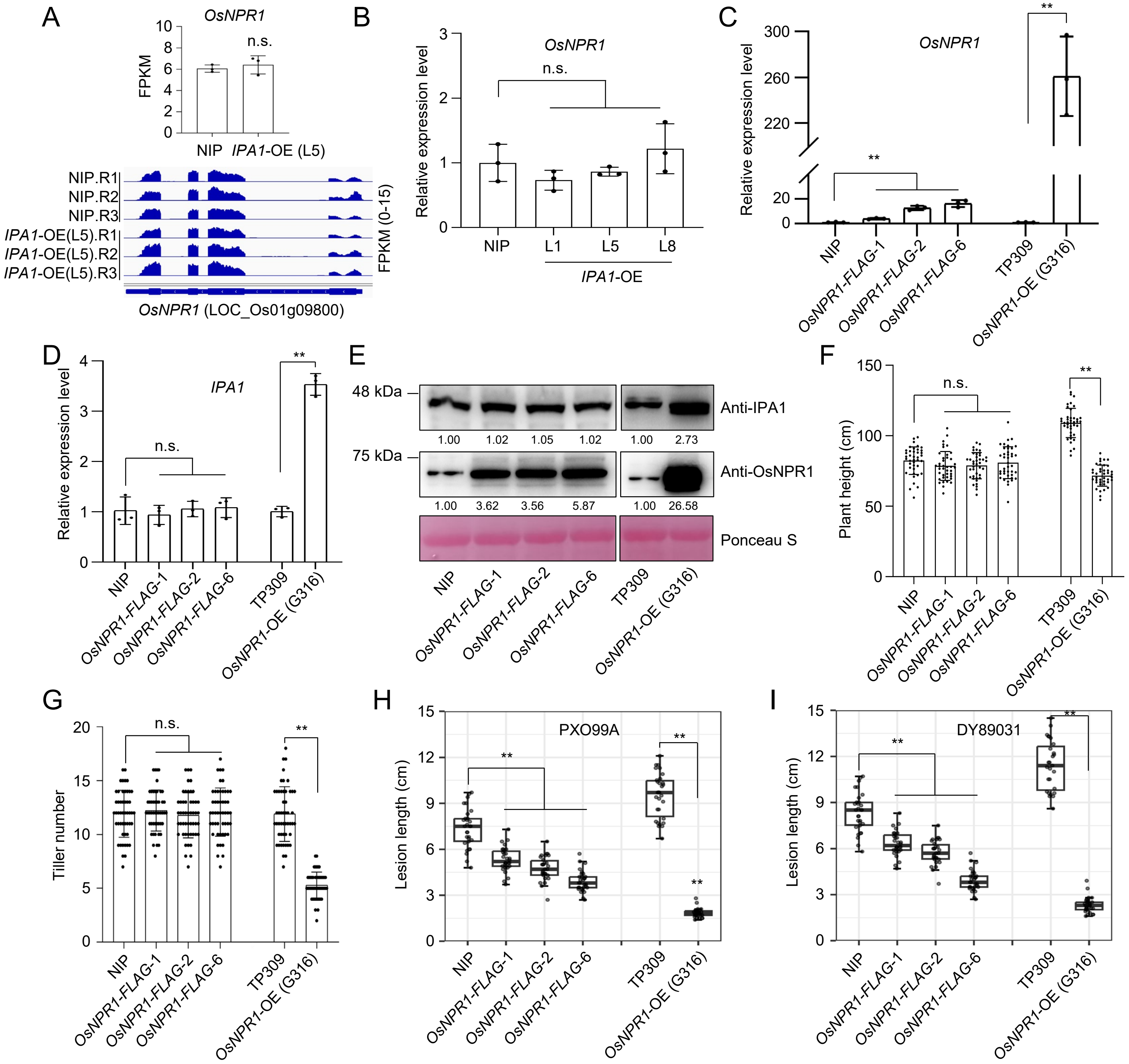

Supplementary Figure S12. The effect of OsNPR1 abundance on expression levels of IPA1. (Supports Figure 6)

(*A*) The transcript levels of *OsNPR1* in *IPA1*-OE (L5) compared to that in NIP based on RNA-seq analysis. (B) Relative *OsNPR1* transcript levels in NIP and three *IPA1*-OE lines as determined by qRT-PCR. (C) The relative transcript levels of *OsNPR1* in the indicated genotypes (n = 3). (D) The relative transcript levels of *IPA1* in the indicated genotypes. In (*B*), (*C*) and (*D*), gene expression levels were examined and normalized to *OsUBQ*, values are means ± standard deviation (n = 3). (*E*) Immunoblot analysis of OsNPR1 and IPA1 protein abundance in the indicated genotypes. Ponceau S staining of the large subunit of Rubisco served as a loading control. (*F*) The plant height of indicated genotype (n = 50). (*G*) The tiller number per plant of indicated genotype (n = 50). (*H*) Length of lesions at 14 dpi inoculated with *Xoo* PXO99A (n = 30). (*I*) Length of lesions at 14 dpi inoculated with *Xoo* DY89031 (n = 30). Boxplots in (*H*) and (*I*) show median and interquartile range, and error bars denote the full range excluding outliers. ** indicates significant differences (P < 0.01); n.s. indicates non-significant differences (P > 0.05) according to two-tailed Student's *t*-test. All experiments were repeated at least three times.

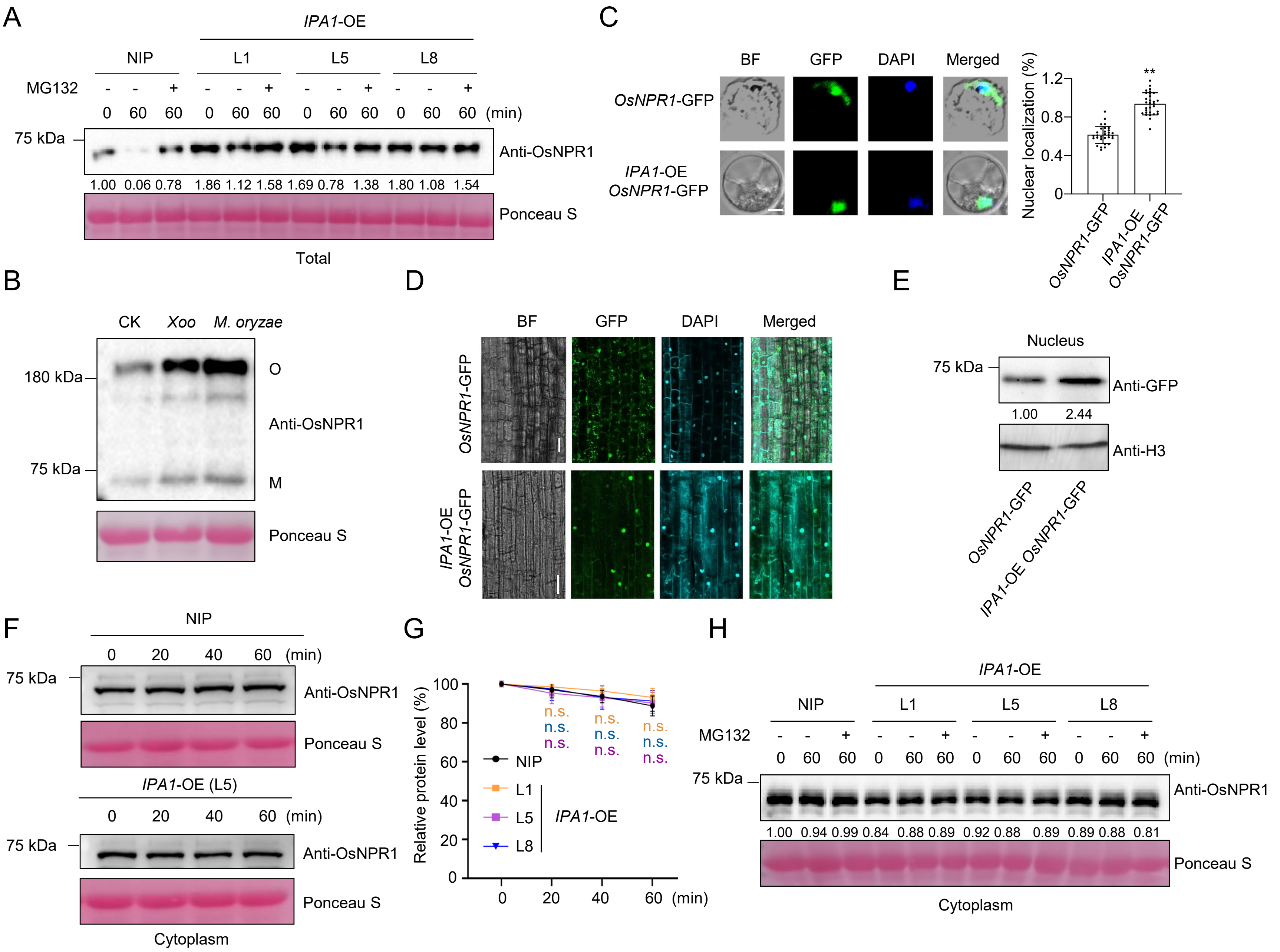

Supplementary Figure S13. IPA1- or pathogen-induced changes in the subcellular localization or oligomer–monomer abundance of OsNPR1, and the effect of MG132 on its stability. (Supports Figure 6)

(*A*) Immunoblot analysis of OsNPR1 abundance at the indicated time points with or without MG132 treatment. Ponceau S staining of the large subunit of Rubisco served as a loading control. (*B*) Immunoblot analysis of monomeric and oligomeric forms of OsNPR1 under non-reducing conditions in NIP plants after inoculation with *Xoo* (left) or *M. oryzae* (right). (*C*) Subcellular localization of *OsNPR1*-GFP in protoplasts of NIP and *IPA1*-OE. The ratio of nuclear localization was quantified in NIP and *IPA1*-OE (n = 50). BF, bright field. Scale bar = 10 μm. (*D*) Confocal scanning images for *OsNPR1-GFP* and *IPA1*-OE *OsNPR1-GFP* plants. Scale bar = 50 μm. (*E*) Immunoblot analysis of nuclear OsNPR1-GFP. Histone H3 was used as a nuclear loading control. (*F*) Immunoblot analysis of cytoplasmic OsNPR1 in NIP and *IPA1*-OE (L5) at the indicated time points, based on subcellular fractionation of total protein extracts. Ponceau S staining of the large subunit of Rubisco served as a loading control. (*G*) Quantification of relative abundance of cytoplasmic OsNPR1 in NIP and three *IPA1*-OE lines at the indicated time points. (*H*) Immunoblot analysis of cytoplasmic OsNPR1 in NIP and three *IPA1*-OE lines at the indicated time points with or without MG132 treatment, based on subcellular fractionation. Ponceau S staining of the large subunit of Rubisco served as a loading control. In (*C*) and (*G*), ** indicates significant differences ($P < 0.01$); n.s. indicates non-significant differences ($P > 0.05$) according to two-tailed Student's *t*-test. All experiments were repeated at least three times.

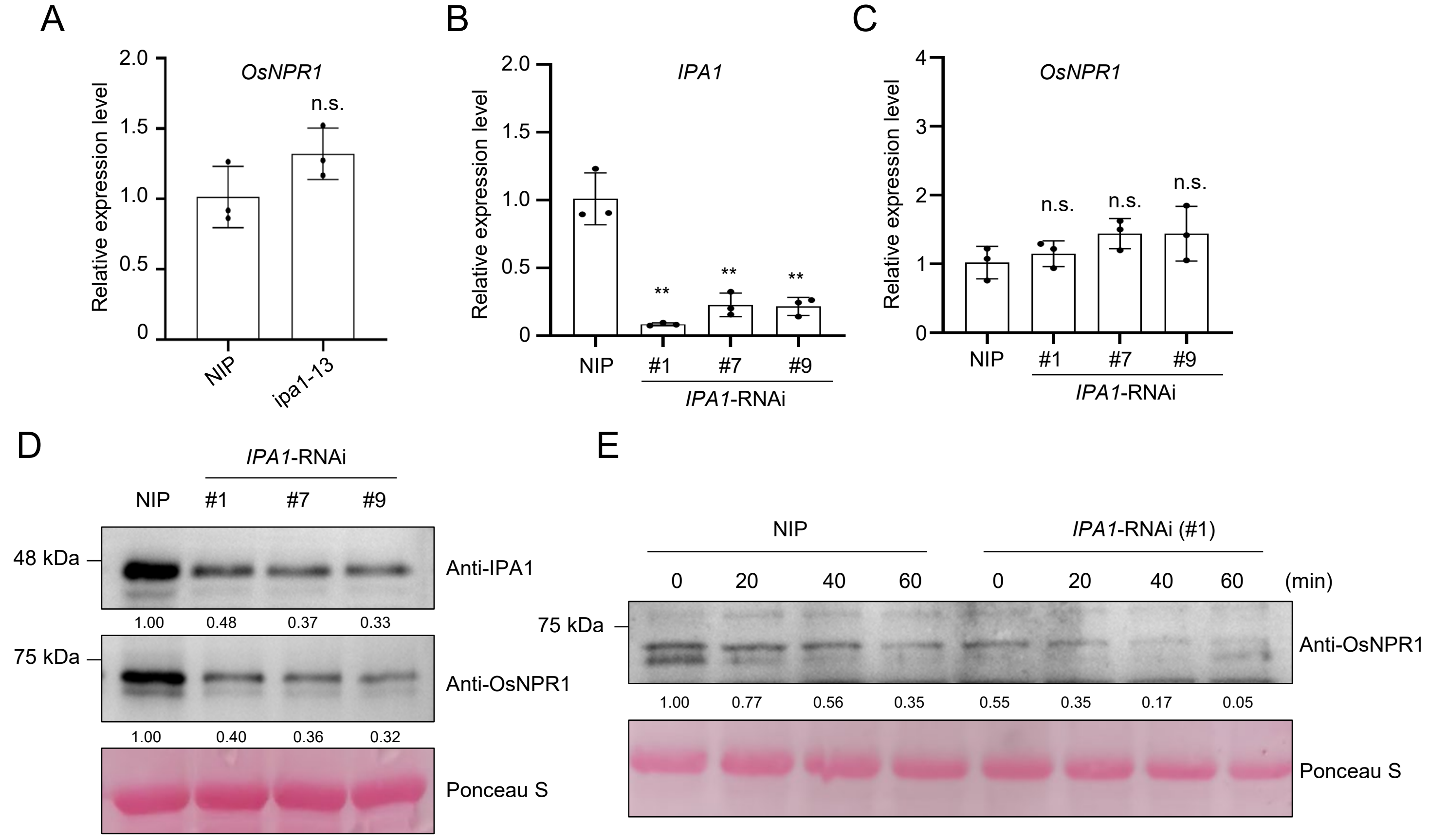

Supplementary Figure S14. The protein stability of OsNPR1 in *IPA1*-RNAi transgenic rice plants. (Supports Figure 6)

(*A*) Relative *OsNPR1* transcript levels in NIP and *ipa1-13* as determined by qRT-PCR. (*B* and *C*) Relative *IPA1* (*B*) and *OsNPR1* (*C*) transcript levels in NIP and *IPA1*-RNAi as determined by qRT-PCR. In (*A*), (*B*) and (*C*), gene expression levels were examined and normalized to *OsUBQ*, values are means ± standard deviation (n = 3). In (*A*), (*B*) and (*C*), ** indicates significant differences ($P < 0.01$), n.s. indicates non-significant differences ($P > 0.05$) according to two-tailed Student's *t*-test. (*D*) Immunoblot analysis of OsNPR1 and IPA1 abundance in two-month-old leaves of three *IPA1*-RNAi rice lines. Ponceau S staining of the large subunit of Rubisco served as a loading control. (*E*) Immunoblot analysis of OsNPR1 in NIP and *IPA1*-RNAi (#1) at the indicated time points in the degradation assay. Ponceau S staining served as a loading control.

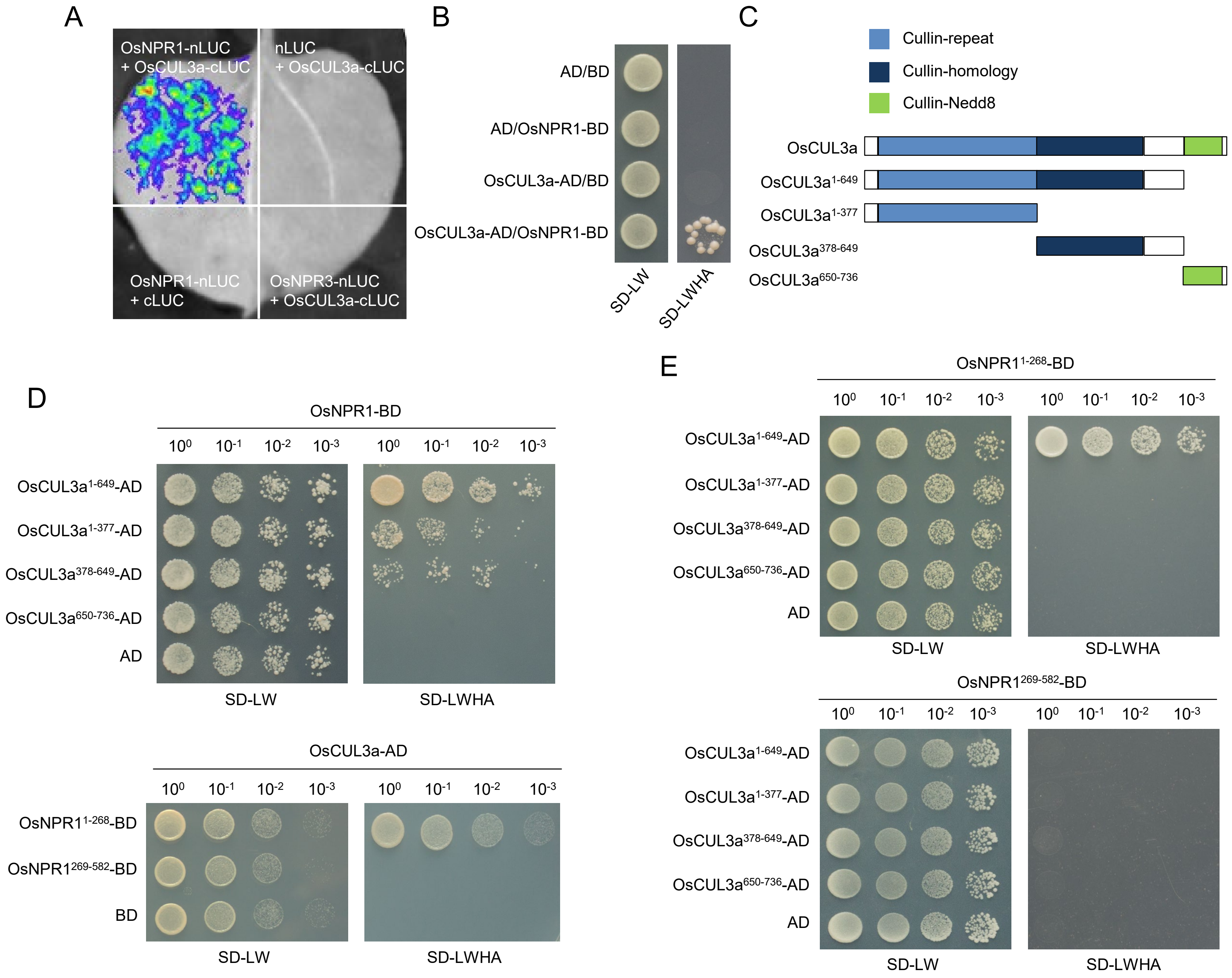

Supplementary Figure S15. OsCUL3a physically interacts with OsNPR1. (Supports Figure 7)

(*A*) LUC complementation assay showing that OsCUL3a interacts with OsNPR1 but not with OsNPR3 in *N. benthamiana* leaves. (*B*) Y2H assay showing that OsCUL3a and OsNPR1 interact. (*C*) Diagrams of the full-length and truncated OsCUL3a constructs used in Y2H assays. (*D*) Y2H assay testing the interaction between various truncated versions of OsCUL3a and full-length OsNPR1 (top) and between full-length OsCUL3a and truncated forms of OsNPR1 (bottom). (*E*) Y2H assay between various truncated versions of OsCUL3a and two truncated versions of OsNPR1. All experiments were repeated at least three times.

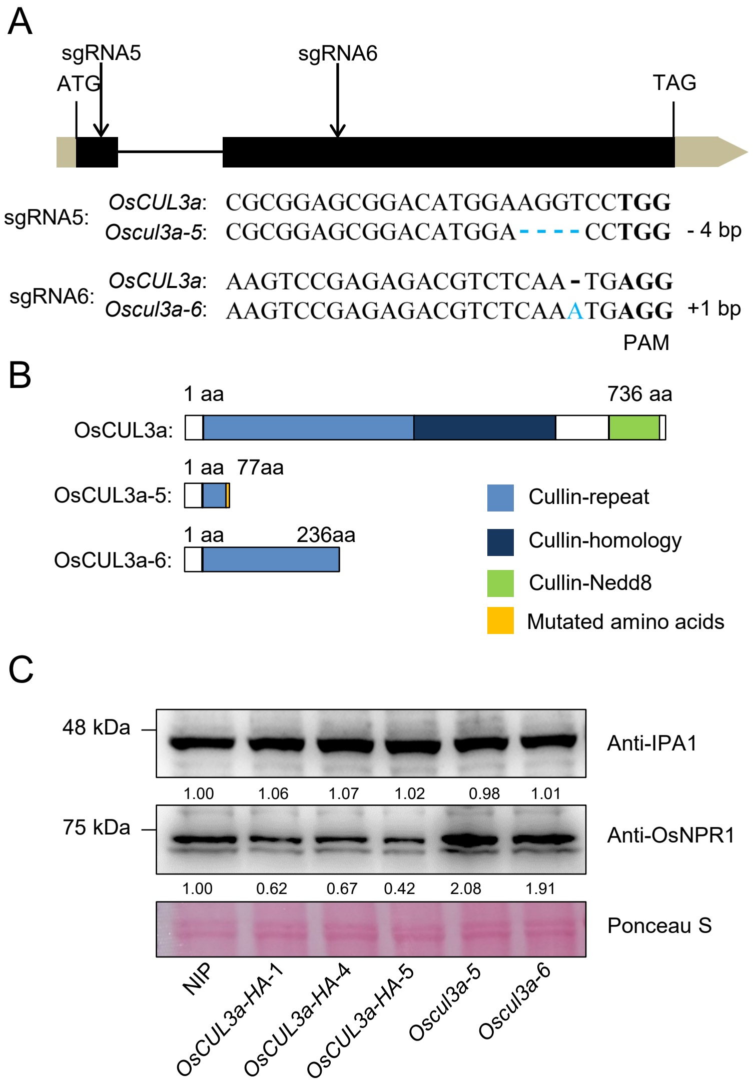

Supplementary Figure S16. OsCUL3a does not affect the protein levels of IPA1. (Supports Figure 7)

(*A*) Diagram of the *OsCUL3a* locus with two sgRNA target sites for genome editing. The mutations in *OsCUL3a* are shown in blue. (*B*) Diagram of wild-type OsCUL3a and the predicted OsCUL3a mutant proteins in the *Oscul3a-5* and *Oscul3a-6* mutants. (*C*) Immunoblot analysis of OsNPR1 and IPA1 in two-month-old leaves of *OsCUL3a-HA* and *Oscul3a* mutant plants, Ponceau S staining served as a control. This experiment was repeated at least three times.

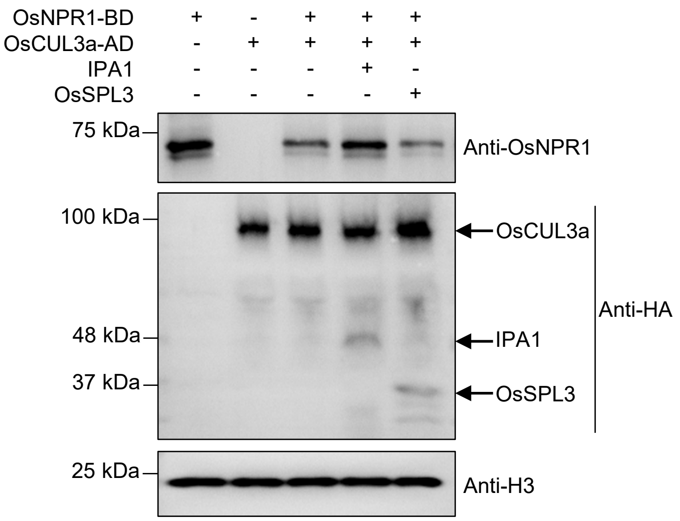

Supplementary Figure S17. The protein levels of OsNPR1, OsCUL3a, IPA1, and OsSPL3 in yeast three-hybrid. (Supports Figure 7)

The immunoblotting analysis of OsCUL3a, IPA1, and OsSPL3 using anti-HA antibody, and of OsNPR1 using anti-OsNPR1 antibody.

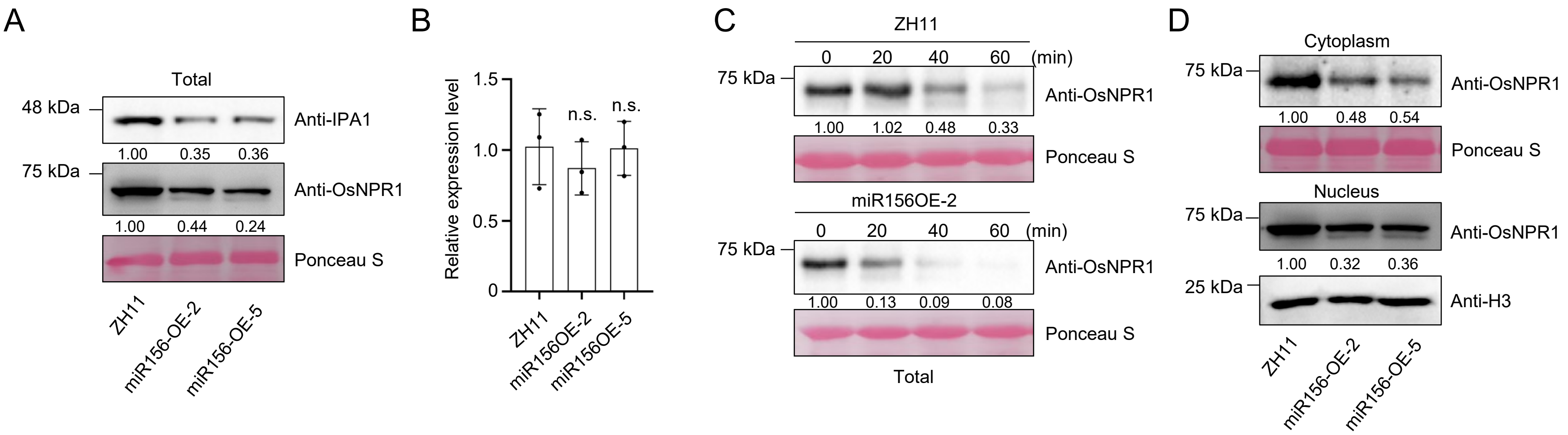

Supplementary Figure S18. miR156 overexpression leads to OsNPR1 protein instability. (Supports Figure 7)

(*A*) Immunoblot analysis the protein abundance of IPA1 and OsNPR1 in ZH11 and miR156-OE two-month-old leaves. Ponceau S staining of the large subunit of Rubisco served as a loading control. (*B*) Relative *OsNPR1* transcript levels in ZH11 and miR156-OE as determined by qRT-PCR. *OsNPR1* gene expression levels were examined and normalized to *OsUBQ*. Values are means ± standard deviation (n = 3). n.s. indicates non-significant differences ($P > 0.05$) according to two-tailed Student's *t*-test. (*C*) Immunoblot analysis of OsNPR1 abundance in ZH11 and miR156-OE at the indicated time points in cell-free degradation assay. Ponceau S staining of the large subunit of Rubisco served as a loading control. (*D*) Immunoblot analysis of OsNPR1 following subcellular fractionation from ZH11 and miR156-OE total proteins. Anti-histone H3 served as a loading control for the nucleus; Ponceau S staining served as a loading control for the cytoplasm. All experiments were repeated at least three times.

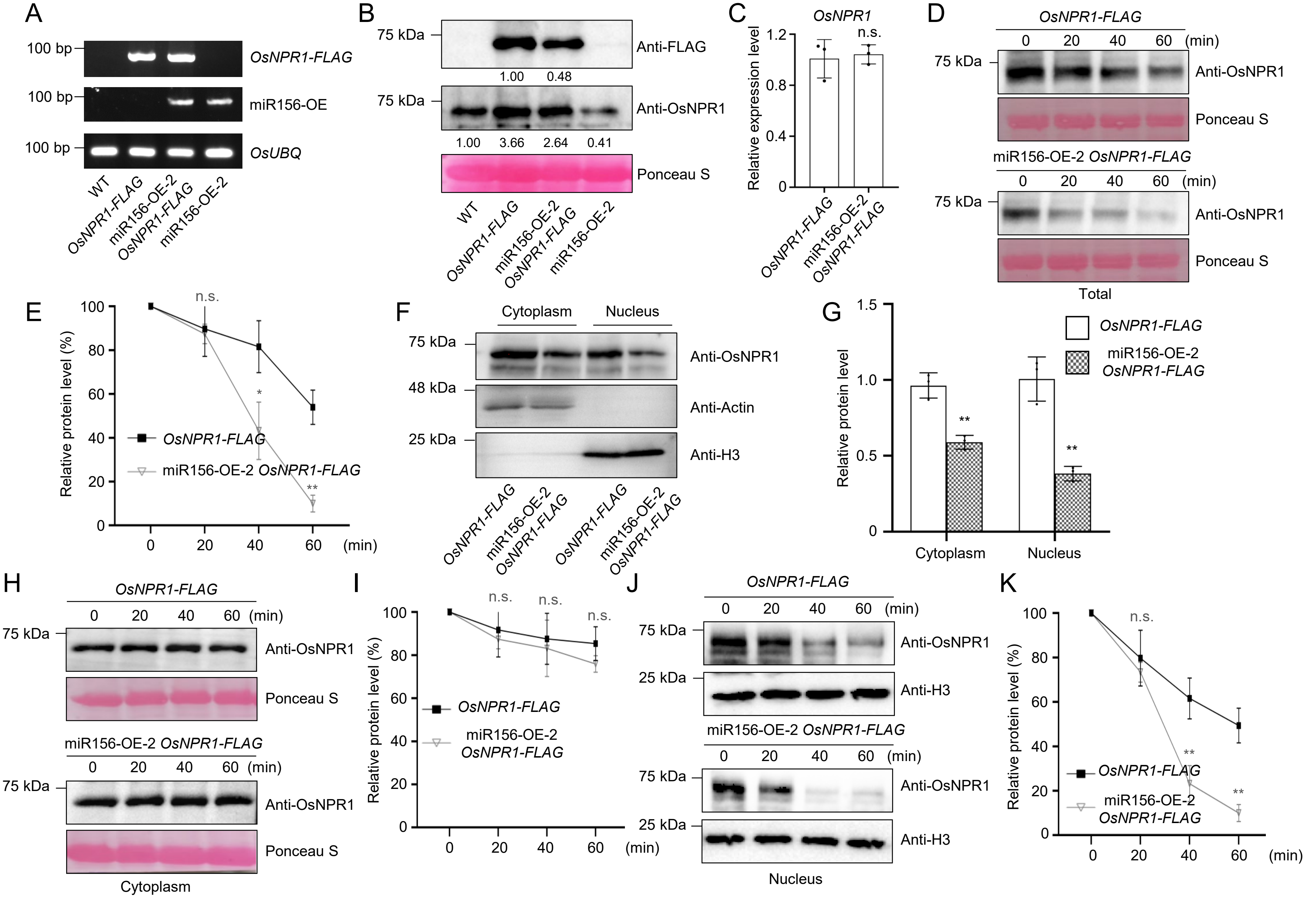

Supplementary Figure S19. miR156 overexpression leads to OsNPR1 protein instability in the nucleus. (Supports Figure 7)

(*A*) Genotyping PCR for *OsNPR1-FLAG* and miR156-OE transgenes in the indicated genotypes; *OsUBQ* served as the control. (*B*) Immunoblot analysis of the protein abundance of OsNPR1, and OsNPR1-FLAG in miR156-OE *OsNPR1-FLAG* and *OsNPR1-FLAG* plants. Ponceau S staining of the large subunit of Rubisco served as a loading control. (*C*) Relative *OsNPR1* transcript levels in miR156-OE *OsNPR1-FLAG* and *OsNPR1-FLAG* as determined by qRT-PCR. *OsNPR1* gene expression levels were examined and normalized to *OsUBQ*. Values are means ± standard deviation (n = 3). (*D*) Cell-free degradation assay for OsNPR1 in miR156-OE *OsNPR1-FLAG* and *OsNPR1-FLAG*. Ponceau S staining of the large subunit of Rubisco served as a loading control. (*E*) Quantification of OsNPR1 abundance at the indicated time points in *OsNPR1-FLAG* and miR156-OE *OsNPR1-FLAG* based on three biological replicates. (*F*) Subcellular fraction assay showing the abundance of OsNPR1 in the cytoplasmic and nucleus fractions for the indicated genotypes. (*G*) Quantification of relative OsNPR1 abundance in the cytoplasm and nucleus for the indicated genotypes. (*H*) and (*I*) Cell-free degradation assay for cytoplasm-localized OsNPR1 in the indicated genotypes at the indicated time points (*H*) and quantification of relative OsNPR1 abundance at the indicated time points (*I*). Ponceau S staining of the large subunit of Rubisco served as a loading control. (*J*) and (*K*) Degradation assay for nucleus-localized OsNPR1 in the indicated genotypes (*J*) and quantification of relative OsNPR1 abundance (*K*). Histone H3 served as loading control for the nucleus. Ponceau S staining served as loading control for the cytoplasm. In (*C*), (*E*), (*G*), (*I*) and (*K*), values are means ± standard deviation, asterisks indicate significant differences (* $P < 0.05$, ** $P < 0.01$), n.s. indicates non-significance ($P > 0.05$) according to two-tailed Student's *t*-test. All experiments were repeated at least three times.

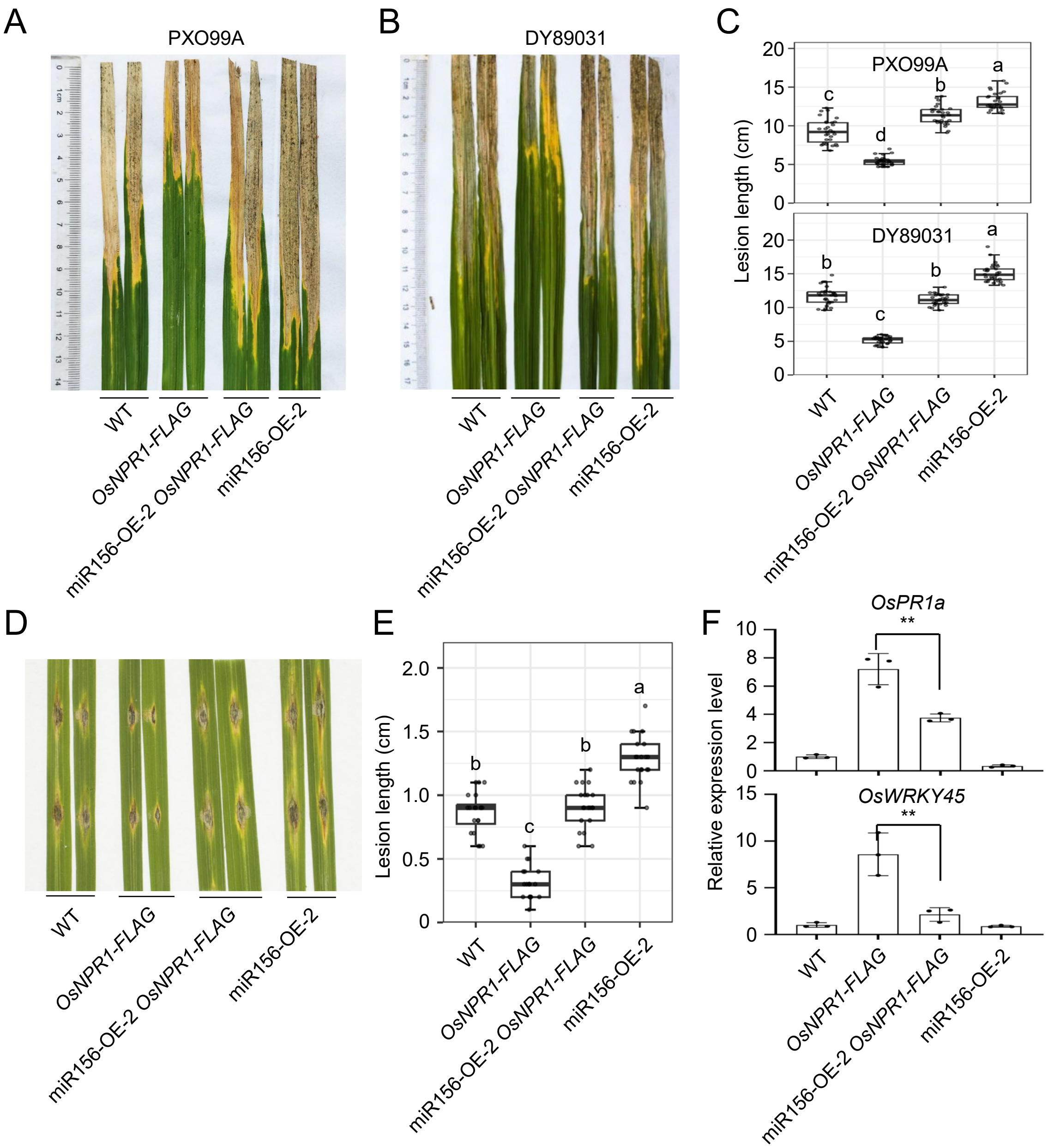

Supplementary Figure S20. miR156 overexpression compromises OsNPR1-mediated disease resistance. (Supports Figure 7)

(*A*) and (*B*) Disease symptoms of the indicated genotypes at 14 dpi inoculated with *Xoo* strain PXO99A (*A*) and DY89031 (*B*). (*C*) The length of lesions for the indicated genotypes at 14 dpi inoculated with PXO99A or DY89031 (n = 30). (*D*) Disease symptoms of the indicated genotypes at 7 dpi inoculated with *M. oryzae* strain FJ81278 by the punch-inoculated method. (*E*) Length of lesions at 7 dpi inoculated with *M. oryzae* strain FJ81278 (n = 20). (*F*) Relative transcript levels of *OsPR1a* and *OsWRKY45* in the indicated genotypes. Gene expression levels were examined and normalized to *OsUBQ*. Values are means ± standard deviation (n = 3). ** indicates significant differences ($P < 0.01$) according to two-tailed Student's *t*-test. Boxplots in (*C*) and (*E*) show median and interquartile range, and error bars denote the full range excluding outliers. Different lowercase letters indicate a significant difference ($P < 0.05$) according to Duncan's new multiple range test. All experiments were repeated at least three times.

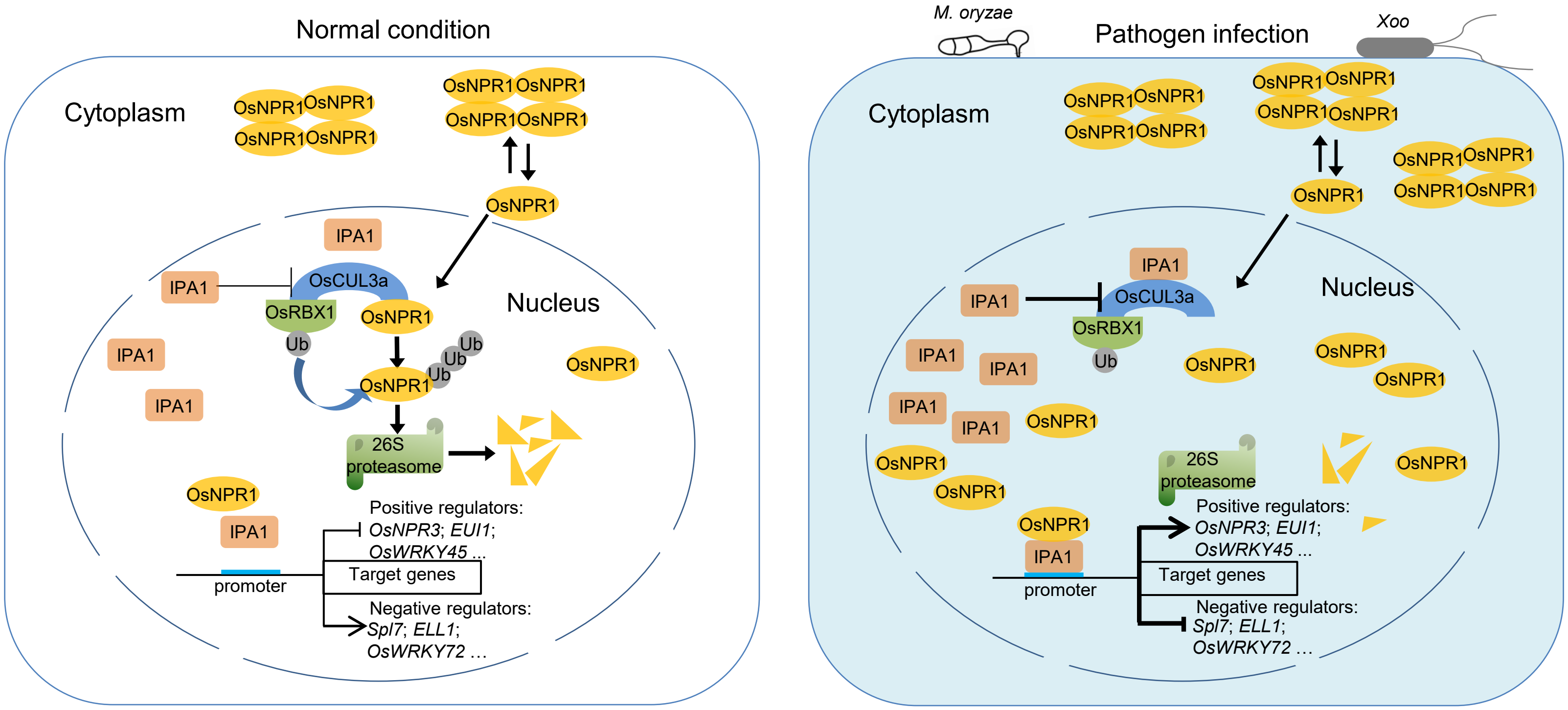

Supplementary Figure S21. IPA1-OsNPR1 mutual potentiation under pathogen infection. (Supports Figures 1–7 and Supplementary Figures S1–S20)

In normal growth conditions, a low IPA1 abundance has little effect on the interaction between OsNPR1 and OsCUL3a, allowing OsCUL3a to degrade OsNPR1 via the 26S proteasome system, resulting in a low OsNPR1 abundance in the nucleus. In rice plants, defense is turned off due to a low abundance of the IPA1-OsNPR1 protein complex. Under pathogen infection, the protein levels of IPA1 and OsNPR1 both become elevated. OsNPR1, as a transcriptional co-activator of IPA1, switches from oligomer to monomer, and translocates into the nucleus where it interacts with IPA1 and facilitates IPA1 for transcription activity. In turn, the abundant IPA1 physically interacts with OsCUL3a and interferes with OsCUL3a-OsNPR1 interaction, ultimately stabilizing OsNPR1 in the nucleus.
